# A small molecule PI3Kα activator for cardioprotection and neuroregeneration

**DOI:** 10.1038/s41586-023-05972-2

**Published:** 2023-05-24

**Authors:** Grace Q Gong, Benoit Bilanges, Ben Allsop, Glenn R Masson, Victoria Roberton, Trevor Askwith, Sally Oxenford, Ralitsa R Madsen, Sarah E Conduit, Dom Bellini, Martina Fitzek, Matt Collier, Osman Najam, Zhenhe He, Ben Wahab, Stephen H McLaughlin, AW Edith Chan, Isabella Feierberg, Andrew Madin, Daniele Morelli, Amandeep Bhamra, Vanesa Vinciauskaite, Karen E. Anderson, Silvia Surinova, Nikos Pinotsis, Elena Lopez-Guadamillas, Matthew Wilcox, Alice Hooper, Chandni Patel, Maria A Whitehead, Tom D Bunney, Len R Stephens, Phillip T Hawkins, Matilda Katan, Derek M Yellon, Sean M Davidson, David M Smith, James B Phillips, Richard Angell, Roger L Williams, Bart Vanhaesebroeck

**Affiliations:** 1 Cell Signalling, Cancer Institute, University College London, UK; 2 Drug Discovery Group, Translational Research Office, University College London, London, UK; 3 Medical Research Council Laboratory of Molecular Biology, Cambridge, UK; 4 Division of Cellular Medicine, School of Medicine, University of Dundee, UK; 5 UCL Centre for Nerve Engineering, UCL School of Pharmacy, University College London, London, UK; 6 Hit Discovery, Discovery Sciences, R&D, AstraZeneca, Alderley Park, Cheshire, UK; 7 The Hatter Cardiovascular Institute, University College London, London, UK; 8 Medicines Discovery Institute, School of Biosciences, Cardiff University, Cardiff CF10 3AT, UK; 9 Wolfson Institute for Biomedical Research, University College London, London, UK; 10 Molecular AI, Discovery Sciences, R&D, AstraZeneca, Waltham MA, USA; 11 Hit Discovery, Discovery Sciences, R&D, AstraZeneca, Cambridge, UK; 12 Proteomics Research Translational Technology Platform, Cancer Institute, University College London, London, UK; 13 Signalling Programme, Babraham Institute, Cambridge, UK; 14 Institute of Structural and Molecular Biology, Birkbeck College, London, UK; 15 Institute of Structural and Molecular Biology, Division of Biosciences, University College London, London, UK; 16 Emerging Innovations, Discovery Sciences, R&D, AstraZeneca, Cambridge, UK

**Keywords:** PI3K, PI3Kα, *PIK3CA*, kinase, small molecule, allosteric, activator, regeneration, drug development, cell protection, modulator, signalling, HDX-MS, crystallography, neuron, cardiac, heart, ischaemia reperfusion injury

## Abstract

Harnessing the potential beneficial effects of kinase signalling through the generation of direct kinase activators remains an underexplored area of drug development^1–5^. This also applies to the PI 3-kinase (PI3K) signalling pathway, which has been extensively targeted by inhibitors for conditions with PI3K overactivation, such as cancer and immune dysregulation. Here we report on the discovery of UCL-TRO-1938 (further referred to as 1938), a small molecule activator of the PI3Kα isoform, a critical effector of growth factor signalling. 1938 allosterically activates PI3Kα through a unique mechanism, by enhancing multiple steps of the PI3Kα catalytic cycle, and causes both local and global conformational changes in the PI3Kα structure. This compound is selective for PI3Kα over other PI3K isoforms and multiple protein and lipid kinases. It transiently activates PI3K signalling in all rodent and human cells tested, resulting in cellular responses such as proliferation and neurite outgrowth. In rodent models, acute treatment with 1938 provides cardioprotection from ischaemia reperfusion injury and, upon local administration, enhances nerve regeneration following nerve crush. This study identifies a unique chemical tool to directly probe PI3Kα signalling and a novel approach to modulate PI3K activity, widening the therapeutic potential of targeting these enzymes, through short-term activation for tissue protection and regeneration. Our findings illustrate the potential of activating kinases for therapeutic benefit, a currently largely untapped area of drug development.

## Introduction

Compared to the development of protein and lipid kinase inhibitors, efforts to generate pharmacological activators to harness the beneficial activities of some of these enzymes, such as in tissue regeneration and protection, wound healing, immune stimulation and metabolic sensitization, have been very limited^1–5^.

Class IA PI3Ks signal downstream of tyrosine kinases, G protein-coupled receptors and small GTPases to regulate cell metabolism, growth, proliferation and migration. They consist of a p110α, β or δ catalytic subunit and a p85 regulatory subunit (further referred to as PI3Kα, PI3Kβ and PI3Kδ), with a broad tissue distribution (p110α, p110β) or enriched in leukocytes (p110δ)^6,7^. Overactivation of class IA PI3Ks and their effectors AKT and mTORC1 in cancer and the immune system has driven extensive PI3K pathway inhibitor development^6^.

PI3K pathway *activation* could also be of therapeutic benefit in tissue protection and regeneration. PI3K inhibition dampens the protective effect of growth factors and other agents in models of cell/tissue damage^8–11^. This includes protection from ischaemia-reperfusion injury (IRI) (such as in neurons following a stroke^12,13^ and in cardiomyocytes following myocardial infarction^14^), protection from ionising radiation^15^, enhancement of tissue/wound repair^8,16^ and neuro-protection/regeneration^17–20^.

Genetic strategies of PI3K pathway activation tested in this context include expression of activated alleles of PI3Kα^21^ or AKT^22^, or deletion/knockdown of PTEN, a lipid phosphatase that downregulates PI3K signalling^8,23^. Non-genetic PI3K pathway activators include p85-binding phospho-peptides^17,24^, the AKT-activating small molecule SC79^25,26^ and PTEN inhibitors^8,27^. These agents have poor drug-like properties, obscure mechanisms of PI3K pathway activation, and poor selectivity for their target proteins.

### A screen for PI3Kα activators

**Fig. 1:**
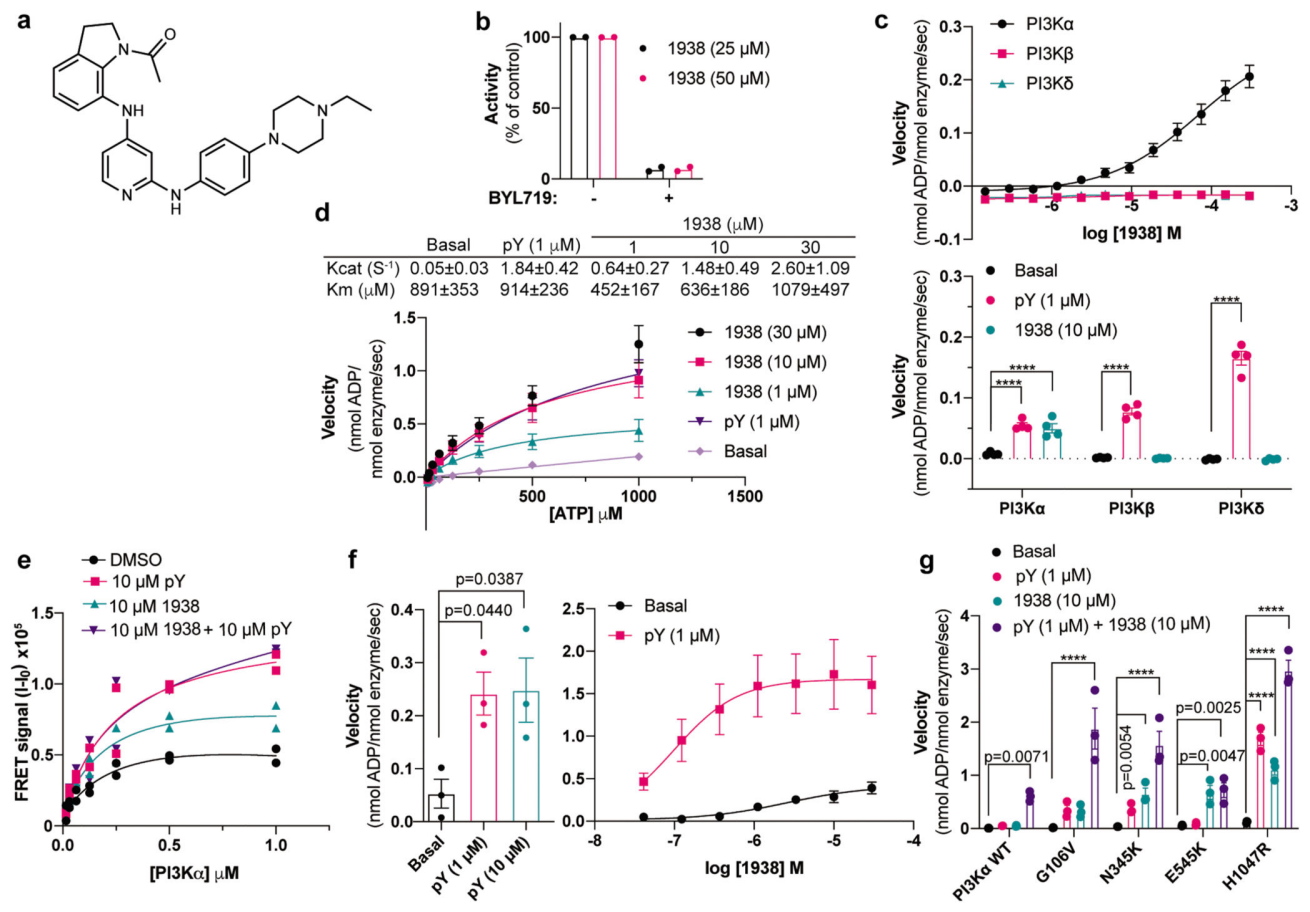
Biochemical mechanism of PI3Kα activation by 1938. **a**, Structure of UCL-TRO-1938 (referred to in the text as 1938). **b**, Effect of the PI3Kα-selective inhibitor BYL719 (500 nM) on 1938-activated PI3K. Enzyme activity in the presence of 1938 only was considered 100%. **c**, Selectivity of 1938 for PI3Kα over PI3Kβ and PI3Kδ. **d**, Enzyme kinetics (calculated using kcat function in Prism 8) upon ATP titration on PI3Kα with or without 1938 and pY. **e**, Membrane binding of PI3Kα shown as FRET signal (I-I0). I, fluorescence intensity at 520 nm, I0, fluorescence intensity at 520 nm in the absence of enzyme. **f,** Effect of 1938 on PI3Kα catalytic activity in the presence of a saturating dose of pY. **g**, Effect of 1938 on the catalytic activity of oncogenic mutants of PI3Kα. Data shown as n=2 independent experiments (**b,e**). Data shown as mean ± SEM, n=6 (**c,** top), n=4 (**c,** bottom), n=3. (**d,f,g**) experiments. Kinetic values in **d** shown as mean ± SD. Statistical analysis performed with two way ANOVA, Tukey’s multiple comparisons test (**c**) or Dunnett’s multiple comparisons test (**g**); one way ANOVA, Dunnett’s multiple comparisons test (**f**). ****P<0.0001.

We conducted an unbiased high throughput screen for small molecule activators of recombinant human p110α/p85α with liposomes mimicking the plasma membrane composition, enriched with 5% phosphatidylinositol(4,5)bisphosphate (PIP2), the natural PI3Kα substrate. Confirmed hits were screened by a fluorescence polarisation assay (an orthogonal assay for lipid kinase activity) and microscale thermophoresis (to test for direct PI3Kα binding). Validated hits were investigated for the generation of phospho-S473-AKT (pAKT^S473^) in the human A549 cell line. Subsequent medicinal chemistry to improve cellular potency (as measured by pAKT^S473^ in A549) led to the generation of UCL-TRO-1938 (Fig. 1a), further referred to as 1938.

### 1938 is an allosteric activator of PI3Kα

**Extended data Fig. 1:**
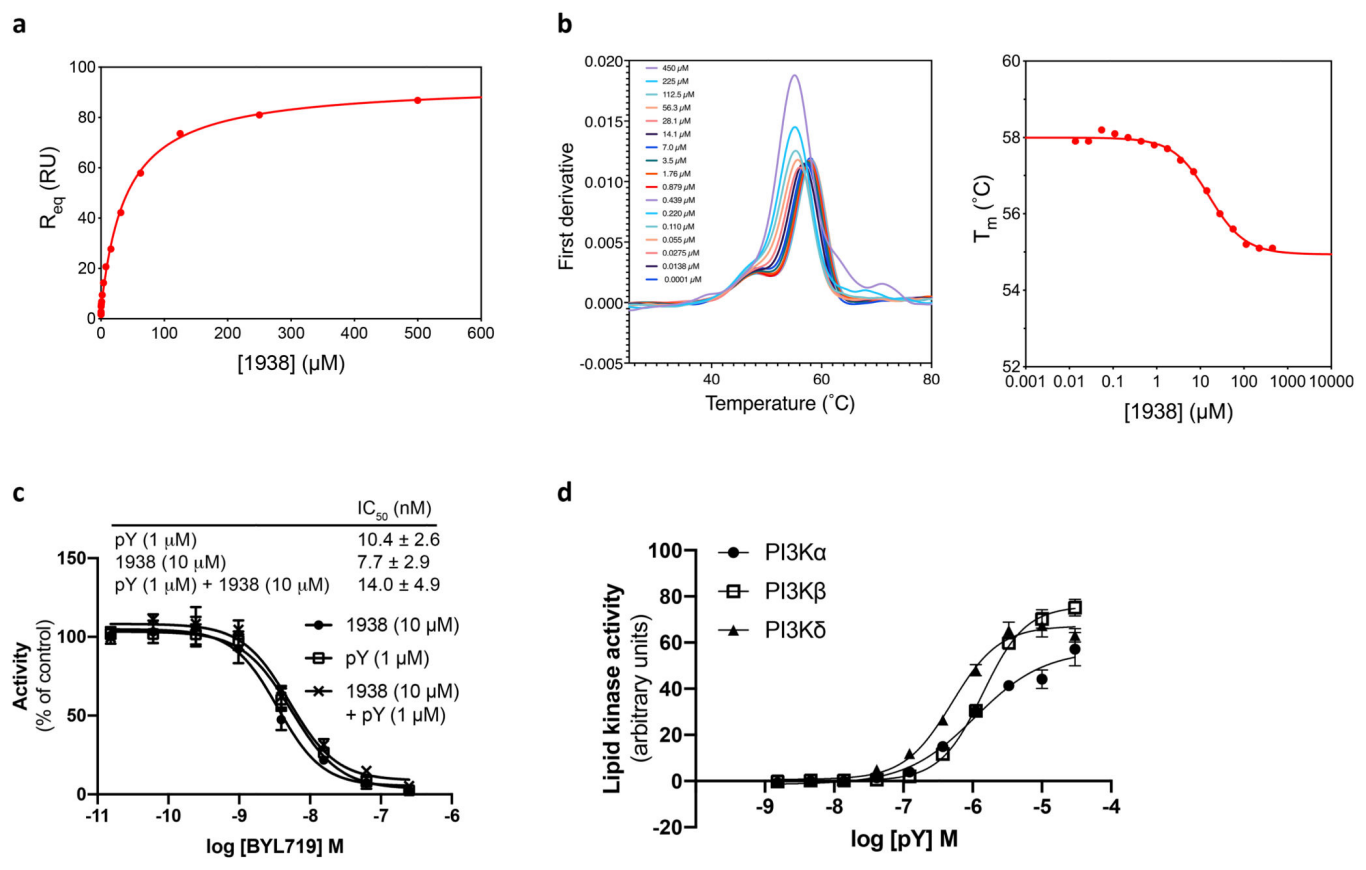
Additional biochemical data on 1938. **a**, Determination of *K*_d_ for the dissociation of 1938 from p110α/p85α by surface plasmon resonance (SPR). SPR equilibrium response titration of 1938 binding to immobilized p110α/p85α, yielding a dissociation constant *K*_d_ = 36 ± 5 μM. **b**, Determination of *K*_d_ for the dissociation of 1938 from p110α/p85α by differential scanning fluorimetry (DSF). The first derivatives of the fluorescence change of p110α/p85α upon thermal denaturation at the stated 1938 concentrations *(left panel)* were used to plot the melting temperature (Tm) *(right panel)*. Fits to data gave a *K*_d_ = 16 ± 2 μM. *K*_d_ shown as mean ± SD (n=3 independent experiments). Representative experiment is shown. **c**, Effect of 1938 on the IC50 of BYL719 for PI3Kα. Data shown as mean ± SEM (n=3 independent experiments). **d**, Activation of class IA PI3K isoforms by a concentration range of pY using the ADP-Glo assay. Data shown as mean ± SEM (n=3 independent experiments).

1938 is a drug-like compound (MW <500, cLogP <5), with an EC50 of ~60 μM for PI3Kα (assessed by *in vitro* lipid kinase activity) and a *K*_d_ for PI3Kα of 36±5 μM and 16±2 μM (determined by surface plasmon resonance and by differential scanning fluorimetry, respectively; Extended Data Fig. 1a,b). 1938-stimulated PI3Kα activity was fully inhibited by the nanomolar potency ATP-competitive PI3Kα-selective inhibitor BYL719^28^ (Fig. 1b, Extended Data Fig. 1c).

1938 activates PI3Kα but not PI3Kβ or PI3Kδ (Fig. 1c). This contrasts with activation of all class IA PI3K isoforms by pY (Extended Data Fig. 1d), a bis-phosphorylated phosphopeptide (based on a PDGF-receptor peptide phosphorylated on Tyr-740 and Tyr-751^29^) that mimicks tyrosine-phosphorylated peptides in receptors and adaptor proteins that engage p85α SH2 domains to release p85-mediated PI3K inhibition^29^.

Like pY, 1938 increased the kcat of PI3Kα (Fig. 1d). Unlike pY, which did not affect the Km of PI3Kα for ATP, 1938 slightly decreased Km at activator concentrations of 1 and 10 μM, but not at 30 μM (Fig. 1d). 1938 also induced increased PI3Kα binding to lipid membranes, to a maximum level of about half of that induced by pY (Fig. 1e).

Combination of a saturating concentration of pY with 1938 (Fig. 1f, *left*), led to synergistic PI3Kα activation (Fig. 1f, *right*), indicating that 1938 activates PI3Kα via a different mechanism or enhances activatory events beyond those induced by pY. This synergy is unlikely to involve changes in membrane binding, given that the combination of 1938 with pY did not further increase PI3Kα membrane association beyond that induced by pY (Fig. 1e).

Oncogenic mutants of p110α each activate p85α/p110α through different mechanisms^29^. 1938 activated the G106V, N345K and H1047R mutants to levels comparable with stimulation with pY. Although the E545K mutant was insensitive to pY stimulation, as previously shown^29^, it could be further activated by 1938 (Fig. 1g). Co-stimulation using 1938 with pY also led to a synergistic activation of G106V and N345K, and additive activation of H1047R (Fig. 1g).

In summary, 1938 does not specifically mimic the mechanism of activation of any single oncogenic p110α mutation tested, but instead it stimulates PI3Kα by enhancing multiple events associated with natural and mutation-mediated PI3Kα activation.

### 1938 changes the conformation of PI3Kα

Class IA PI3K activation upon binding to phosphorylated tyrosine motifs in proteins occurs through the release of inhibitory interactions between p85 and p110, by: (1) release of p85α-nSH2 and p85α-iSH2 from the p110α-helical and p110α-C2 domains, respectively; (2) movement of the N-terminal p85-binding domain in p110α relative to the rest of the catalytic subunit and (3) interaction of the p110α kinase domain with the lipid membrane^29^.

**Extended data Fig. 2:**
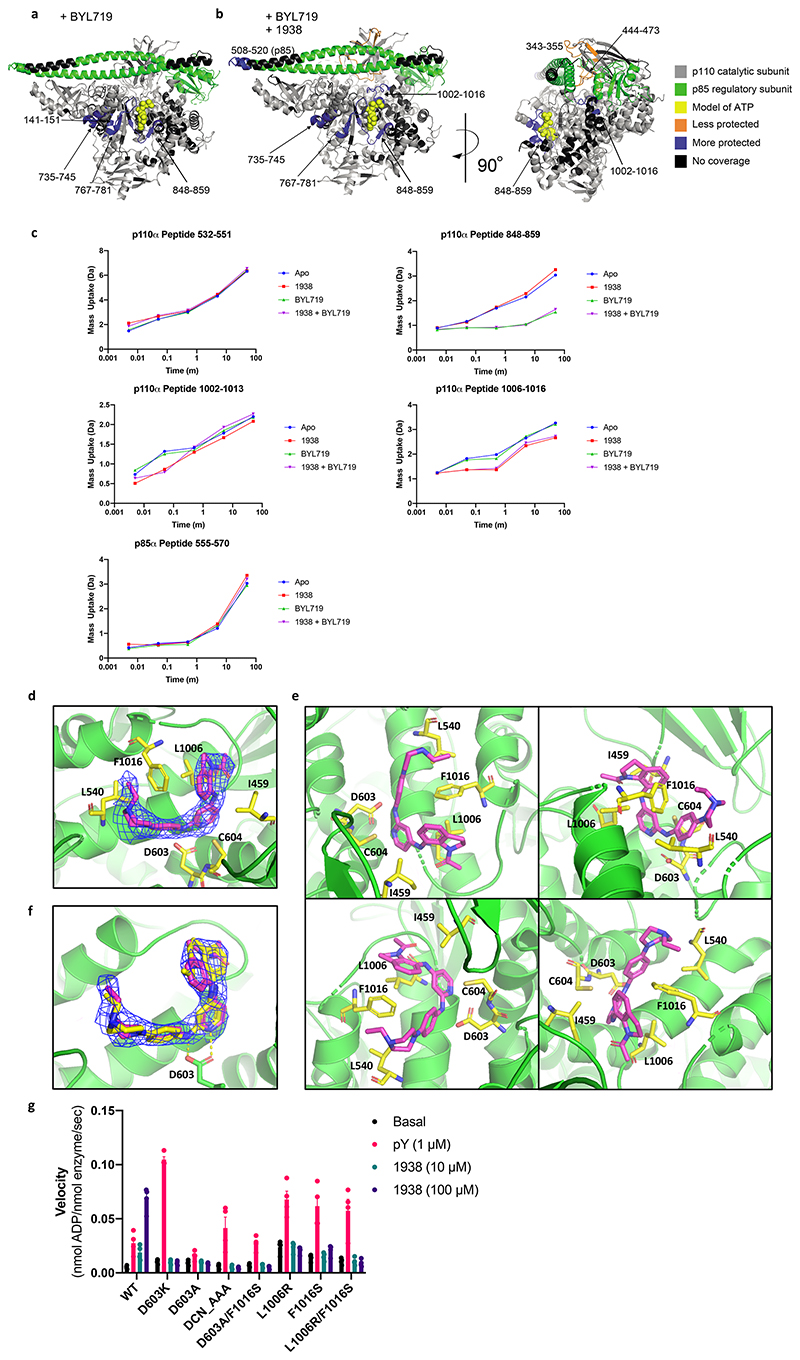
Additional data on HDX-MS and crystallography. Structural changes induced by BYL719 (**a***)*, or 1938 in combination with BYL719 (**b**), assessed by HDX-MS in full-length p110α/p85α, highlighted on the structure of p110α (gray)/niSH2-p85α (green) (PDB:4ZOP). Selection threshhold for significant peptides: a-b difference ≥2.5%, Da difference ≥0.25, p-value <0.05 (unpaired t-test). **c**, Peptide uptake from HDX-MS. A selection of peptides (peptides 848-859, 532-551, 1002-1013 and 1006-1016 are from p110α, peptide 555-570 is from p85α) exhibiting significant differences in solvent exchange rates on the addition of 1938 (red), BYL719 (green), both (purple) or neither (blue). Data presented here is from one of three biological replicates Five time points were measured in triplicate. Each point is the mean of one biological repeat. **d**, Omit map of ligand 1938 (mFo-DFc) calculated at +/- 3σ using phenix.polder. **e**, 1938 bound to p110α shown in multiple orientations. **f**, Two possible orientations shown for the 1938 ligand (magenta and yellow sticks) in the p110α crystal structure, both fit the Sigma-weighted density map (blue, 2mFo-DFc) equally well.Yellow dashes show predicted hydrogen bonds. **g**, Effect of 1938 on catalytic activity of p110α proteins with mutations in the 1938-binding pocket. Data shown as mean ± SEM (n=4 independent experiments).

**Fig. 2:**
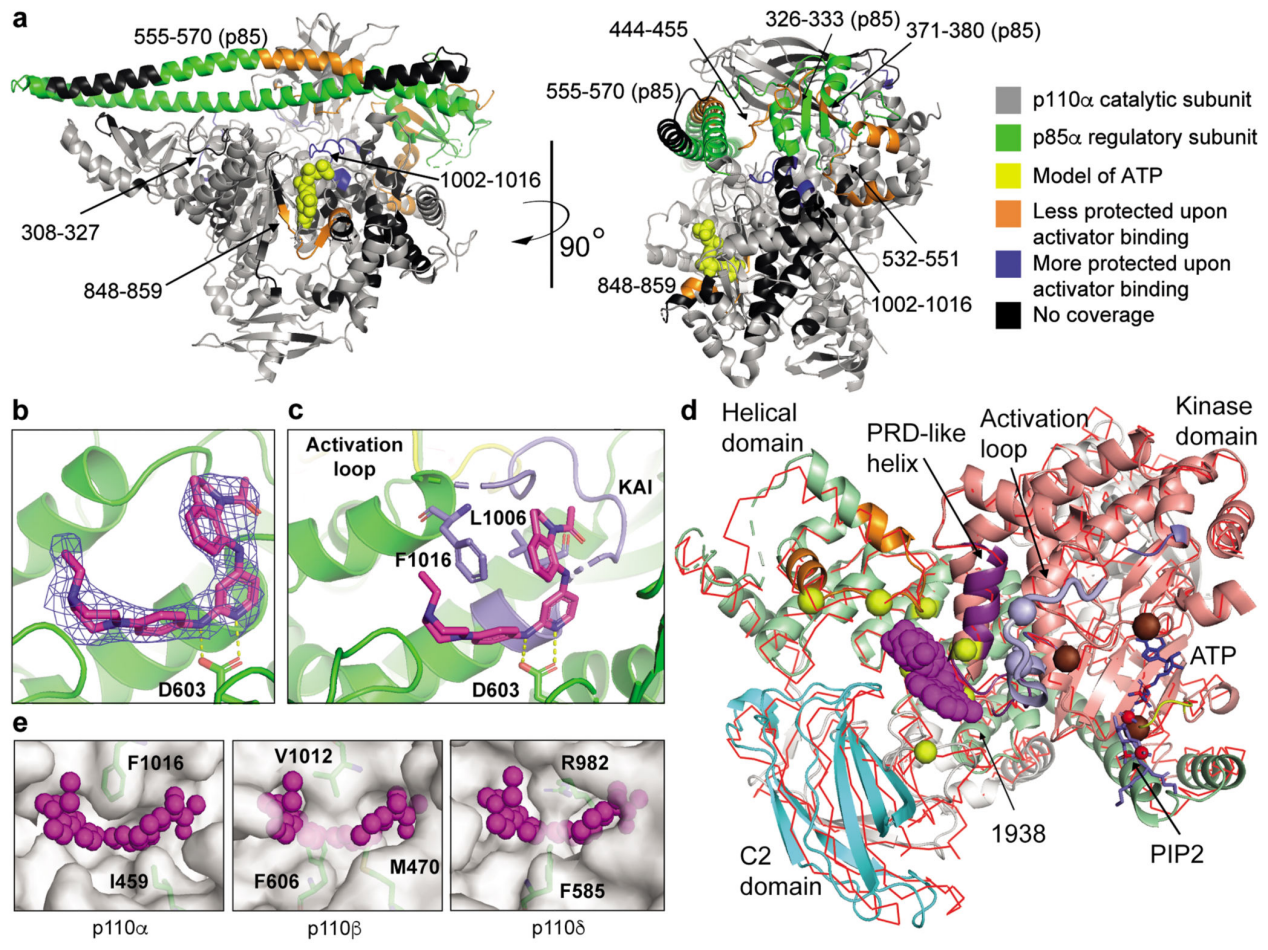
Structural mechanism of PI3Kα activation by 1938. **a**, Structural changes induced by 1938 as assessed by HDX-MS in full-length p110α/p85α, highlighted on the structure of p110α (gray)/niSH2-p85α (green) (pdb:4ZOP). Selection threshhold for significant peptides: a-b difference ≥2.5%, Da difference ≥0.25, p-value <0.05 (unpaired t-test). **b**, Sigma-weighted density map in blue (2mFo-DFc) for the 1938 ligand (magenta) in the p110α crystal structure. Yellow dashes show predicted H-bonds. **c**, Crystal structure of 1938 bound to p110α; 1938 (magenta), activation loop (yellow), loop 1002-1016 (kinase/activator interface, slate), predicted H-bonds (yellow dashes). **d**, Comparison of the 1938-bound p110α with apo-p110α. The 1938-bound structure is shown in cartoon representation, while the apo-model is shown as a superimposed red Cα trace. 1938 shown as magenta blob, PRD-like helix shown in purple. Yellow spheres mark the sites of cancer-associated mutations from the COSMIC database that are near the 1938-binding site (only mutations with >10 reports are shown). Regions showing decreased HDX-MS protection for the common helical domain mutations are colored orange. PIP_2_ substrate (slate) has been modelled in the active based on 4OVV. A region of the activation loop (thick worm representation, slate) has been taken from 7PG5 since it is disordered in the 1938-bound structure. Slate spheres represent residues important for PIP_2_ recognition (K942 and R949). Chocolate spheres represent residues essential for phosphate transfer (K776, H917 and H936). A bound ATP (blue) has been modelled based on PDB ID 1E8X. The ATP binding loop is coloured yellow. Phosphates in PIP_2_ and ATP are shown in red. **e**, Comparison of 1938-binding pocket in p110α with homologous regions in p110β and p110δ.

HDX-MS of PI3Kα incubated with 1938 revealed changes in protection that occurred mostly outside the ATP-binding site (Fig. 2a; Extended Data Fig. 2a-c; Supplementary Table 1). There was protection of a region in the C-terminus of the kinase domain consisting of amino acids (AA) 1002-1016 of p110α, suggesting that this region might be the 1938 binding site on p110α (further referred to as the kinase/activator interface).

Increases in solvent exchange rate in several regions were also observed: the p85α-nSH2 domain (AA326-333 and 371-380), the p85α-iSH2 domain (AA555-570) and multiple regions in p110α, namely (from N- to C-terminus): AA444-455 (interface between p85α-iSH2 and p110α-C2 domains), AA532-551 (interface between p85α-nSH2 and p110α-helical domains), and AA848-859 (ATP-binding site). These changes are compatible with the notion that 1938 activates PI3Kα by disrupting inhibitory contacts at the p85α-nSH2/p110α-helical and p85α-iSH2/p110α-C2 interfaces, leading to decreased inhibition of p85α on p110α.

HDX-MS with BYL719 produced a characteristic ATP-competitive footprint on PI3Kα, with strong protections of AA848-859 in the hinge between the N- and C-lobes of the kinase domain and the AA735-745 and AA767-781 regions adjacent to the ATP binding site (Extended Data Fig. 2a).

Combination of BYL719 and 1938 yielded a combined footprint that largely overlapped with that of each ligand separately bound to PI3Kα, with the protections in the kinase hinge and the AA1002-1016 regions, along with exposures in the p110α-C2 interface (Extended Data Fig. 2b), suggesting that PI3Kα can accomodate both ligands simultaneously.

We next attempted to crystallize full-length p110α/niSH2-p85α in the presence of 1938. Despite obtaining PI3Kα crystals, no 1938 was visible, either upon co-crystallisation or upon compound soaking into preformed crystals (PDB:7PG5). Co-crystallisation of PI3Kα with 1938 and BYL719 resulted in crystals in which only density for BYL719 was visible (PDB:7PG6; Supplementary Table 2a).

We next used a deletion variant of p110α (p110α AA105-1048)^30^ in which the p85 adaptor-binding domain (AA1-104) and a C-terminal membrane binding motif in the kinase domain (AA1049-1068) were deleted. This construct lacks catalytic activity but, in contrast to wild-type p110α, is stable in the absence of p85^30^. Co-crystallisation with 1938 did not yield crystals, however, soaking preformed crystals with 1938 revealed density for 1938 (Fig. 2b-d; Extended Data Fig.2d). Crystals diffracted to 2.4 Å for apo (PDB:8BFU), and 2.6 Å for 1938-bound p110α (PDB:8OW2; Supplementary Table 2b). The bound 1938 is in a pocket surrounded by residues E365, I459, L540, D603, C604, N605, Y641 of p110α and, in agreement with HDX-MS protection, S1003, L1006, G1007 and F1016 (Fig. 2c; Extended Data Fig. 2e). The core pyridine nitrogen in 1938 is predicted to be sufficiently basic to be predominantly protonated at physiological pH (Marvin Sketch 21.14, pKa calculator plug-in, Chemaxon Ltd, Váci út 133. 1138 Budapest, Hungary), and this NH^+^ makes key interactions with the side chain of D603. The acetylated indoline of 1938 sits in a pocket comprised of L1006, F1016 and I459, and makes face-to-edge interactions with F1016. Two orientations for the indoline group of 1938 fit the density equally well (yellow and magenta sticks in Extended Data Fig. 2f), and both orientations have an internal H-bond between the oxygen of the acetylated indoline and the NH group connecting the indoline to the pyridine. However, in one orientation (magenta carbons) the compound is less strained, with a better internal H-bond geometry, so this is our preferred model (Fig. 2b).Binding of 1938 induces F1016 to move away from the pocket in order to accommodate the ligand. The piperazine is surrounded by E365 and L540, and points out towards solvent.

1938 induced global conformational shifts in the p110α crystal structure (Fig. 2d; Video S1), with the C2 and helical domains moving away from the kinase domain, the AA1002-1016 kinase/activator interface moving away from the helical domain and the α-helix AA1016-1026 moving toward the active site (Fig. 2d; Video S1). This 1016-1026 helix is structurally analogous to a region in the PI3K-like protein kinases (PIKKs) known as the PIKK regulatory domain (PRD). In PIKKs, this region can block substrate interaction with the activation loop. In PI3Kα, the PRD-analogous region interacts with the substrate-binding activation loop (AA933-957). The AA940-954 activation loop region is disordered in both the apo and 1938-bound structures, however, in the structure of PIP2 bound to PI3Kα (PDB:4OVV^31^) and in our own apo p110α/niSH2 structure (PDB:7PG5), the activation loop is fully ordered and packed against the PRD-analogous helix. Activation loop residues K942 and R949 are important for recognizing PIP_2_^32^, suggesting that a component of the mechanism of action of 1938 is to cause repositioning of the activation loop to facilitate productive phosphotransfer to PIP_2_. In addition, 1938 binding causes pivoting of the helical domain to bring the ATP-binding loop and the N-lobe of the kinase domain towards the ATP-binding pocket (Fig. 2d; Video S1). This helical domain pivoting to close the ATP pocket is analogous to a closing of the N-lobe relative to the C-lobe that accompanies RHEB-mediated activation of mTORC1 caused by conformational changes of the FAT domain^33^. The COSMIC database^34^ shows that several cancer-associated mutations in *PIK3CA* occur at sites adjacent to the activator-binding pocket (Fig. 2d; Video S1), including two of the most common cancer-associated mutants E542K and E545K that are known to relieve inhibition of p110α by the p85α-nSH2 domain^29^. Therefore, it is possible that 1938 weakens the inhibitory effects of p85α on p110α, contributing to enzyme activation. Key components of the compound binding mode are confirmed by preliminary structure-activity relationship (SAR) analysis of our small molecule scaffold (Supplementary Table 3).

Based on the crystal structure and SAR data, we performed mutagenesis to generate 1938-resistant p110α-mutants, as follows: D603K, D603A, 603DCN_AAA605 triple-mutant, D603A/F1016S double-mutant, L1006R, F1016S, and the L1006R/F1016S double-mutant. All mutants were resistant to activation by 1938 but could be activated by pY (Extended Data Fig. 2g).

Comparison of the p110α/1938 crystal structure with that of p110β (PDB:2Y3A) and p110δ (PDB:6PYU) indicates that these PI3Ks cannot accommodate 1938 at the homologous site due to side-chains that occlude the analogous volume in which 1938 binds to p110α (Fig. 2e), explaining the high selectivity of 1938 for p110α.

### 1938 induces PI3Kα signalling in cells

PI3Kα phosphorylates PIP_2_ in the plasma membrane to PtdIns(3,4,5)P_3_ (or PIP_3_), which can be converted by 5-phosphatases to PtdIns(3,4)P_2_.

**Extended Data Fig. 3:**
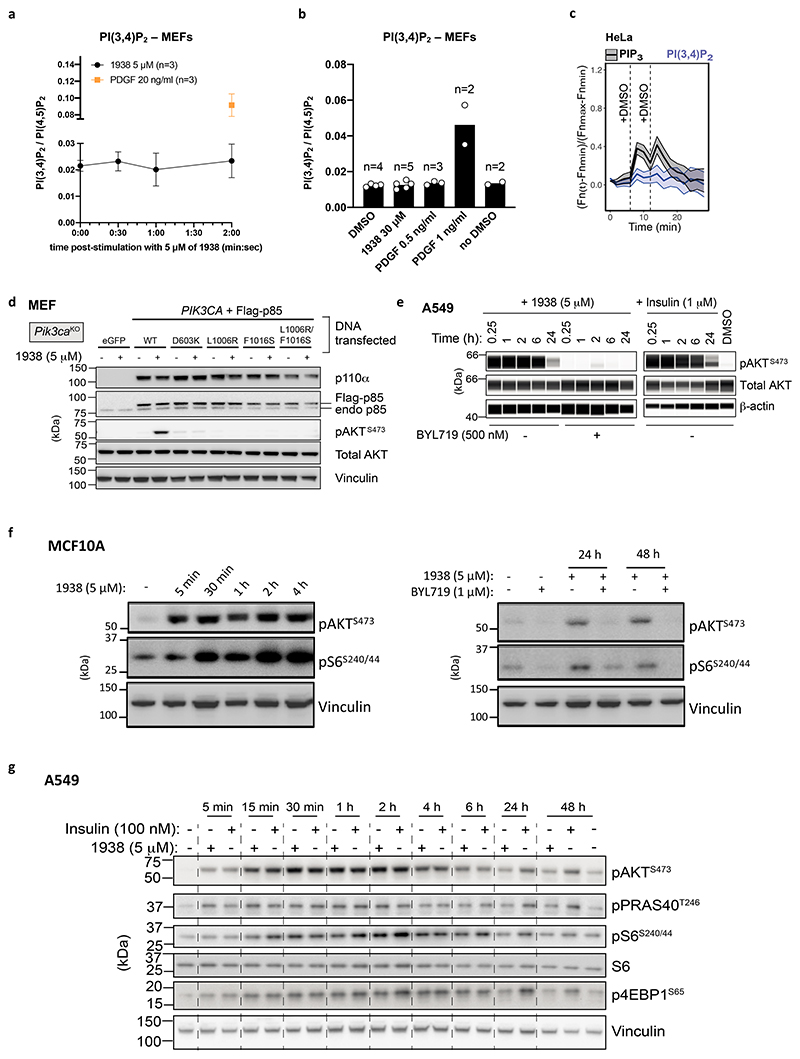
Additional data on 1938-driven signalling. **a**, MEFs were stimulated at different time points with 1938 (5 μM) or for 2 min with PDGF (20 ng/ml), followed by lipid extraction and PI(3,4)P_2_ measurement by mass spectrometry. **b**, MEFs were stimulated for 2 min with 1938 (30 μM) or PDGF (0.5 or 1 ng/ml), followed by lipid extraction and PI(3,4)P_2_ measurement by mass spectrometry. (**a,b**) n=independent experiments, shown in figure. Error bars represent SD. **c**, Control TIRF microscopy data from DMSO-treated HeLa cells expressing the PIP_3_ or the PI(3,4)P_2_ reporter. HeLa cells expressing the EGFP-tagged PIP_3_ reporter PH-ARNO-I303Ex2 (ARNO) *(black lines)* or the PI(3,4)P_2_ reporter mCherry-cPH-TAPP1x3 *(blue lines)* were stimulated with DMSO as indicated. Overlay plots (mean ± SEM) were generated by scaling to minimum and maximum values of the normalised fluorescence intensity for each time point (Fn(t)). PIP_3_ reporter data are representative of 2 experiments and 16 single cells. PI(3,4)P_2_ reporter data are representative of 4 experiments and 28 single cells. Individual measurements were acquired every 2 min. **d**, pAKT^S473^ induction by 1938 in PI3Kα-KO MEFs transiently transfected with p110α-WT or p110α-mutants. Blot representative of n=2 experiments. **e**, Time course analysis of 1938-induced pAKT^S473^ in A549 by 1938+BYL719 or a saturating insulin concentration. Blot representative of n=3 experiments. **f**, Time course analysis of 1938-induced pAKT^S473^ and pS6^S240/244^ in MCF10A cells in the presence or absence of BYL719. Shown is a representative blot of n=2 independent experiments. **g**, Time course analysis of insulin- or 1938-induced PI3K/AKT/mTORC1 signalling in A549, n=2 experiments.

**Fig. 3:**
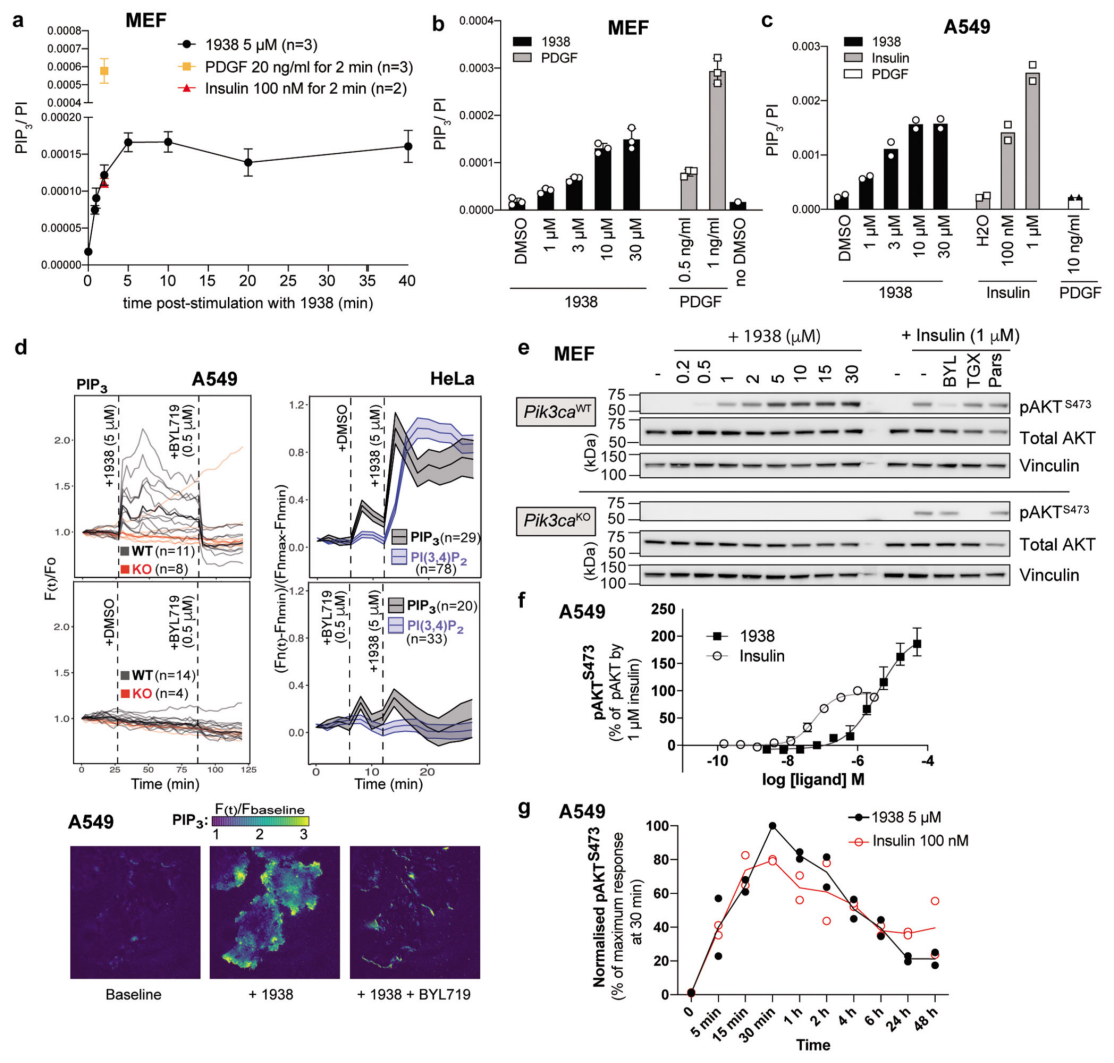
1938 activates PI3Kα signalling in cells. All cells were serum-starved overnight. **a**, Time-dependent PIP_3_ generation in MEFs stimulated with 1938, PDGF or insulin. Data shown as mean±SD, n=number of experiments. **b**, Dose-dependent PIP_3_ generation by 2 min stimulation with the indicated agonists in MEFs (mean±SD, n=3 experiments except no DMSO (n=1)) and **c**, A549 cells (n=2 experiments). **d**, Total internal fluorescence (TIRF) microscopy of 3-phosphoinositide reporter-expressing A549 or HeLa cells treated with DMSO, 1938 and BYL719. Thick lines specify medians; n=number of single cells. A549: PIP_3_ reporter-expressing PI3Kα-WT or PI3Kα-KO cells, with data from one experiment. HeLa: PIP_3_- or PI(3,4)P_2_-reporter cells, with PIP_3_ and PI(3,4)P_2_ data representative of 2 and 4 experiments, respectively. Shown below is a representative TIRF image of a PI3Kα-WT A549 cell, imaged 3 min before 1938 addition; 3 min after 1938 addition at t=27 min, and 3 min after BYL719 addition at t=87 min. Scale bar: 11 μm. **e**, pAKT^S473^ induction by 15 min treatment with different doses of 1938 in PI3Kα-WT and PI3Kα-KO MEFs. BYL719 (BYL), TGX-221 (TGX) and Parsaclisib (Pars) were used at 0.5 μM, 0.2 μM and 0.05 μM, respectively. Blot representative of n=3 experiments. **f**, pAKT^S473^ induction (measured by ELISA) in A549 by a 1938 dose titration or insulin. Data shown as mean ± SEM (n=3 experiments). **g**, Time course analysis of insulin- or 1938-induced PI3K/AKT/mTORC1 signalling in A549, n=2 experiments. Quantification of pAKT^S473^/vinculin signal ratio, expressed relative to treatment with DMSO only.

In MEFs, 1938 increased PIP_3_ levels within 30 sec, as assessed by mass spectrometry (MS)^35^, maxing at 5 min and maintained at this maximum level for up to 40 min (Fig. 3a). At the 2 min time point, the PIP_3_ levels induced by 1938 were comparable to those induced by insulin, but lower than those induced by PDGF. The observation of different PIP_3_ levels induced by insulin and PDGF is in line with the notion that PI3Kα is the sole mediator of PIP_3_ production downstream of insulin^36,37^, whereas PDGF activates both PI3Kα and PI3Kβ, with PI3Kβ contributing substantially to PDGF-stimulated PIP_3_-generation in MEFs^38^. In the same experiment as in Fig. 3a, a PI(3,4)P_2_ signal was detected in MEFs upon PDGF stimulation but not with 1938 (at 5 μM; Extended Data Fig. 3a). This is consistent with a higher threshold of PI(3,4)P_2_ detection compared to PIP_3_ by MS (due primarily to background contamination^39^), together with the lower PI3K activation by 1938 compared to a high dose of PDGF, as is also illustrated by the experiments below.

When tested at different doses at a fixed 2 min time point, PIP_3_ induction by 1938 in MEFs had an EC50 of ~5 μM, plateauing around 10 μM, at a substantially lower level of PIP_3_ to that induced by PDGF at 1 or 3 ng/ml (Fig. 3b). These maximal 1938-induced PIP_3_ levels are below those required to give rise to sufficient PI(3,4)P_2_ to be detectable by MS, a conclusion also supported by the observation that substantial levels of PIP_3_ induced by lower doses of PDGF (e.g. 0.5 ng/ml) also did not give rise to PI(3,4)P_2_ levels detectable by MS (Extended Data Fig. 3b). Similar to what was observed for MEFs, stimulation of A549 cells for 2 min with a dose range of 1938 revealed that the PIP_3_ response to 1938 maxed out at 10 μM (Fig. 3c). A strong PIP_3_ response was also observed with insulin in A549, with no PIP_3_ induced by PDGF, in line with the absence of the PDGF receptor in epithelial cells, including in A549^40^.

Live imaging of A549 cells expressing a fluorescent biosensor for PIP_3_^41^ showed acute plasma membrane-associated PIP_3_ production upon 1938 addition, which could be fully and acutely neutralized by BYL719 (Fig. 3d; Video S2,S3). This PIP_3_ signal was not seen in PI3Kα-KO A549 (Fig. 3d*;* Video S4). In HeLa cells, 1938 also induced an acute and BYL719-sensitive burst of PIP_3_ (Fig. 3d), followed by the generation of membrane-associated PI(3,4)P_2_ (Fig. 3d), with a timing in line with the known mechanism of PIP_3_ conversion to PI(3,4)P_2_ by 5-phosphatases^42–44^ and similar kinetics of PIP_3_/PI(3,4)P_2_ production in insulin-stimulated HeLa cells^41^. The small increases in signal upon addition of agonists (Fig. 3d) represent a non-specific response to medium addition in HeLa cells (Extended Data Fig. 3c).

Treatment with 1938 for 15 min increased pAKT^S473^ levels in a concentration-dependent manner in PI3Kα-WT MEFs, with an EC50 of ~2-4 μM (Fig. 3e), with no pAKT^S473^ signal in 1938-stimulated PI3Kα-KO MEFs (Fig. 3e). The latter cells still respond to insulin, but in a PI3Kβ-dependent manner, as shown by sensitivity of insulin-stimulated pAKT^S473^ to the PI3Kβ-selective inhibitor TGX-221^45^ (Fig. 3e). Expression in PI3Kα-KO MEFs of WT PI3Kα restored 1938-mediated pAKT^S473^ stimulation (Extended Data Fig. 3d), while none of the p110α-mutants resistant to 1938-activation in *in vitro* kinase assays showed a response to 1938, as assessed by pAKT^S473^ induction (Extended Data Fig. 3d). 1938 treatment of A549 and MCF10A also led to a BYL719-sensitive increase in pAKT^S473^ (Extended Data Fig. 3e,f).

A dose titration of 15 min stimulation with 1938 and insulin in A549 cells revealed that in these cells, 1938 can activate the PI3K pathway as measured by pAKT^S473^ generation, beyond the level of pathway activation by saturating doses of insulin, namely ~200% of Emax of 1 μM insulin at doses of 5-10 μM 1938 (Fig. 3f). The induction of pAKT^S473^ in A549 and MCF10A by 1938 (5 μM) was rapid (5 min; Fig. 3g; Extended Data Fig. 3f,g), reaching peak activation at 30 min and persisting for a few hours before returning to levels slightly above baseline 24h or 48h later (Fig. 3g; Extended Data Fig. 3e). Similar observations were made for mTORC1 pathway activation, as measured by phosphorylation of S6^S240/244^ and 4EBP1^S65^ (Extended Data Fig. 3g). Interestingly, the kinetics of Akt/mTORC1 pathway activation was overall similar to that induced by insulin (Fig. 3g; Extended Data Fig. 3e,g), suggesting that 1938-mediated PI3K pathway activation is subject to the endogenous cellular feedback mechanisms within the PI3K signalling pathway^46^.

In summary, 1938 activates both proximal and distal signalling in a dose- and PI3Kα-dependent manner in rodent and human cells, demonstrating its ability to directly activate PI3Kα signalling in cells.

### Unbiased assessment of 1938 signalling

**Extended data Fig. 4:**
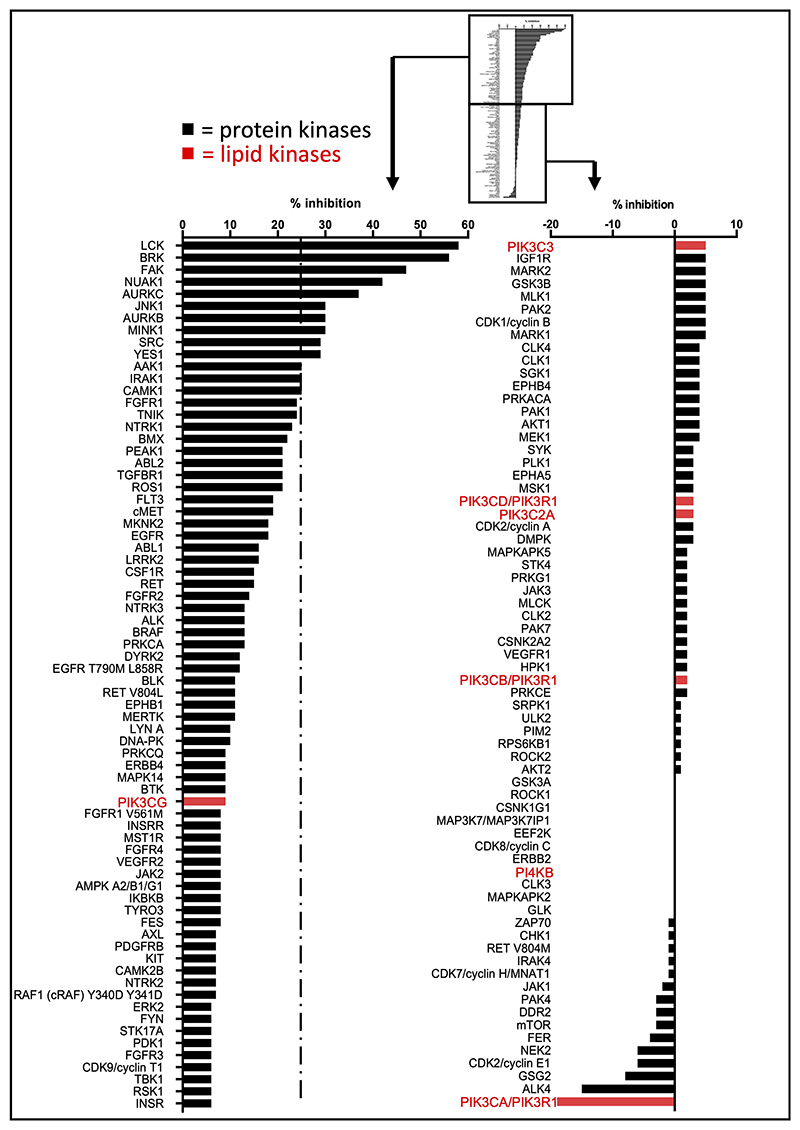
*In vitro* selectivity profile of 1938 (1 μM) on 133 protein kinases and 7 lipid kinases visualised as a waterfall plot. In the waterfall plot, the protein and lipid kinases are labeled in black and red, respectively, with the dashed line delineating 25% of kinase inhibition.

**Extended data Fig. 5:**
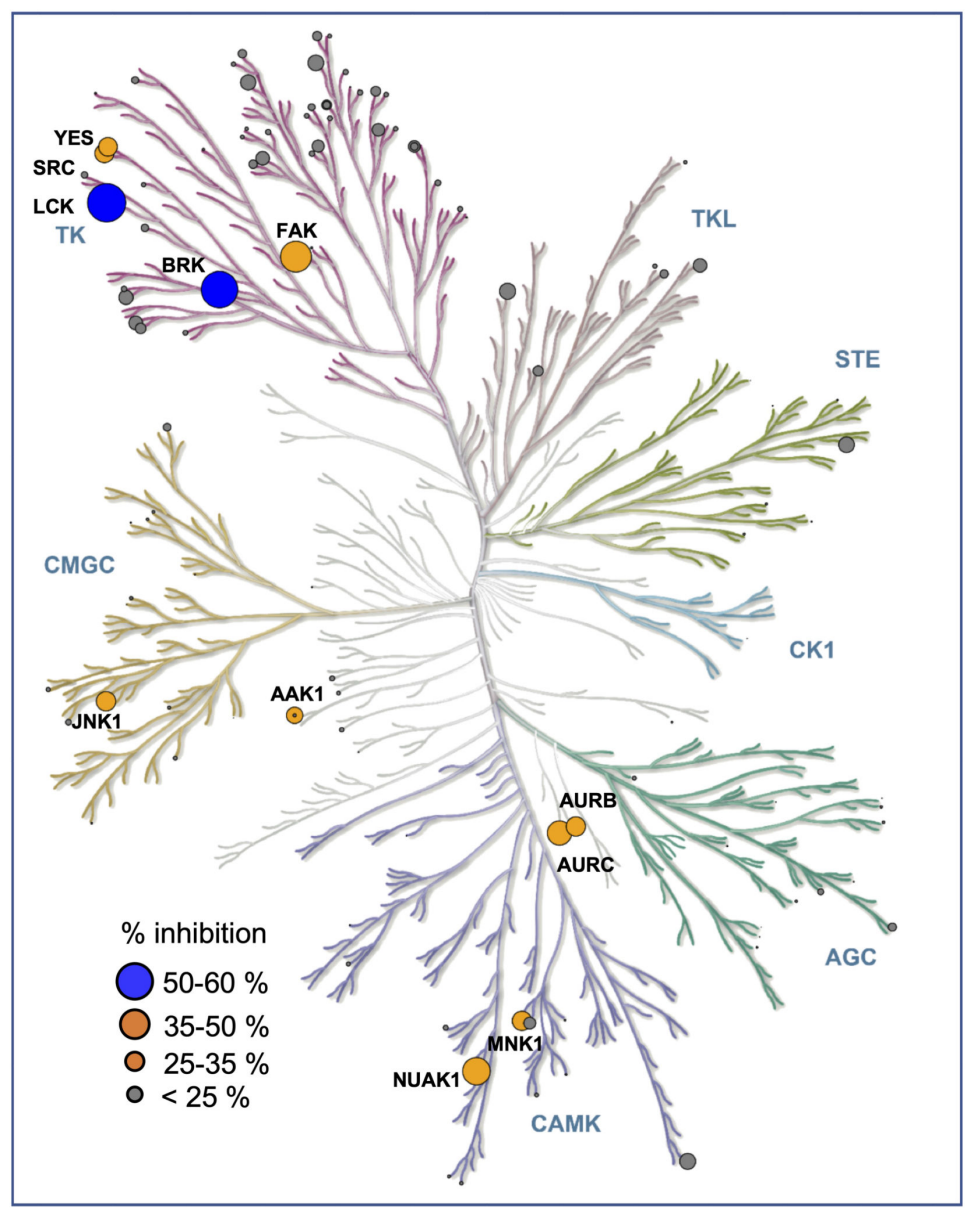
*In vitro* selectivity profile of 1938 (1 μM) on 133 protein kinases and 7 lipid kinases. Visualised as a kinome tree using KinMap.

1938 contains a pyridine core, a scaffold of multiple kinase inhibitors. A key feature of many kinase inhibitors is their ability to form mono-, bi- or tridentate H-bonding with the hinge region between the N- and C-lobes of kinase domains. The key interaction usually involves the inhibitor accepting a H-bond from the backbone amide in the hinge in the ATP-binding site. As mentioned above, the core pyridine nitrogen in 1938 is predicted to be sufficiently basic and predominantly protonated at physiological pH, which is likely to render this NH^+^ unable to form the donor-acceptor motif characteristic of standard kinase inhibitors. In order to gain insight into possible kinase inhibitory effects of 1938, we tested its impact in a panel of 133 protein kinases and 7 lipid kinases (Supplementary Tables 4-6; Extended Data Fig. 4, 5). At 1 μM of 1938, 13 protein kinases were inhibited between 25-50%, with the LCK and BRK protein kinases inhibited by 58% and 56%, respectively. It is important to note that the *in vitro* kinase assays with LCK and BRK were performed in the presence of 50 and 75 μM ATP, respectively. If 1938 were to act as an ATP-competitive inhibitor for these kinases, the inhibition by 1938 in cells is expected to be significantly lower, given that the ATP concentration in cells is 1-10 mM, i.e. ≥200x higher than tested in the kinase counterscreen. Overall, these data indicate that in cells, 1938 is unlikely to inhibit any of the kinases in the panel tested.

**Extended Data Fig. 6:**
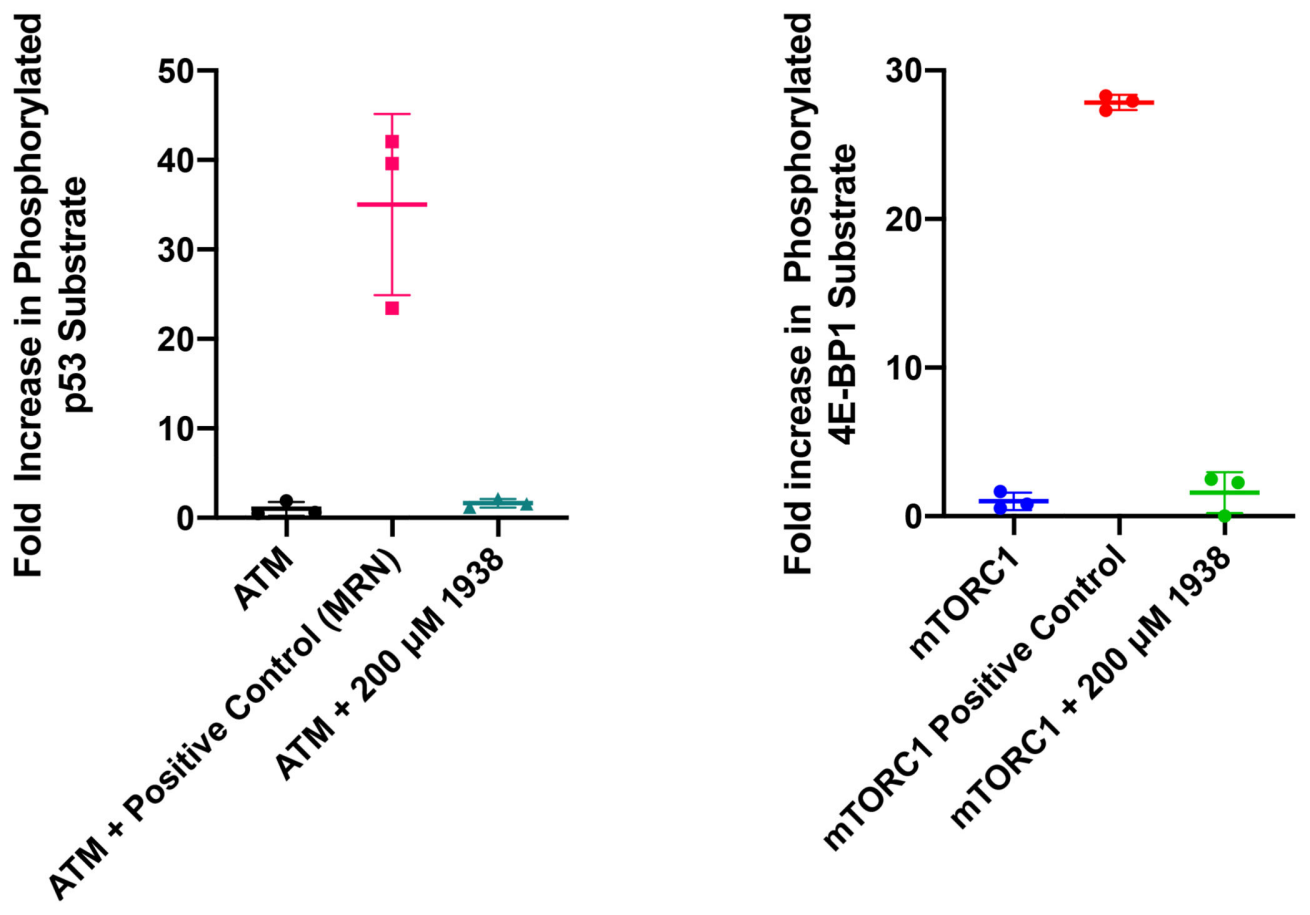
Effect of 1938 on *in vitro* kinase activity of the PI3K-related kinases ATM and mTORC1 (mTOR/RAPTOR/LST8 complex). The kinases were incubated at 30°C for 30 min (ATM) or 3 h (mTORC1), with or without 200 μM 1938 in the presence of their respective substrates (GST-p53 for ATM and 4E-BP1 for mTORC1), followed by analysis and quantification as described in Methods. The positive control for ATM was inclusion of the MRN complex (Mre11-Rad50-Nbs1), known to activate ATM, in the kinase reaction. The positive control for mTORC1 was the use of a triple amount of mTORC1 complex in the kinase reaction. Data show individual experiments (n=3), error bars represent mean ± SD.

1938 did not affect the activity of the other PI3K isoforms in the panel (PI3Kβ, PI3Kγ, PI3Kδ, PI3K-C2α and Vps34) or the PI3K-related kinases PI4Kβ, mTOR and DNA-PK (Extended Data Fig. 4, 5; Supplementary Table 4). In separate *in vitro* assays, 1938 did not affect the activity of the PI3K-related kinases ATM (Extended Data Fig. 6, *left*) and mTORC1 [Extended Data Fig. 6, *right;* tested as the mTOR/RAPTOR/LST8 complex; note that mTOR activity in the ThermoFisher screen (Extended Data Fig. 4, 5) was tested on baculovirus-expressed human mTOR/FRAP1 (AA1360-2549).

**Extended Data Fig. 7:**
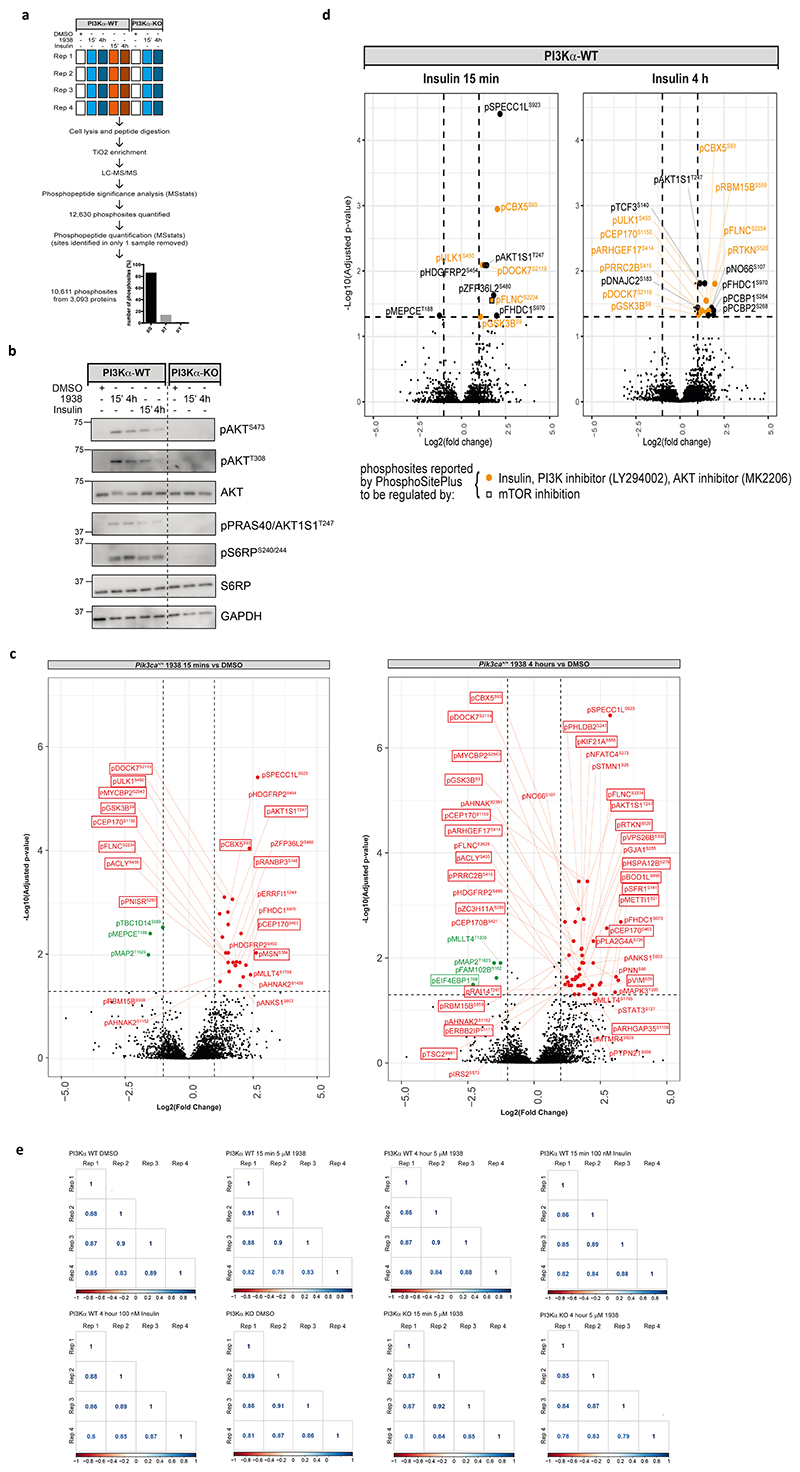
Phosphoproteomics experimental set-up and control data. **a**, Experimental design and workflow of phosphoproteomics experiment. PI3Kα-WT and PI3Kα-KO MEFs were serum-starved overnight, stimulated with DMSO, 1938 (5 μM) or insulin (100 nM) for 15 min or 4 h and processed for phosphoproteomics analysis. 10,611 phosphosites fom 3,093 proteins were analysed by MSstats, the majority of which were pSer and pThr residues. **b**, Validation of phosphoproteomics conditions. PI3Kα-WT and PI3Kα-KO MEFs were serum-starved overnight and stimulated with DMSO, 1938 (5 μM) or insulin (100 nM) for 15 min or 4 h as indicated. Lysates were immunoblotted with antibodies to pAKT^S473^, pAKT^T308^, total AKT, pPRAS40/AKT1S1^T247^, pS6RP^S240/244^, S6RP or GAPDH. Samples were from a representative phosphoproteomics experiment. Representative of n=2 independent experiments. **c**, Volcano plot of phosphosites differentially regulated by 1938 (5 μM) relative to DMSO in PI3Kα-WT MEFs. Note, these data are reproduced, enlarged and labelled from Fig. 4b. Red, upregulated phosphosites, Green, downregulated phospho-sites. Boxed phosphosites have been previously reported to be regulated by PI3K signalling (PhosphoSitePlus). **d**, Insulin stimulation induces phosphorylation of expected PI3K targets in PI3Kα-WT MEFs. Volcano plot of Log2(fold change) *versus* -log10(adjusted p-value) for phosphosites differentially regulated by (right) 15 min or (left) 4 h 100 nM insulin treatment in PI3Kα-WT MEFs relative to DMSO-treated cells. **e**, High experimental reproducibility of phosphoproteomics experiment. Quantified phosphopeptides were analysed within the model-based statistical framework MSstats. Data were log2 transformed, quantile normalised, and a linear mixed-effects model was fitted to the data. The group comparison function was employed to test for differential abundance between conditions. p-values were adjusted to control the FDR using the Benjamini-Hochberg procedure. Multi-scatter plot of the Log2(intensity) of signals obtained from each replicate against the Log2(intensity) of the same sample from all other replicates. Numbers indicate the Pearson correlation coefficient for each pair.

**Fig. 4:**
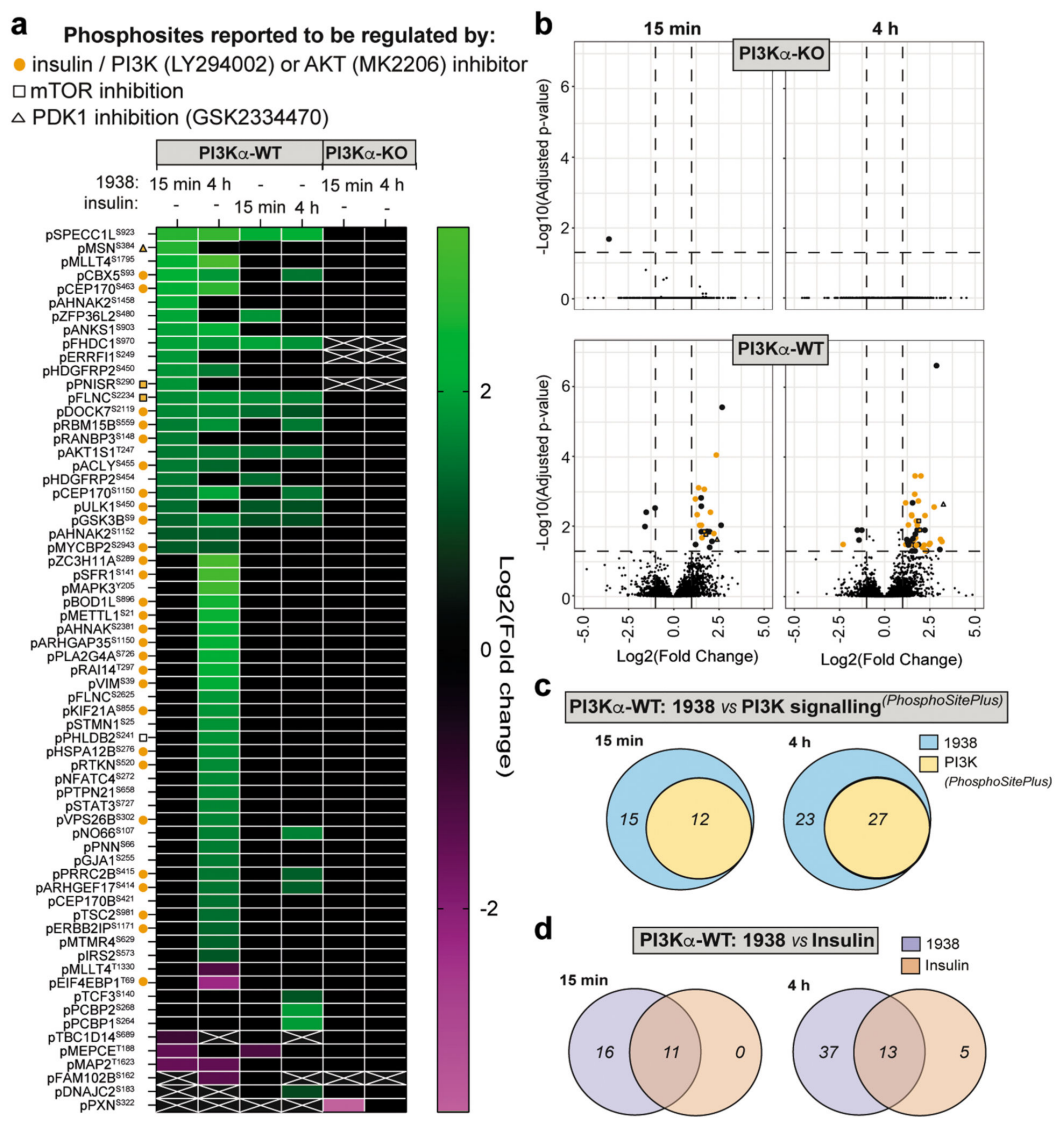
Phosphoproteomic analysis of PI3Kα-WT and PI3Kα-KO MEFs stimulated with 1938 (5 μM) or insulin (100 nM) for 15 min or 4h (n=4 independent experiments). **a**, Heat map: phosphosites significantly altered by stimulation relative to DMSO treatment. Green boxes, significantly upregulated phosphosites; magenta boxes, significantly downregulated phosphosites; white crosses: phosphosites not detected in a comparison. **b**, Volcano plot of phosphosites differentially regulated by 1938 (5 μM) in PI3Kα-WT or PI3Kα-KO MEFs, relative to DMSO-treated cells of the same genotype. Note that the PI3Kα-WT volcano plots have been reproduced in enlarged format with labeling of individual proteins and phosphosities in Extended Data Fig. 7c. **c**, Venn diagram showing overlap of the number of phosphosites significantly regulated by 1938 in PI3Kα-WT MEFs with sites that have been identified previously and are annotated in PhosphoSitePlus^47^ as regulated by insulin, IGF-1, LY294002 (pan-PI3K inhibitor) or MK2206 (AKT inhibitor). **d**, Venn diagrams showing the overlapping number of phosphosites regulated by 1938 and insulin in PI3Kα-WT MEFs.

We next investigated the impact of 1938 on cell signalling using phosphoproteomics. Serum-starved PI3Kα-WT and PI3Kα-KO MEFs were treated for 15 min or 4h with 1938 or insulin (Extended Data Fig. 7a,b), with phosphosites exhibiting >2-fold up- or downregulation relative to DMSO and adjusted p-value <0.05 defined as significantly regulated. We quantified 10,611 phosphosites from 3,093 proteins (Supplementary Table 7) of which 9100, 1420 and 91 were pSer, pThr and pTyr residues, respectively (Extended Data Fig. 7a). In line with the data shown in Fig. 3e, 1938 had little signalling impact in PI3Kα-KO MEFs (Fig. 4a,b; Extended Data Fig. 7b), with Paxillin (pPXN^S322^) being the only phosphosite altered (downregulated upon 15 min treatment but not affected by 4h stimulation; Fig. 4a).

In PI3Kα-WT MEFs, 1938 induced differential phosphorylation of 27 and 50 peptides at 15 min and 4h, respectively (Fig. 4a,b*;* Extended Data Fig. 7c; Supplementary Table 7). Most of these were upregulated and included the PI3K pathway components pAKT1S1^T247^ (PRAS40) and pGSK3β^S9^ (Fig. 4a*;* Extended Data Fig. 7c; Supplementary Table 8). Approximately half of the 1938-controlled phosphosites have been reported in PhosphoSitePlus^47^ to be regulated by insulin, IGF-1, PI3K or AKT, with some linked to regulation by mTOR or PDK1 (Fig. 4a-c; Supplementary Table 8), indicating that 1938 activates the canonical PI3K pathway. Notably, some phosphosites upregulated by 1938 in PI3Kα-WT MEFs, including top hits such as pSPECC1L^S923^, pMSN^S384^ and pMAPK3^Y205^, have not been previously linked to PI3K signalling as per PhosphoSitePlus^47^ (Fig. 4a; Supplementary Table 8), highlighting the utility of 1938 to uncover novel pathways potentially downstream of PI3Kα.

Compared to treatment with vehicle, insulin induced differential phosphorylation of 11 and 18 sites at 15 min and 4h, respectively, in PI3Kα-WT MEFs (Fig. 4a; Extended Data Fig. 7d), with substantial overlap in phosphosites regulated by 1938 and insulin at both time points (Fig. 4d). The majority of phosphosites upregulated by insulin at both timepoints were similar to the sites upregulated by 15 min 1938 treatment, whereas 4h treatment with 1938 induced phosphorylation of a larger set of sites (Fig. 4a,d), which might be due to a threshold effect, with a higher level of pAKT induced by 1938 compared to insulin at the concentrations of ligands tested (Extended Data Fig. 7b).

### 1938 induces cell proliferation

**Extended Data Fig. 8:**
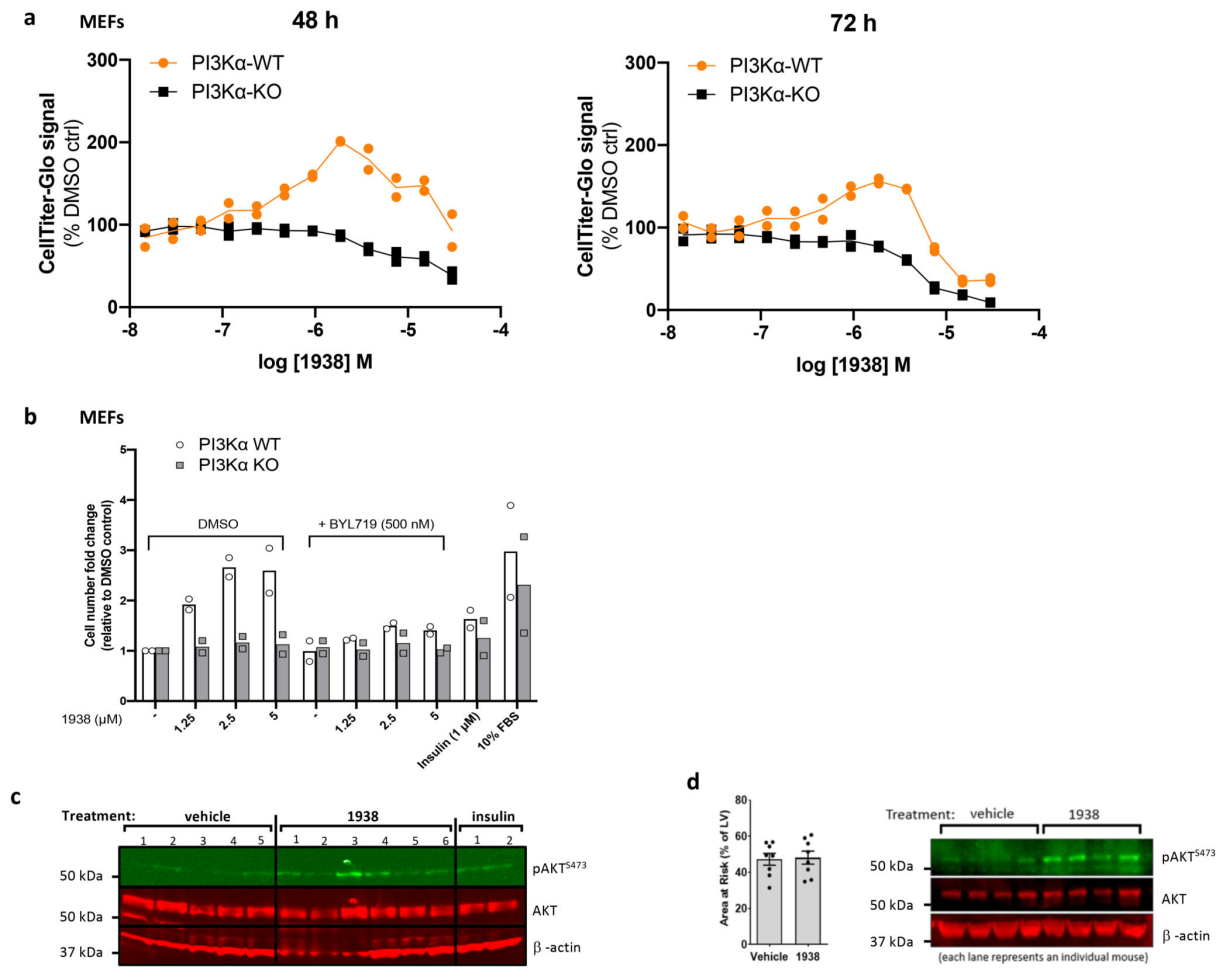
Additional data related to the functional activities of 1938 in cultured cells, tissues and organisms. **a**, Time-dependent dose-response of MEFs to 1938 as measured by CellTiter-Glo^®^. PI3Kα-WT and PI3Kα-KO MEFs were serum starved for 4 h, followed by stimulation with a dose range of 1938 in serum-free media for the indicated time points. Cellular metabolic activity was assessed by measurement of cellular ATP content by CellTiter-Glo^®^. Luminescence normalised to DMSO-only as 100%. Data shown from 2 individual experiments. **b**, MEFs were serum-starved overnight, followed by 24h stimulation in serum-free medium with 1938±BYL719, insulin, or culture medium containing 10% FBS, followed by measurement of cell number (crystal violet staining). Data show 2 independent experiments. **c**, *Ex vivo* perfused Langendorff rat heart model. Generation of pAKT^S473^ in ischaemic hearts treated with vehicle, 1938 or insulin upon reperfusion. Rat hearts were perfused for 10 min for stabilization, followed by 45 min global ischaemia and then reperfused for 2 h. During the first 15 min of reperfusion, the buffer contained either vehicle (0.1% DMSO), 1938 (5 μM) or insulin (1 μM). After 2 h, all hearts were freeze-clamped and frozen in liquid nitrogen followed by tissue extraction in RIPA buffer, SDS-PAGE and immunoblotting with the indicated antibodies. The quantification for this blot is shown in Fig. 5d. Statistics: 1-way ANOVA with Tukey’s post test. Each lane contains the extract of an individual heart: vehicle (n=5), 1938 (n=6) or insulin (n=2). **d**, *In vivo* perfused mouse heart model. Left panel, area at risk in vehicle- and 1938-treated hearts. Mice were subjected to 40 min coronary artery ligation followed by 2 h reperfusion. 15 min prior to reperfusion, 50 μl of DMSO or 10 mg/kg 1938 in DMSO was administered i.v. Following reperfusion, the hearts were then excised, perfused with Evans Blue, sliced and stained with tetrazolium chloride, prior to blinded assessment of infarct size as a percentage of the total ischaemic “area at risk” (AAR) (this is shown in Fig. 5e).The AAR in each heart is indicated as a % of the total area of the left ventricular (LV) myocardium. Since there was no significant difference in AAR between the two groups (P=0.86), this control measurement demonstrates experimental consistency in suture positioning etc. Statistics: Student’s unpaired 2-sided t-test, data shown as mean±SEM. Right panel, generation of pAKT^S473^ in ischaemic hearts treated with vehicle or 1938 upon reperfusion. 50 μl of DMSO vehicle or 10 mg/kg 1938 in DMSO was injected i.v. into anaesthetized and intubated mice. After 15 min, the chest was opened, the heart removed and immediately freeze-clamped in liquid nitrogen followed by tissue extraction in RIPA buffer, SDS-PAGE and immunoblotting with the indicated antibodies. Each lane contains the extract of an individual heart of mice treated with vehicle (n=4) or 1938 (n=4). The quantification for this blot is shown in Fig. 5e, right panel.

**Fig. 5:**
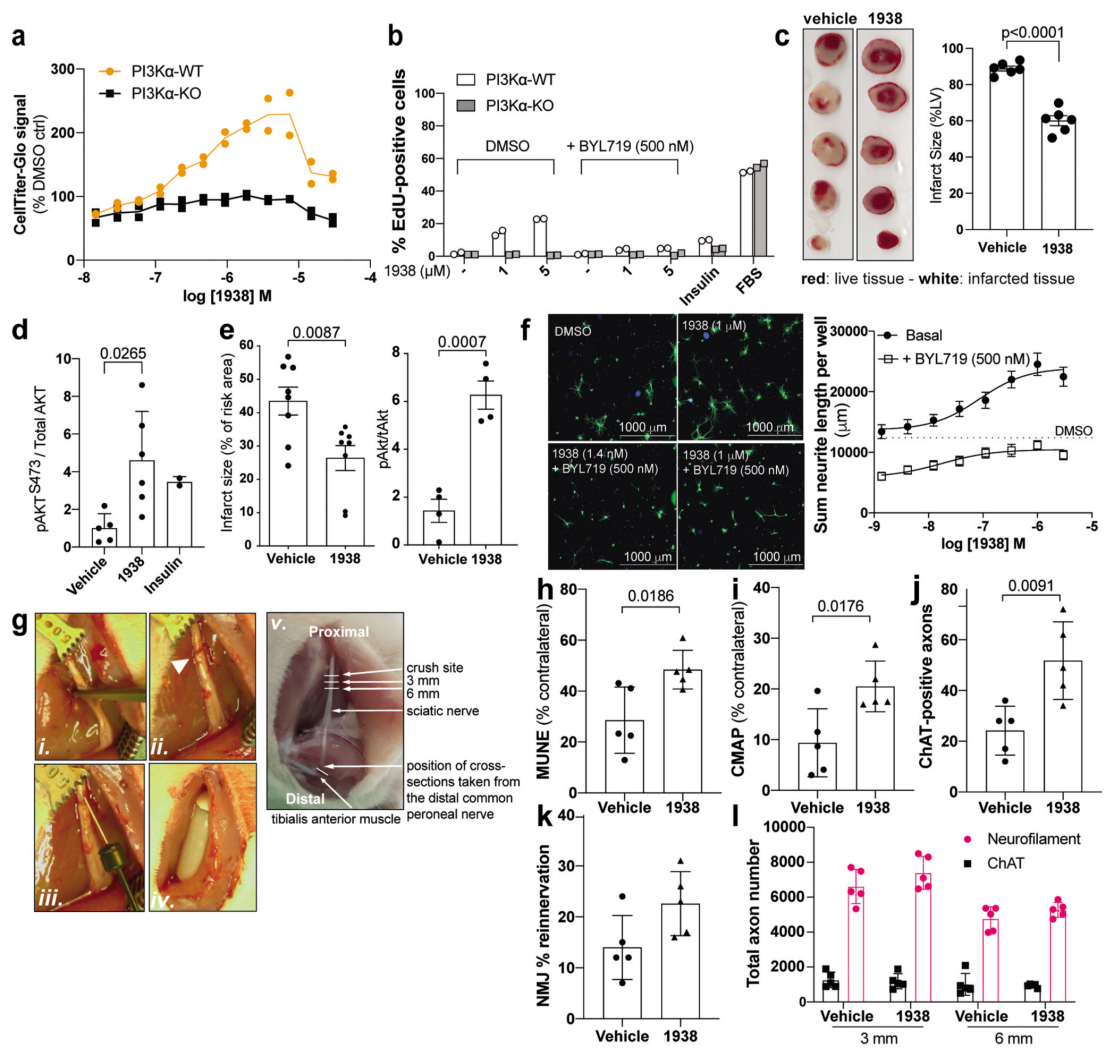
1938 induces biological responses in cultured cells, explanted tissues and model organisms. **a,b**, MEFs were serum-starved overnight, followed by 24h stimulation in serum-free medium with 1938±BYL719, insulin, or culture medium containing 10% FBS, followed by measurement of: **a**, metabolic activity (ATP content assessed by CellTiter-Glo^®^), **b**, cell cycle progression (EdU incorporation). (**a,b**) show 2 independent experiments. Gating strategies for (**b**) shown in Supplementary Fig. 2. **c**, *Left*, Representative tetrazolium-stained slices of isolated rat hearts (Langendorff model) subjected to 45 min global ischaemia, followed by 2h reperfusion, with administration of DMSO (0.1%) or 1938 (5 μM) during the first 15 min of perfusion. *Right*, infarct size measured at the end of the 2h reperfusion, in *ex vivo* hearts administered DMSO (n=6) or 1938 (n=6). Unpaired Student’s t-test. **d**, pAKT^S473^ in *ex vivo* hearts administered DMSO (n=5), 1938 (n=6) or insulin (n=2). 1-way ANOVA with Tukey post-test. **e**, Impact of 1938 on *in vivo* heart IRI in mice. *Left*, infarct size measured following 40 min ischaemia and 2h reperfusion, with DMSO (n=8) or 1938 (n=8) administered 15 min prior to reperfusion. Unpaired Student’s t-test. *Right*, pAKT^S473^ in hearts administered DMSO (n=4) or 1938 (n=4). Unpaired Student’s t-test. Data in **c-e** shown as mean±SEM (n=independent experiments). **f**, Neurite length in DRG cultures stimulated with 1938±BYL719 for 72h, with representative images of neurons stained with anti-β-III tubulin at 72h. Data represent mean±SEM of n=3 independent experiments. **g**, Sciatic nerve crush injury *(i)*, arrowhead in *(ii)* shows resulting lesion. Injury was followed by *(iii)* direct injection proximal to the injury, of a single dose of dH2O or 1938 (5 μM in sterile H2O) and *(iv)* minipump implantation for continuous delivery of dH2O or 1938 (100 μM in sterile H2O) for 21 days. **h**, Motor unit number estimation (MUNE) recordings from the *tibialis anterior* (TA) muscle. **i**, Compound muscle action potential (CMAP) recordings in the TA muscle following nerve stimulation proximal to the crush site (percentage of the contralateral side). **j**, Total number of choline acetyltransferase (ChAT)-positive motor axons in distal common peroneal nerve cross-sections. **k**, Proportion of neuromuscular junctions (NMJs) re-innervated by axons at the target TA muscle, revealed by α-bungarotoxin (α-BTX) and neurofilament (NF) staining. **l**, Quantification of total axons (neurofilament) and motor axons (ChAT) in the sciatic nerve at 3 and 6 mm distal to the injury site. For all experiments in **h-l**: n=5 animals per group, error bars are SD. Two-tailed Student’s t-tests. All data are from the 21 day endpoint.

In PI3Kα-WT but not in PI3Kα-KO MEFs, 24h treatment with 1938 dose-dependently increased metabolic activity, with an EC50 of ~0.5 μM (Fig. 5a), and a decrease in ATP levels in both PI3Kα-WT and PI3Kα-KO MEFs at concentrations >7.5 μM, indicative of PI3Kα-independent effects of 1938 at these doses (Fig. 5a). Upon 48h and 72h incubation, these non-PI3Kα-dependent 1938 effects were observed from 2-4 μM onwards (Extended Data Fig. 8a).

In PI3Kα-WT but not in PI3Kα-KO MEFs, 1938 induced cell cycle progression (Fig. 5b) and an increase in cell number (Extended Data Fig. 8b), which could be fully neutralised by co-treatment with BYL719. Unlike 1938, insulin did not induce cell cycle progression and an increase in cell number (Fig. 5b, Extended Data Fig. 8b), providing further evidence for a differential cellular impact of insulin and 1938 at the doses tested, as suggested by our proteomics data (Fig. 4a,d).

### 1938 provides cardioprotection

Myocardial infarction is responsible for significant morbidity and mortality in patients with coronary artery disease. Despite the development of new anti-platelet and anti-thrombotic agents, timely reperfusion by percutaneous coronary intervention via catheterisation remains fundamental to heart tissue salvage. Paradoxically, such reperfusion also causes IRI, tissue damage that occurs following the restoration of blood supply after a period without^14,48^, and is also observed in intra-arterial device-based treatment of stroke^12,13^. Finding ways to reduce IRI is vital to improving the long-term outcome of patients with myocardial infarction^14,48^ and stroke^12,13^. Ischaemic preconditioning, an experimental method of protecting the heart from IRI, leads to the activation of kinases including PI3K/AKT as part of the so-called Reperfusion Injury Salvage Kinase (RISK) pathway^11^, a cardioprotective signalling pathway induced by most cardioprotective agents^49^, including insulin, the canonical activator of PI3Kα^36,37,50^. Using PI3Kα inhibitors, we previously showed that PI3Kα activation is both necessary and sufficient for cardioprotection provided by ischaemic preconditioning or insulin^51^.

In *ex vivo* perfused rat hearts, 1938 was found to be a fast-acting agonist which, upon administration during the first 15 min of reperfusion, provided substantial tissue protection from IRI. This was evidenced by increased tissue survival and reduced infarct size (Fig. 5c), associated with an increase in generation of pAKT^S473^ (Fig. 5d; Extended Data Fig. 8c). 1938 also provided significant cardioprotection in an *in vivo* IRI model in mice, with a corresponding pAKT^S473^ increase in the hearts (Fig. 5e; Extended Data Fig. 8d). Given the observed rapid PI3Kα activation observed in both models, it could be envisaged that therapeutic application of a direct PI3Kα activator to a patient undergoing emergency coronary revascularization following myocardial infarction could be cardioprotective, and practically feasible in the clinical setting.

### 1938 stimulates nerve regeneration

PI3K pathway activation has been linked to neuroprotection and neuroregeneration^9,10,17-19,22,23,25^, with a positive role for PI3Kα recently demonstrated in axonal regeneration using genetic approaches^20^. There are currently no therapeutic agents used routinely to stimulate neuronal regeneration such as for injury to peripheral nerves, the spinal cord or optic nerves.

In a dose-dependent manner, 1938 significantly increased neurite outgrowth in dissociated adult rat dorsal root ganglion (DRG) cultures, an *in vitro* model for neuroregeneration, with higher 1938 concentrations doubling the total length of neurites measured at 72h (Fig. 5f). In the presence of low, biologically-inactive doses of 1938 (such as 10^-9^ M), BYL719 inhibited neurite outgrowth, and partially reduced the increase in neurite outgrowth induced by 1938 concentrations above 10^-7^ M (Fig. 5f).

**Extended Data Fig. 9:**
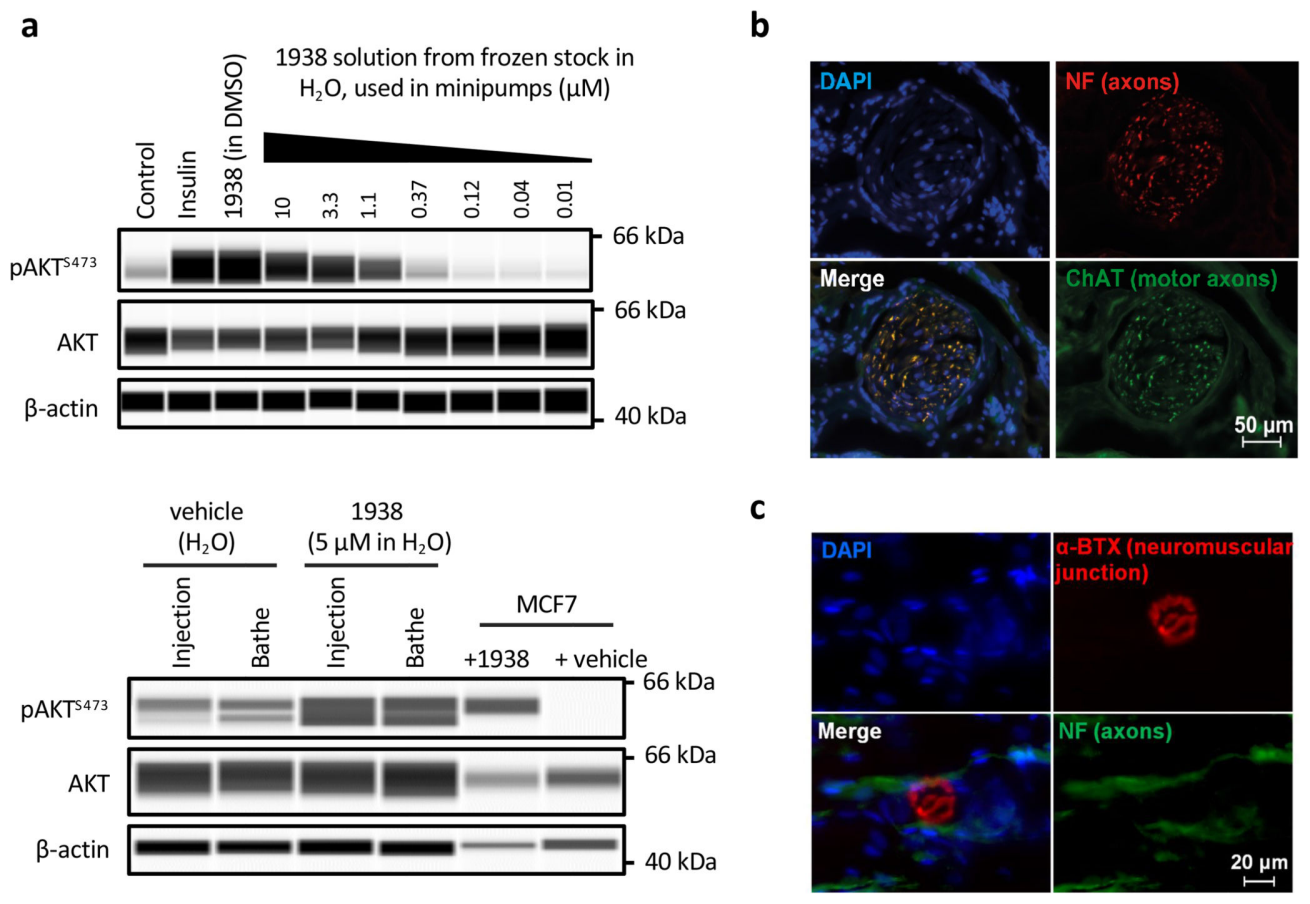
Additional and control studies for neuro-regeneration experiments. **a**, *Top panel;* Control experiment to test the biological activity of 1938 post-freezing. An aliquot of 100 μM 1938 stock solution in dH_2_O and vehicle was defrosted and tested for induction of pAKT^S473^ by 15 min treatment of A549 cells, using insulin (1 μM) or 1938 (10 μM from control stocks in DMSO) as positive controls. *Bottom panel;* pAKT^S473^ induction in exposed sciatic nerves, injected with vehicle (autoclaved H_2_O) or 1938 (from stocks in autoclaved H_2_O) or bathed in a solution of vehicle or 1938. After 30 min, the nerves were washed and processed for analysis as described in Materials and Methods. Cell extracts of MCF7 breast cancer cells stimulated for 15 min with 5 μM 1938 or vehicle (DMSO) were loaded on the gels as positive controls. n=1 experiment. **b,** Representative immunohistochemistry images of a transverse section through the distal common peroneal rat nerve, showing ChAT- and neurofilament-positive axons with tissue architecture typical of normal tissue. Scale bar = 50 μm. **c,** Representative immunohistochemistry images of rat TA muscle, showing a α-BTX-stained post-synaptic neuromuscular structure with associated neurofilament-positive neurons. Scale bar = 20 μm. n=5 animals.

We next tested 1938 in the rat sciatic nerve crush model of peripheral nerve injury and regeneration (Fig. 5g). Exploratory experiments showed pAKT induction upon direct injection of 1938 or bathing of exposed sciatic nerves in a 1938 solution (Extended Data Fig. 9a), indicating that 1938 leads to PI3K pathway activation in this tissue when delivered locally. Immediately after the nerve crush (Fig. 5g, *i-ii*), 1938 was delivered via a single intraneural injection into the proximal crush site (Fig. 5g, *iii*) and via a minipump implanted adjacent to the nerve (Fig. 5g, *iv*), loaded with 1938 solution, for continuous delivery for the duration of the experiment. Analyses were conducted 3 weeks after injury.

Electrophysiological recordings from the *tibialis anterior* muscle during nerve stimulation proximal to the injury site showed a greater electrophysiological recovery upon 1938-treatment, as indicated by an increased motor unit number estimation (MUNE) (Fig. 5h) and greater compound muscle action potential (CMAP) recovery (Fig. 5i). This correlated with histological analyses which showed an increase in 1938-treated animals in the number of choline acetyltransferase (ChAT)-positive motor axons (Fig. 5j); assessed in distal nerve sections from the common peroneal branch of the sciatic nerve, close to the point of re-innervation of the *tibialis anterior* muscle (indicated in Fig. 5g, *v*, i.e. approximately 25 mm from the injury site), with neurites grouped within normal fascicular nerve architecture (Extended Data Fig. 9b).

Histological analysis further showed innervation of a proportion of neuromuscular junctions (NMJs) in the *tibialis anterior* muscles (Fig. 5k), with the characteristic normal distribution of post-synaptic acetylcholine receptors and axons (Extended Data Fig. 9c).

Analysis after 21 days is an early time point in terms of regeneration, with low level initial re-innervation of muscle expected in untreated animals. The histological detection of motor axons in the distal nerve and NMJs corresponds with improved electrophysiological reinnervation of the *tibialis anterior* muscle. Histological analysis of nerve sections closer to the point of injury (3 and 6 mm distal to the crush site) showed equivalent numbers of neurofilament- and ChAT-positive axons in treatment and control groups (Fig. 5l). This indicates that the improved functional muscle re-innervation associated with 1938 treatment is due to an acceleration of natural neuronal regeneration rather than an increase in the overall number of regenerating neurites.

## Discussion

Here we report on a small molecule to directly and allosterically activate PI3Kα, providing a chemical tool to investigate the consequences of direct PI3Kα activation in basic and translational studies. This reagent will facilitate controlled studies to gain a better quantitative understanding of PI3Kα signalling^52^ and to delineate PI3Kα-specific signalling in cells. Our data also reveal the potential of PI3Kα-activating compounds like 1938 for use in tissue protection and regeneration, widening the possible therapeutic range of modulating this enzyme. However at present, we cannot exclude that some of the regenerative effects of 1938 *in vivo* are contributed by non-target-dependent effects, a challenge for any pharmacological modulator, especially at the first stages of development.

1938 is an allosteric activator of wild-type and all oncogenic PI3Kα mutants tested. 1938 binds outside the ATP-binding site, and weakens the inhibitory effects of p85α on p110α, contributing to enzyme activation. Its ability to induce conformational changes that do not fully overlap with those observed in PI3Kα activation by natural ligands (pY) or oncogenic *PIK3CA* mutations, indicates a unique mechanism of activation. The AA1016-1026 p110α helix, which is analogous to the PIKK regulatory domain (PRD) of PI3K-like protein kinases such as mTOR and ATM, acts as a transmission between the kinase/activator interface and the kinase active site. Binding of 1938 shifts this PRD-like helix to potentially reposition the activation loop and facilitate productive phospho-transfer to PIP_2_. The helical domain also pivots to bring the ATP-binding loop (AA772-776) and the N-lobe of the p110α kinase domain toward the ATP-binding site for beter phospho-transfer.

Our data indicate that *transient* PI3K activation using 1938 allows to effectively boost endogenous protective and regenerative signalling. PI3Kα signalling induced by 1938 and insulin showed similar kinetics in cells, including downregulation upon prolonged exposure, indicating that 1938-driven PI3Kα signalling remains subject to endogenous feedback mechanisms^46^. Such short-lived PI3K signalling is likely to differ from the *sustained* impact on signalling provided by constitutive oncogenic *PIK3CA* activation. Mutant *PIK3CA* on its own is a weak driver oncogene, with mice constitutively expressing the *Pik3ca^H1047R^* hot-spot mutation not developing cancer within a year^53,54^. Similarly, people with rare mosaic genetic activation of *PIK3CA* are not predisposed to cancer in adulthood, although it has to be noted that *PIK3CA* mutations in these patients are present in different tissues than the tissue types with somatic *PIK3CA* mutations in sporadic cancer^55^. Taken together, these data make it less likely that short-term and transient pharmacological PI3Kα activation would induce or promote cancer.

In general, our work illustrates the potential of activating kinases for therapeutic benefit, a currently largely unexplored area of drug development.

## Methods

Compound UCL-TRO-1938 is available from https://www.cancertools.org/ catalog No. 161068.

### HTS

A HTS for human p110α/p85α (referred to as PI3Kα) of 450,000 small molecules in the AstraZeneca screening library was performed using the ADP-Glo™ kinase assay. To enable the HTS, the PI3Kα ADP-Glo™ kinase assay was miniaturised into 1536-well format. Reagent stability and compatibility with low volume dispensing technology were optimised to ensure conformity with HTS conditions. The HTS was performed in single point at room temperature using white 1536-well plates (Corning #3729). DMSO-solubilised compounds were acoustically dispensed to assay ready plates using an Echo 555 (Labcyte) yielding a final compound concentration of 10 μM for most compounds (with some low MW compounds screened at 100 μM) and a final DMSO concentration of 1%. The reaction mixture contained 750 nl enzyme, 750 nl substrate. Final concentrations of reaction buffer, PI3Kα, liposomes and ATP used were unchanged. PI3Kα (25 nM) was incubated for 3 h at room temperature with 500 μM ATP and liposomes (1 mg/ml) that mimic the plasma membrane lipid composition, enriched with 5% PtdIns(4,5)P2 substrate, using the ADP-Glo™ assay (Promega) to measure ADP production. Compound responses were normalised to DMSO control, and 6000 hit compounds with activity >3x standard deviation of the DMSO control were re-screened in duplicate, using the assay described above. The final assay hit rate was 0.53%. The HTS experimental procedure and data analysis were performed at AstraZeneca using proprietary processes.

Confirmed hits with near neighbour molecules were subsequently screened in a 10-point concentration response curve using a bis-phosphorylated phosphopeptide (a PDGF-receptor-derived peptide phosphorylated on Tyr-740 and Tyr-751, hereafter referred to as pY peptide^29^) to mimic receptor tyrosine kinase binding to p85α as a positive control.

Confirmed hits and near neighbour compounds were subsequently screened using an orthogonal fluorescence polarisation biochemical activity assay in 10-point concentration response curves. Biophysical confirmation of PI3Kα binding of selected hits was assayed by microscale thermophoresis, and hits that bound PI3Kα investigated for cellular activity using the A549 human lung carcinoma cell line, measuring the generation of phospho-S473-AKT (further referred to as pAKT^S473^) by automated Wes western blotting or ELISA. Routine compound profiling of novel compounds was performed using the ADP-Glo™ *in vitro* kinase assay and ELISA for pAkt generated in A549 cells.

### Lipid substrate preparation for HTS

A plasma membrane-like composition of liposomes was prepared by combining L-α-phosphatidylinositol-4,5-bisphosphate (Brain, Porcine, Avanti #840046X), L-α-phosphatidylserine (Brain, Porcine, Avanti 840032C), L-α-phosphatidylethanolamine (Brain, Porcine, Avanti #840022C), L-α-phosphatidylcholine (Brain, Porcine, Avanti #840053C), cholesterol (Ovine Wool, Avanti #700000P) and sphingomyelin (Brain, Porcine, Avanti #860062C) in a 5:20:45:15:10:5 ratio while in organic solvent (primarily a chloroform:methanol:water (9:3:1) mixture). Methanol was titrated into the mixture until components were in solution. The liposome solution was then placed on a rotatory evaporator flushed with nitrogen gas, and solvent was evaporated at 250 mbar using a 25°C water bath, until a translucent film of lipids was observed. The container was flushed with nitrogen before being placed under vacuum for a further 16 h. Lipid buffer (20 mM HEPES pH 7.5, 100 mM KCl, 1 mM EGTA pH 8.0) was then added, and the flask vortexed until the lipids were in suspension. The flask was then bath-sonicated for 2 min, before being aliquoted into 250 ml plastic flasks. These fractions were freeze-thawed 11 times using liquid nitrogen and a 42°C water bath. Liposomes were then extruded using the Avestin LF-50 liposome extrusion apparatus. Liposomes were extruded with nitrogen gas at a pressure of 150 psi. 50 ml aliquots of liposome solution were initially extruded through a 0.4 μm filter, followed by five passes through a 0.25 μm filter. Liposome solutions were then flash frozen in liquid nitrogen and stored at -80°C.

### Lipid substrate preparation for post-HTS ADP-Glo™ kinase assay

Liposomes were prepared by mixing lipid components dissolved in chloroform and then evaporating the solvent under a stream of nitrogen gas. The remaining lipid film was dried under a vacuum for 2 h, then resuspended in liposome buffer (20 mM HEPES, 100 mM KCl, 1 mM EGTA, pH 7.5). The lipid solution was vortexed for 3 min and sonicated in a water bath for 2 min at room temperature. The clarified solution was then subjected to 11 freeze-thaw cycles of snap freezing in liquid nitrogen followed by thawing in a 42°C water bath. Liposomes were created by extruding 11 times through a 100 nm filter, snap frozen in liquid nitrogen and stored at -80°C.

### PI3K protein expression and purification

Full-length human p110α was expressed either in a complex with full-length human p85α (for HTS, biochemistry and HDX-MS) or with p85-niSH2 (amino acids 307-593) (for crystallography). A p110α construct (AA 105-1048)^30^ lacking the adaptor binding domain and lipid binding surface was also used for crystallography

Expression and purification of wild-type p110α (Cambridge MRC Laboratory for Molecular Biology (LMB-MRC) plasmid number OP831) in a complex with full-length p85α (LMB-MRC plasmid OP809) was performed as described^29^. The oncogenic mutants G106V (LMB-MRC plasmid JB35), N345K (LMB-MRC plasmid OP661) and E545K (LMB-MRC plasmid OP663) were also purified using this protocol. Briefly, 10 litres of *Spodoptera frugiperda* (Sf9) cell culture at a density of 1.0 x 10^6^ cells/ml were co-infected with a p85α-encoding virus [LMB-MRC plasmid OP809]. and a virus encoding p110α with an N-terminal 6xHis tag followed by a tobacco etch virus (TEV) protease site [LMB-MRC plasmid OP831]. After a 48 h infection at 27°C, cells were harvested and washed with PBS. Cell pellets were then resuspended in Lysis Buffer (20 mM Tris pH 8.0, 300 mM NaCl, 5% glycerol, 10 mM Imidazole pH 8.0, 2 mM β-mercaptoethanol, 1 EDTA-free protease inhibitor tablet (Roche) per 50 ml of buffer) and sonicated at 4°C for 7 min in 15 sec intervals followed by a 15 sec wait. Cell lysate was then centrifuged at 45,000 g for 45 min at 4°C. Supernant was then filtered using a 0.45 μM filter before being passed over 2 x 5 ml HisTrap FF (Cytiva) Columns (equilibrated in NiNTA Buffer [20 mM Tris pH 8.0, 300 mM NaCl, 5% glycerol, 10 mM imidazole (pH 8.0), 2 mM β-mercaptoethanol]) at a 3 ml/min flow rate. Columns were then washed using a 20 mM imidazole wash, and protein was eluted in a gradient to NiNTA B Buffer (20 mM Tris pH 8.0, 300 mM NaCl, 5% glycerol, 200 mM imidazole (pH 8.0), 2 mM β-mercaptoethanol). PI3Kα containing fractions were then pooled and diluted 1:2 with Salt Dilution Buffer (20 mM Tris pH 8.0, 1 mM DTT) to reduce NaCl concertation to 100 mM. This solution was then passed over a HiTrap Heparin (Cytiva) Column (equilibrated in Hep A Buffer (20 mM Tris pH 8.0, 100 mM NaCl, 2 mM β-mercaptoethanol)) at a rate of 3 ml/min. PI3Kα was eluted using a gradient to HEP B Buffer (20 mM Tris pH 8.0, 1 M NaCl, 2 mM β-mercaptoethanol). Protein containing fractions were then pooled and concentrated to 8 mg/ml, before being loaded onto a Superdex 200 16/60 column, equilibrated in Gel Filtration Buffer (20 mM HEPES pH 7.4, 100 mM NaCl, 2 mM TCEP), run at 1 ml/min at 4°C. PI3Kα-containing fractions were pooled and concentrated to 2.5 mg/ml before being flash-frozen in liquid nitrogen and stored at -80°C.

Expression and purification of full-length human p110α (carrying the M232K and L233K mutations used in the structure determination for PDB ID 4JPS^28,56^), in complex with human p85α-niSH2 (amino acids 307-593), was performed as follows. Sf9 insect cells were cultured in Insect-XPRESS with L-Glutamine medium (Lonza BE12-730Q) at 27°C and infected with baculovirus encoding both p110α and p85α-niSH2 [LMB-MRC plasmid GM129] at a density of 1.6–1.8× 10^6^ cells/ml. The culture was incubated for 48 h after infection, and cells were collected and washed with PBS, flash-frozen in liquid N2 and stored at -80°C. For purification, cell pellets were resuspended in 100 ml of lysis buffer (20 mM Tris, 150 mM NaCl, 5% glycerol, 2 mM β-mercaptoethanol, 0.02% CHAPS, pH 8.0) containing EDTA-free Protease inhibitor tablets (Roche, 1 tablet per 50 ml of solution) and 500 μl DNAse I. The suspension was sonicated for 10 min on ice, with 10 sec on and 10 sec off. The lysate was then centrifuged at 35,000 rpm for 45 min using a Ti45 rotor at 4°C. The samples were loaded onto a StrepTrap (Cytiva) column in S300 buffer (20 mM Tris, 300 mM NaCl, 5% glycerol, 2 mM TCEP, pH 8.0). Once the protein was loaded, the column as washed with buffer A (20 mM Tris, 100 mM NaCl, 5% glycerol, 1 mM TCEP, pH 8.0). The column was eluted using a gradient from 1-100% buffer B (buffer A containing 5 mM *d*-Desthiobiotin). Fractions of the p110α/p85α-niSH2 peak were pooled and TEV protease (0.8 mg/ml) was added at the ratio of 1:10 and left at 4°C to cleave overnight. Protein was loaded onto a 5 ml HiTrap Heparin HP column (Cytiva) washed with buffer A, and eluted with a gradient of 1-100% buffer C (20 mM Tris, 1 M NaCl, 1 mM TCEP, pH 8.0). The fractions were collected, concentrated and loaded on a Superdex 200 26/60 HiLoad gel filtration column (Cytiva) and eluted in 20 mM Tris, 200 mM NaCl, 2 mM TCEP, 1% betaine, 1% ethylene glycol and 0.02% CHAPS, pH 7.2. The peak fractions were pooled and concentrated to 10-13 mg/ml using Amicon Ultra-15 Centrifugal filters 100K (Millipore), as measured by a NanoDrop at 280 nm. The protein was then flash-frozen in liquid nitrogen and stored at -80°C. Purity of protein was checked using SDS-PAGE.

Expression and purification of truncated human p110α (105-1048) were performed as follows. Sf9 insect cells (9 L) were cultured in Insect-XPRESS with L-Glutamine medium (Lonza BE12-730Q) at 27°C and infected with baculovirus encoding the p110α subunit [LMB-MRC plasmid OP798] at a density of 1.6 × 10^6^ cells/ml. The culture was incubated for 48 h after infection, cells were collected, flash-frozen in liquid N2 and stored at -80°C. For purification, cell pellets were resuspended in 360 ml of lysis buffer (20 mM Tris, 150 mM NaCl, 5% glycerol, 1 mM TCEP, pH 8.0) containing EDTA-free Protease inhibitor tablets (1 tablet per 50 ml of solution), 0.5 mM PEFA and 36 μl of Piece® Universal Nuclease For Cell Lysis. The suspension was sonicated for 5 min on ice, with 10 sec on and 10 sec off. The lysate was then centrifuged at 35,000 rpm for 35 min using a Ti45 rotor at 4°C. The samples were filtered through a 5 μm filter and loaded onto a StrepTrap (Cytiva) column equilibrated in lysis buffer. Once the sample was loaded, the column was washed with 20 mM Tris, 300 mM NaCl, 5% glycerol, 1 mM TCEP, pH 8.0, and then with 20 mM Tris, 150 mM NaCl, 5% glycerol, 1 mM TCEP, pH 8.0. Then 5 ml TEV solution at 0.14 mg/ml was added onto the column and left at 4°C to cleave overnight. Protein was loaded onto a 5 ml HiTrap Heparin HP column (Cytiva) equilibrated in 20 mM Tris, 150 mM NaCl, 5% glycerol, 1 mM TCEP, pH 8.0, and eluted with a gradient of 1-100% of 20 mM Tris, 1 M NaCl, 1 mM TCEP, pH 8.0. The fractions were collected, concentrated and loaded on a Superdex 200 16/60 HiLoad gel filtration column (Cytiva) and eluted in 50 mM Tris, 100 mM NaCl, 2% ethylene glycol, and 1 mM TCEP, pH 8.0. The peak fractions were pooled and concentrated to 5.83 mg/ml using Amicon Ultra-15 Centrifugal filters 50K (Millipore), as measured by a NanoDrop at 280 nm. The protein was then flash-frozen in liquid nitrogen and stored at -80°C. Purity of protein was checked using SDS-PAGE.

Expression and purification of mutants resistant to 1938 were performed as follows. Sf9 insect cells (1.5 L) were cultured in Insect-XPRESS with L-Glutamine medium (Lonza BE12-730Q) at 27°C and co-infected with baculovirus encoding the regulatory p85α-subunit [LMB-MRC plasmid OP809] and the catalytic subunit p110α-D603K [LMB-MRC plasmid OP895], D603A [OP900], 603DCN_AAA605 [OP894], D603A/F1016S [OP901], L1006R [OP897], F1016S [OP898], L1006R/F1016S [OP899] at a density of 1.6 × 10^6^ cells/ml. The culture was incubated for 47 h after infection, cells were collected, flash-frozen in liquid N2 and stored at -80°C. For purification, cell pellets were resuspended in 50 ml of lysis buffer (20 mM Tris, 150 mM NaCl, 5% glycerol, 1 mM TCEP, pH 8.0) containing EDTA-free Protease inhibitor tablets (1 tablet per 50 ml of solution), 0.5 mM PEFA and 5 μl of Piece® Universal Nuclease For Cell Lysis. The suspension was sonicated for 3 min on ice, with 10 sec on and 10 sec off. The lysate was then centrifuged at 35,000 rpm for 35 min using a Ti45 rotor at 4°C. The samples were filtered through a 5 μm filter and loaded onto a StrepTrap (Cytiva) column equilibrated in lysis buffer. Once the sample was loaded, the column was washed with 20 mM Tris, 300 mM NaCl, 5% glycerol, 1 mM TCEP, pH 8.0, and then with 20 mM Tris, 150 mM NaCl, 5% glycerol, 1 mM TCEP, pH 8.0. Then 5 ml TEV solution at 0.14 mg/ml was added onto the column and left at 4°C to cleave overnight. Protein was loaded onto a 5 ml HiTrap Heparin HP column (Cytiva) equilibrated in 20 mM Tris, 150 mM NaCl, 5% glycerol, 1 mM TCEP, pH 8.0, and eluted with a gradient of 1-100% of 20 mM Tris, 1 M NaCl, 1 mM TCEP, pH 8.0. The fractions were collected, concentrated and loaded on a Superdex 200 16/60 HiLoad gel filtration column (Cytiva) and eluted in 20 mM HEPES pH 7.5, 150 mM NaCl, 1 mM TCEP. The peak fractions were pooled and concentrated using Amicon Ultra-4 Centrifugal filters 50K (Millipore). The protein was then flash-frozen in liquid nitrogen and stored at -80°C. Purity of protein was checked using SDS-PAGE.

Full-length human p110β/p85α and p110δ/p85α were cloned and expressed in a similar manner. Briefly, 5 litres of *Spodoptera frugiperda* (Sf9) cell culture at a density of 1.0 x 10^6^ cells/ml were co-infected with both a p85α-encoding virus and a virus encoding p110β/δ with an N-terminal Strep-tag followed by a tobacco etch virus (TEV) protease site (plasmid OP832 for p110β, plasmid OP833 for p110δ and plasmid of OP809 for p85α). After a 48 h infection at 27°C, cells were harvested and washed with PBS. Cell pellets were then resuspended in Lysis Buffer (20 mM Tris pH 8.0, 150 mM NaCl, 5% glycerol, 2 mM β-mercaptoethanol, 1 EDTA-free protease inhibitor tablet (Roche) per 50 ml of buffer) and sonicated ar 4°C for 7 min in 15 sec intervals followed by a 15 sec wait. Cell lysate was then centrifuged at 45,000 *g* for 45 min at 4°C. Supernatant was then filtered using a 0.45 μm filter before being passed over 1 x 5 ml Streptrap No 1 (GE Healthcare) Columns (equilibrated in 100S Buffer [20 mM Tris pH 8.0, 100 mM NaCl, 5% glycerol, 1 mM TCEP]) at a 3 ml/min flow rate. Column was then washed using 70 ml 100S Buffer, followed by 80 ml S300 Buffer (20 mM Tris pH 8.0, 300 mM NaCl, 5% glycerol, 1 mM TCEP) followed by 50 ml S100 Buffer. 5 ml of 0.1 mg/ml His6TEV protease in S100 Buffer was injected onto the column and was incubated at 4°C for 4 h. The column was then attached to a Heparin column, and the purification protocol proceeded as for PI3Kα.

### Fluorescence polarization assay

PIP_3_ production was measured using a fluorescence polarization assay (#K-1100; Echelon Biosciences, Salt Lake City, UT, USA) and carried out in 384-well microtitre plates. PI3Kα, liposomes and ATP were all diluted in the reaction buffer (20 mM HEPES, 50 mM NaCl, 50 mM KCl, 3 mM MgCl_2_, 1 mM EGTA, 1 mM TCEP, pH 7.4) and added to the microtitre plate at a final reaction concentration of 10 nM PI3Kα, 75 μg/ml liposomes and 10 μM ATP. The reaction was carried out for 45 min at room temperature and quenched with the PIP_3_ detector and TAMRA probe, before being read in a Hidex Sense platereader using λ544±20 and λ590±20 polarizing filters. Data was normalised to the TAMRA probe alone and TAMRA plus detector for minimum and maximum PIP_3_ production, respectively.

### Microscale thermophoresis

MST experiments were performed using an automated Monolith NT.115 (NanoTemper Technologies, Munich, Germany). Fluorescence labelling of PI3Kα with the NT647 dye was performed in accordance with manufacturer protocol using the RED-NHS protein labelling Kit (NanoTemper Technologies, Munich, Germany). PI3Kα was diluted to a final concentration of 2.5 nM in reaction buffer (20 mM HEPES, 100 mM NaCl, 0.1% Tween-20 and 2 mM TCEP, pH 7.4). Compounds were serially diluted in neat DMSO and added to the enzyme to a final concentration of 3% DMSO. Premium treated capillaries, IR laser powers of 80% and LED intensity of 10% were used. Data was analysed with the NanoTemper Analysis software with ΔF_norm_ values (ΔF_norm_ = F_hot_/F_cold_) used to define compound binding.

### ADP-Glo™ kinase assay

Kinase reactions were performed with ADP-Glo kinase assay kit (Promega Corporation). The enzyme, substrate and compounds were diluted in reaction buffer (20 mM HEPES, 50 mM NaCl, 50 mM KCl, 3 mM MgCl_2_, 1 mM EGTA, 1 mM TCEP, pH 7.4). Final concentrations of PI3Kα and PI3Kδ used were 25 nM and 50 nM for PI3Kβ. Liposomes (5% brain PI(4,5)P2, 20% brain phosphatidylserine, 45% brain phosphatidylethanolamine, 15% brain phosphatidylcholine, 10% cholesterol, 5% sphingomyelin (Avanti Polar Lipids)) were used at a final concentration of 1 mg/ml.

For the HTS, the reaction mixture contained 0.75 μl enzyme, 15 nl compound and/or pY and 0.75 μl of liposome substrate mixed with ATP. The pY sequence was ESDGG(pY)MDMSKDESID(pY)VPMLDMKGDIKYADIE (GL Biochem, Shanghai Ltd). ATP was used at a final concentration of 500 μM unless otherwise stated. The final DMSO concentration in the assay was 1%. The experiments were performed at room temperature for 3 h using 1536-well white-polystyrene plates (Corning #3729) before addition of 2 μl of ADP-Glo R1 to terminate the reaction. The plate was incubated for 40 min, followed by addition of 4 μl of ADP-Glo R2 and incubated further for 60 min in the dark. Luminescence was read using an EnVision (PerkinElmer) plate reader. All analyses were performed using Genedata Screener.

For re-screening of HTS hits and routine compound profiling, the reaction mixture contained 2 μl PI3K enzyme, 2 μl compound and/or pY and 2 μl of liposome substrate mixed with ATP. ATP was used at a final concentration of 500 μM for PI3Kα and PI3Kβ and at 200 μM for PI3Kδ, unless otherwise stated. The final DMSO concentration in the assay was 1%. The experiments were performed at room temperature for 3 h using 384 white-polystyrene plates (Corning #3824) before addition of 6 μl of ADP-Glo R1 to terminate the reaction. The plate was incubated for 45 min, followed by addition of 12 μl of ADP-Glo R2 and incubated further for 60 min in the dark. Luminescence was read using a Sense (Hidex) plate reader. Compound data were corrected to the no enzyme DMSO negative control and expressed as a percentage of the internal positive control (1 μM pY), equivalent to maximal activation (E_max_). All analyses were performed using GraphPad Prism 7.

For characterisation of the effects of 1938 on *in vitro* PI3K enzymology, all reactions were performed at room temperature with 384 white-polystyrene plates (Corning #3574). The final DMSO concentration in the assay was between 0.5%-1.8%. The reaction mixture contained 2 μl PI3K enzyme, 2 μl compound and/or pY and 2 μl of liposome substrate mixed with ATP. ATP was used at a final concentration of 200 μM, unless otherwise stated. The enzyme and compounds were pre-incubated for 10 min prior to addition of substrate. The reaction was allowed to proceed for up to 45 min at room temperature, before addition of 6 μl of ADP-Glo R1 to terminate the reaction. The plate was incubated for 60 min, followed by addition of 12 μl of ADP-Glo R2 and incubated further for 60 min in the dark. Data was expressed as velocity (nmol of ADP generated/nmol of enzyme/sec). ADP-ATP standard curves were performed according to the manufacturer’s instructions. Luminescence was read using a PHERAstar^®^ plate reader with software version 5.41, and analyses were performed using GraphPad Prism 8/9.

### FRET membrane binding assay

Membrane binding assays were performed as previously published^29^. Briefly, liposomes were prepared with 5% (w/v) brain PtdIns(4,5)P2, 20% brain phosphatidylserine, 35% brain phosphatidylethanolamine, 15% brain phosphatidylcholine, 10% cholesterol, 5% sphingomyelin, and 10% dansyl-phosphatidylserine (Avanti Polar Lipids). PI3Kα was used at a final concentration of 0.5 μM. Protein solutions were preincubated with 10 μM pY or compounds for 10 min before addition of liposomes. Liposomes were used at a final concentration of 50 μg/ml. The reaction mixture contained 5 μl enzyme, 2 μl compound and 3 μl liposomes, all diluted in 30 mM HEPES, 50 mM NaCl, pH 7.4. The reaction was allowed to proceed for 10 min at room temperature with 384 black-polystyrene plates (Corning #3544) on an orbital shaker at 200 rpm. FRET signals were measured using PHERAStar (BMG) with a 280 nm excitation filter with 350 nm and 520 nm emission filters to measure Dansyl-PS FRET emissions, respectively. FRET signal shown as I-I0, where I is the intensity at 520 nm, and I0 is the intensity at 520 nm for the solution in the absence of protein.

### Surface Plasmon Resonance

SPR was performed with a Biacore T200, using CM7-sensor chips (Cytiva). Both reference control and analyte channels were equilibrated in HBS-P (Cytiva) supplemented with 5% (v/v) DMSO at 20°C. Full length p110α/p85α was immobilised onto the chip surface *via* amide coupling using the supplied kit (Cytiva) to reach an RU value of approximately 25,000 RU. Serial dilutions (1:2) of 1938 starting from 500 μM were injected over the chip for 60 s at 30 μL/min, with a 60 sec dissociation time. The data were double-referenced to the response on a blank but similarly modified flow channel and a buffer-only injection was subtracted. Any differences in the DMSO concentrations between the sample and buffer were corrected using the in-built solvent correction protocol. After reference and buffer signal correction, sensogram data were fitted using Prism 9.4.1 (GraphPad Software Inc). The responses at equilibrium (R_eq_) of the were then fitted to a 1:1 binding model with a linear non-specific phase to determine *K*_d_:

(1) $$R_{eq}=\left(\frac{CR_{\max}}{C+K_{d}}\right)+DC+B$$

where C is the analyte concentration and R_max_ is the maximum response at saturation, D is a non-specific response and B is the background resonance. Data were replotted correcting for the linear non-specific response. The binding was performed in triplicate.

### Differential Scanning Fluorimetry

Thermal denaturation was followed using intrinsic protein fluorescence measured with the NanoTemper Prometheus NT48 instrument (Nanotemper Technologies, München, Germany). Samples in HBS-P (Cytiva) supplemented with 5% (v/v) DMSO containing 3 μM full length p110α/p85α and a 1:2 dilution series of 1938 (from 440 μM to 13.8 nM) were loaded into standard capillaries and heated at 2 °C/min from 15 to 95 °C. The first derivative of the fluorescence emission ratio 350/330 nm were analyzed using the PR.ThermControl v2.3.1 (NanoTemper), to define the Tm. Independent experiments using the same protein and compound stocks were performed in triplicate. Data were fitted using Prism 9.4.1 (GraphPad Software Inc). Dissociation constants were calculated using fits to a single-site ligand depletion model:

(2) $$T=T_{0}+\frac{(T_{1}-T_{0})\{([C_{T}]+[P_{T}]+[K_{D}])-\sqrt{{\left([C_{T}]+[P_{T}]+K_{D}\right)}^{2}-4[C_{T}][P_{T}]\}}}{2[P_{T}]}$$

where *T_0_* and *T_1_* are the T_m_ in the absence of titrating compound and at saturation respectively, *[P_T_]* and *[C_T_]* are the total concentrations of protein and compound respectively and *K_D_* is the dissociation constant.

### HDX–MS

Sample preparation: HDX-MS experiments were carried out as described previously^57^. Briefly, 4 μM PI3Kα was incubated with in the absence of compound or with 250 μM 1938, or 5 μM BYL719, or both in a 2.5% DMSO-containing Protein Dilution Buffer (50 mM Tris pH 7.5, 150 mM NaCl, 2 mM DTT). 5 μl PI3Kα either with or without compound was then incubated with 45 μl D2O Buffer (50 mM Tris pH 7.5, 150 mM NaCl, 5 mM DTT, 2.5% DMSO with or without 125 μM 1938/ 2.5 μM BYL719, 90.6% D2O) for 5 timepoints (0.3 sec/3 sec/30 sec/300 sec/3000 sec, with the 0.3 sec timepoint being a 3 sec timepoint conducted at 0°C) before being quenched with 20 μl ice-cold Quench Solution (8 M Urea and 0.8% Formic Acid), and being rapidly snap-frozen in liquid nitrogen prior to storage at -80°C. In total, three biological replicates, i.e. three separate protein preparations, each with exchange experiments carried out in triplicate were conducted. Data presented in the manuscript is a single biological replicate. Data acquisition and analysis were as follows: Each sample was thawed and injected onto an M-Class Acquity UPLC with HDX Technology (Waters) kept at 0.1°C. Proteins were digested in-line using an Enzymate Pepsin Column (Waters, #186007233) at 15°C for 2 min at 200 μl/min. Peptic peptides were then eluted onto an Acquity UPLC BEH C18 Column (Waters, #186002346) equilibrated in Pepsin-A buffer (0.1% formic acid) and separated using a 5-35% gradient of Pepsin-B buffer (0.1% formic acid, 99% acetonitrile) over 7 min at a flowrate of 40 μl/min. Data were collected on a Waters Cyclic IMS, with an electrospray ionisation source, from 50-2000 m/z. Data were collected in the HDMS^E^ mode. A single pass of the cyclic IMS was conducted. A “blank” sample of protein dilution buffer with quench was run between samples, and carry-over of peptides was routinely monitored. Five replicates were used to identify non-deuterated peptides. Criteria used to include peptides in the HDX-MS dataset were: minimum intensity 5000, minimum sequence length 5, maximum sequence length 25, a minimum of 3 fragment ions, a minimum of 0.1 products per amino acid, a minimum score of 6.62, a maximum MH+ Error of 10 ppm, identification in at least two datasets with a retention time RSD of less than 10%. Data was analysed using Protein Lynx Global Server (Waters) and DynamX (Waters). All peptides were manually inspected for EX1 kinetics and sufficient quality of the peptide envelope. Data quality, experiment design, and reporting of data meets the criteria as determined by the HDX-MS community^58^. Uptake files were created using Baryonyx. Data are available via ProteomeXchange with identifier PXD037721.

### *In vitro* kinase profiling, mTORC1 and ATM kinase assays

133 protein kinases and 7 lipid kinases were counterscreened, with 1938 used at 1 μM, using the Adapta, Lantha and Z-LYTE assays (SelectScreen Kinase Profiling Service; Thermofisher – experimental details of these assays can be found here: https://www.thermofisher.com/uk/en/home/industrial/pharma-biopharma/drug-discovery-development/target-and-lead-identification-and-validation/kinasebiology/kinase-activity-assays.html. The tree represention in KinMap^59^ generated courtesy of Cell Signaling Technology, Inc. (www.cellsignal.com). mTORC1 (mTOR/RAPTOR/LST8) protein complex and ATM kinase and substrates were produced as previously described^57,60^. Screening of 1938 was conducted using SuperSep Phos-Tag 50 μmol/l 100 x 100 x 6.6 mm 17-well (192-18001/199-18011) gels. For ATM assays, 100 nM ATM was incubated for 30 min at 30°C with 5 μM GST-p53 and 1 mM ATP, in the absence or presence of 200 μM 1938 in ATM Kinase Buffer (50 mM HEPES pH 7.5, 100 mM NaCl, 10% glycerol, 2 mM Trichloroethylene, 5 mM MgCl_2_). As a positive control for ATM activation, the same reaction was carried out with 100 nM ATM/5 μM GST-p53/1 mM ATP in the presence of 100 nM Mre11-Rad50-Nbs1 (MRN) complex, a known activator of ATM^61^. For mTORC1 assays, 50 nM mTORC1 complex (mTOR/LST8/RAPTOR) was incubated for 3 h at 30°C with 15 μM 4E-BP1, 10 mM MgCl_2_ and 250 μM ATP, in the absence or presence of 200 μM 1938. As a ‘positive’ control, a higher concentration (150 nM) of mTORC1 complex (mTOR/LST8/RAPTOR) was incubated for 3 h at 30°C with 15 μM 4E-BP1, 10 mM MgCl_2_ and 250 μM ATP. Kinase reactions were quenched by addition of SDS-PAGE Loading Buffer (as per manufacturer’s instructions) and freezing at -20°C before being run on the Phos-tag gels at 150 V for 90 min. Gels were then stained using InstantBlue™ Coomassie, and quantified using BioRad Image Lab Software. Kinase assays were carried out in triplicate.

### Co-crystallisation of p110α/p85α niSH2-compound complexes

All crystallisation experiments were performed at a temperature of 20°C. An initial screen of approximately 2000 conditions was performed using the LMB robotic crystallization setup^62^. p110α/p85α niSH2 was either pre-incubated with 100 μM of BYL719 for 1 h, or pre-incubated with 100 μM BYL719 for 1 h followed by incubation with 500 μM 1938 for 1 h. Sitting drops were set up by mixing 100 nl of reservoir with 100 nl of protein solution (10 mg/ml) in 96-well MRC-plates. Initial crystals were obtained in 0.2 M KSCN, 0.1 M sodium cacodylate pH 6.5, and between 8-30% of PEG 2K, PEG 4K, PEG 5K and PEG 6K (w/v), or in 80 mM KSCN, 30% PEG 1K (w/v), 150 mM MES, pH 6.0. For optimisation, the crystallisation was set in a sparse matrix layout by varying the concentrations PEG and KSCN in hanging drops by mixing 1 μl of 5.5 mg/ml protein with 1 μl of reservoir. The best diffracting crystals were obtained in a condition containing 10% PEG 5K MME (w/v), 160 mM KSCN, 100 mM sodium cacodylate pH 6.5 for both the apo and PI3Kα/NVP-BYL719 structures. Crystals were also soaked between 1-20 h in 10 mM 1938. Before data collection, harvested crystals were immersed in a solution containing the precipitant mixture and 15% 2-methyl-2,4-pentanediol (MPD) and cryo-cooled in liquid nitrogen.

### Crystallisation of p110α-compound complexes

All crystallisation experiments were performed at 18°C. An initial screen of approximately 2300 conditions was performed using the LMB robotic crystallization setup^62^. p110α was either pre-incubated with 500 μM of 1938 or 1% DMSO for 1 h. Sitting drops were set up by mixing 100 nl of reservoir with 100 nl of protein solution (5.8 mg/ml) in 96-well MRC-plates. Crystals for apo were obtained from the Morpheus II screen, in 12.5% (w/v) PEG 4K, 20% (v/v) 1,2,6-hexanetriol, 40 mM Polyamines, 0.1 M MOPSO/bis-tris pH 6.5; and in 12.5% (w/v) PEG 4K, 20% (v/v) 1,2,6-hexanetriol, 90 mM LiNaK, 0.1 M MOPSO/bis-tris pH 6.5. For optimisation with 1938, crystallisation was set up in 96-well MRC-plates by varying the concentrations of PEG, 1,2,6-hexanetriol and polyamine or LiNaK in sitting drops by mixing either 200 nl of 5.8 mg/ml protein with 200 nl of reservoir, or 500 nl of 5.8 mg/ml protein with 500 nl of reservoir. Crystals only formed under apo conditions. Crystals were then soaked for 1.5-2 h in 20 mM 1938 (20% DMSO). For data collection, crystals for apo were obtained in conditions containing 12.5% (w/v) PEG 4K, 20% (v/v) 1,2,6-hexanetriol, 90 mM LiNaK, 0.1 M MOPSO/bis-tris pH 6.5 and crystals soaked with 1938 were obstained in conditions containing 12.5% (w/v) PEG 4K, 20% (v/v) 1,2,6-hexanetriol, 50 mM Polyamines, 0.1 M MOPSO/bis-tris pH 6.5. Harvested crystals were cryo-cooled in liquid nitrogen prior to data collection.

### Crystal data collection and refinement for p110α/p85α niSH2

All datasets were collected at 100 K. A crystal of the native PI3K was measured at the i03 beam-line (Diamond Light Source, UK), while the crystal of the PI3K/BYL719 structure was collected at the PetraIII P13 beam-line (EMBL-Hamburg/DESY, Germany)^63^. The native data set was indexed, processed and scaled using the XDS package^64^ and merged and scaled with AIMLESS^65^, while the PI3K/BYL719 was processed by XDS. Both crystals belonged to the P212121 space group with a solvent content 50.4 % corresponding to one complex (containing one catalytic and one regulatory subunit) in the asymmetric unit. The native PI3K structure was determined by molecular replacement using MOLREP^66^ and the PI3K structure with PDB ID 4JPS as a search model. The molecular replacement solution was then used as a starting model for refinement using the high-resolution native data-set of PI3K. After several iterations of rigid-body, maximum-likelihood and TLS refinement using the PHENIX suite^67^, manual building and model inspection using COOT^68^, the final model converged to a final Rwork/Rfree of 0.1964/0.2456 at a maximum resolution of 2.20 Å. The PI3K model covers the catalytic subunit residues 3-313, 322-501, 523-864 and 871-1065 and the regulatory subunit residues 326-591. This structure was used as a starting model for the PI3K/BYL719 structure which after refinement converged to a final Rwork/Rfree of 0.1873 / 0.2403 at a maximum resolution of 2.50 Å. Data collection and refinement statistics are summarised in Supplementary Table 2a.

### Crystal data collection and refinement for p110α 105-1048

X-ray diffraction for single crystals of p110α 105-1048 alone and soaked with 1938 were collected at the i04 and i24 beamlines, respectively (Diamond Light Source, UK). Images were processed using automated image processing with Xia2 (Ref.^69^). Both crystals belong to the P212121 space group. Initial phases were obtained with molecular replacement, using Phaser in the CCP4 suite, with an initial model from PDB entry 4TUU. Models were manually adjusted to the densities, using COOT^68^, and the structures were refined firstly with REFMAC^70^ and with PHENIX^67^ at later stages. A 3D model was built for 1938 from its chemical structure, using AceDRG in CCP4, and this model agreed well with the density in the 1938-soaked crystal. The final model converged to a final Rwork/Rfree of 0.24/0.28 at a maximum resolution of 2.41 Å for p110α-apo, and Rwork/Rfree of 0.21/0.27 at a maximum resolution of 2.57 Å for p110α-1938. Representations of the complex were prepared using PyMOL and Chimera. Data collection and refinement statistics are summarised in Supplementary Table 2b. Geometry of 1938 was checked using MOGUL 1.8.5 (Supplementary Table 9).

### Western blot analysis using enhanced chemiluminescence (ECL) detection

Unless otherwise indicated, western blotting was performed with ECL detection. For time course studies, A549 cells were seeded at 250,000 cells per well in 6-well plates in RPMI (10% FBS + 1 mM Na-Pyruvate + 1% P/S) and allowed to adhere overnight. The next day they were serum-starved for 4 h prior to treatment with 100 nM insulin or 5 μM 1938, for the indicated time (5 min to 48 h) at 37°C, 5% CO_2_. For 1938 titration assays in MEFs, cells were seeded at 150,000 cells per well in 6-well plates and allowed to adhere overnight. The next day they were serum-starved for 4 h prior to treatment with insulin (1 μM), 1938 (0.2 to 30 μM, final DMSO concentration of 1.5%) or inhibitors (final DMSO concentration of ≤1.5%) for 15 min at 37°C, 5% CO_2_. Cells were washed twice with cold PBS and lysed using cold RIPA buffer (25 mM Tris.HCl pH 7.6, 150 mM NaCl, 1% NP-40, 1% sodium deoxycholate, 0.1% SDS supplemented with protease/phosphatase inhibitor cocktail from Merck). To remove cell debris, homogenates were spun at 13,000 rpm for 15 min at 4°C and the supernatant fraction recovered. Protein concentration was determined by colorimetric assay (BCA assay, Promega). Protein extracts were resolved by SDS-PAGE, transferred to nitrocellulose membranes, and incubated overnight at 4°C with specific antibodies as follows: anti-vinculin (Sigma #V9131) and the following antibodies from Cell Signaling Technology (CST): pAKT-S473 (CST #9271), pS6-S240/244 (CST #2215), pPRAS40-T246 (CST #2640) and total S6 (CST #2317). Primary ntibodies were used at 1:1000 dilution except anti-vinculin (1:10000). Secondary antibodies are also from Cell Signalling Technology: Anti rabbit IgG, HRP linked Antibody (CST #7074S), Anti mouse IgG, HRP linked Antibody (CST #7076S). Raw and uncropped blots are shown in Supplementary Fig. 1.

### Western blot analysis using Wes™

A549 cells were seeded at 200,000 cells per well in 24-well plates in DMEM (10% FBS + 1% P/S) and allowed to adhere overnight. The next day, cells were washed once with PBS before addition of serum-free DMEM for 24 h. On the day of treatment, cells were incubated in fresh serum-free DMEM prior to treatment. 15 min pre-treatment with either PI3Kα inhibitor (BYL719, 500 nM) or 0.1% DMSO was performed prior to 1938 addition for 15 min at 37°C, 5% CO_2_. Following incubation, cells were washed and lysed in RIPA buffer (Thermo), supplemented with protease and phosphatase inhibitors (Roche). The lysate was collected and centrifuged at 15,000 rpm for 15 min at 4°C, supernatant collected and stored at -80°C. Western blotting was performed by Wes™ (ProteinSimple) according to the manufacturer’s instructions. Antibodies for pAKT-S473 (CST #4060) and total AKT (CST #9272) were used at 1:50; antibody to β-actin (CST #4970) was used at 1:100. Raw and uncropped blots are shown in Supplementary Fig. 1.

### Detection of AKT phosphorylation by ELISA

A549 cells were seeded at 50,000 cells per well in 96-well plates in DMEM (10% FBS + 1% P/S). The next day cells were washed once with PBS before addition of serum-free DMEM for 24 h. On the day of treatment, cells were incubated in fresh serum-free DMEM prior to treatment. Compounds solubilised to 10 mM in DMSO were diluted 1:3 in an 8-point concentration response curve in DMSO. Concentration response curves were diluted in serum-free DMEM by transfer into intermediate plates using a BRAVO liquid handler (Agilent). Intermediate plates were then used to treat cell plates using the BRAVO liquid handler. Compound concentration response curves had a top concentration of 50 μM and a final well concentration of 0.5% DMSO. Cell plates were treated for 15 min at 37°C, 5% CO_2_ before being washed with ice-cold PBS and lysed in lysis buffer 6 (R&D Systems #895561) and freezing at -80°C. Levels of pAKT^S473^ were determined using the phospho-AKT (S473) pan-specific Duoset IC ELISA (R&D Systems #DYC887BE) in 96-well white high-binding plates (Corning #3922) according to manufacturer’s instructions. Endpoint luminescence was measured using a Sense (Hidex) platereader. Compound data were corrected to the negative DMSO control and expressed as a percentage the internal insulin control (1 μM), equivalent to the maximal activation (Emax) induced by insulin. Data were transformed and EC_50_ data were determined by variable slope (4 parameters) non-linear regression using Prism 7 (Graphpad).

### Cell culture

Immortalised PI3Kα-WT and PI3Kα-KO MEFs were generated and described previously^45^. MEFs were cultured in DMEM containing 10% FBS and 1% penicillin-streptomycin and starved in serum-free DMEM with 1% penicillin-streptomycin at 37°C and 5% CO_2_. A549 cells were cultured either in DMEM Glutamax (Gibco #31966021) supplemented with 10% FBS and 1% penicillin-streptomycin, or in RPMI 1640 medium supplemented with 10% FBS, 1 mM sodium pyruvate and 1% penicillin-streptomycin. For starvation experiments, A549 cells were incubated in serum-free RPMI containing 1 mM sodium pyruvate and 1% penicillin-streptomycin. All cell cultures were regularly tested to be negative for Mycoplasma.

### Plasmid vectors

Flag-*PIK3R1* (a kind gift of Neil Vasan), *PIK3CA*-WT and mutant *PIK3CA* (D603K, L1006R, F1016S and L1006R/F1016S) were cloned into pcDNA3.4TOPO. pcDNA3-eGFP was used as a control for transfection efficiency and is available on Addgene repository upon request (Plasmid #13031). The mutations in *PIK3CA* were introduced by site-directed mutagenesis according to published commercial protocols using NEBuilder HiFi DNA Assembly Master Mix (NEB E2621). For each mutation, a pair of primers were designed to incorporate a mutation within the gene, with the resulting PCR products containing a 20 bp overlap and the desired mutations. The DpnI-treated and purified PCR products were combined with the linearized vector and treated with the NEBuilder HiFi DNA Assembly Master Mix. Following transformation, single colonies were grown and purified with QIAprep spin Miniprep kit (Qiagen 27106). All plasmids were sequenced for verification.

### Transient transfection of WT and mutant *PIK3CA* constructs in MEFs

eGFP, Flag-*PIK3R1*, *PIK3CA*-WT and mutant *PIK3CA* (D603K, L1006R, F1016S, L1006R/F1016S) plasmids were used for transient transfection. *Pik3ca*-KO MEFs cells were seeded at 70-80% confluency in 6-well plate at 100,000 cells per well. The next day, fibroblasts was transfected using Lipofectamine™ 3000 Transfection Reagent (ThermoFisher) as follows: 2.5 μg of plasmid DNA cocktail (containing equimolar amount 1:1 of *PIK3CA:PIK3R1* plasmids) were mixed in Opti-MEM medium (ThermoFisher) with 5 μl P3000 reagent. The diluted DNA mix was then added to a premix containing 7.5 μl Lipofectamine 3000 in Opti-MEM in 1:1 ratio. After 10 min incubation, the DNA-lipid complex was added to the cells and incubated for 48 h before treatment. All cells were serum starved for 4 h before treatment with 5 μM of 1938 or DMSO and lysed in RIPA buffer.

### Generation of *PIK3CA*-null A549 cells by CRISPR/Cas9 gene targeting

Generation of pooled *PIK3CA*-null A549 cells was outsourced to Synthego Corporation. Briefly, the *PIK3CA* gene was targeted with synthetic ribonucleoprotein (RNP) complexes including the following single guide RNA (sgRNA) sequence: *5’-CUCUACUAUGAGGUGAAUUG-3’*. This sequence is located within *PIK3CA* exon 3 and covers the coding sequence preceding the p110α RAS binding domain, with the Cas9 cut site corresponding to amino acids 156/157 of p110α). In parallel, control cultures were exposed to the Cas9 protein without sgRNA, henceforth referred to as “WT cultures”. Single-cell clones were established from both WT and targeted cultures by limiting dilution, thereby ensuring seeding of maximum 1 cell per well of a 96-well plate. To promote recovery, subcloned cells in 96-wells were cultured in a 1:1 mixture of standard A549 complete medium and conditioned medium. Conditioned medium was prepared from WT cultures 2 days post-passaging by centrifuging the medium at 1000g for 10 min, followed by 0.22 μm PES filtration and storage at 4°C (-80°C for storage exceeding 2 weeks). The medium was replenished every 2-3 days, as gently as possible to prevent cells from dislodging. Once cells reached sub-confluence, they were expanded to 24-well plates and 25 cm^2^ flasks, followed by genotyping and cell banking.

For genotyping, genomic DNA was extracted from replicas of the cells cultured in 24-well plates using 50 μl QuickExtract solution (Cambridge Bioscience #QE0905T) and the following thermocycling conditions: 68°C for 15 min, 95°C for 10 min, 4°C HOLD. The edited locus was amplified by standard PCR using GoTAQ G2 MasterMix (2X) (Promega #M7822) with 2 μl QuickExtract-processed genomic DNA and the following primers: F 5’-*TCTACAGAGTTCCCTGTTTGC*-3’; R 5’-*AGCACTCAACTATATCTTGTCAGT*-3’. Annealing and extension wwere performed at 55°C for 30 sec and 72°C for 30 sec, respectively. The PCR reactions were cleaned up with ExoSAP-IT Express (Thermo Fisher Scientific #75001.1.ML) according to the manufacturer’s instructions, at 37°C for 30 min followed by 80°C for 1 min. The cleaned-up reactions were submitted for Sanger sequencing (Eurofins Genomics). Subsequent analyses of the Sanger sequencing traces were performed using Synthego’s open-source ICE tool^71^. Next, all predicted knock-out (KO) clones were validated by Western blotting for the *PIK3CA* protein using two complementary antibodies (CST #4249 and CST #4255; each used at 1:1000 dilution in 1X TBS/T with 3% BSA). Clones with confirmed loss of p110α expression were kept for further experimental studies.

**Extended Data Fig. 10:**
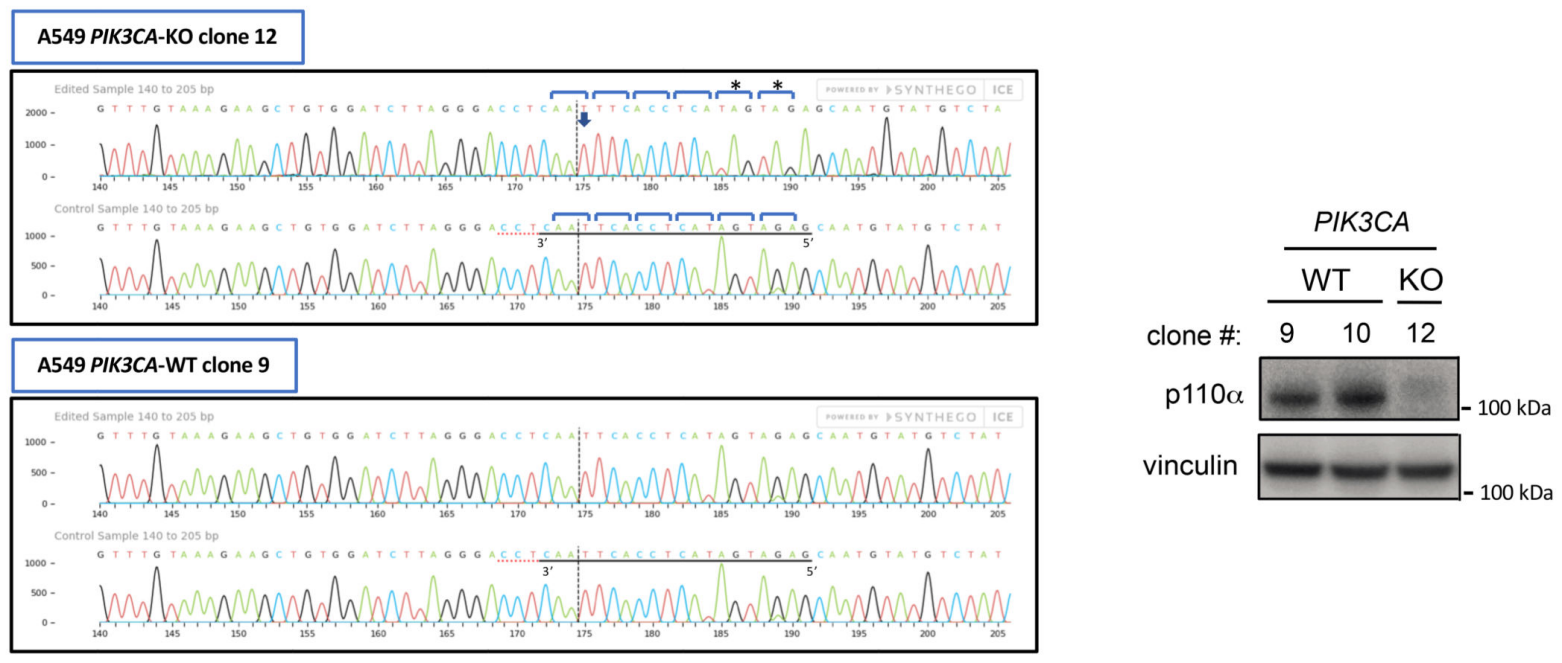
Additional data for methodology. *Left panel*, Sanger sequencing of the genomic *PIK3CA* locus of A549 cell clones subjected to CRISPR/Cas9 gene-targeting. *Lower traces:* reference genomic *PIK3CA* sequence (wild-type), with the crispr RNA sequence underlined. *Top traces:* DNA sequence of CRISPR/Cas9 gene-targeted or control-edited A549 clones. The *PIK3CA*-KO clone 12 shows a +1 bp insertion (arrow), leading to frameshift and the generation of 2 consecutive premature stop-codons (asterisk) immediately downstream of the +1 bp insertion. Note that the first stop-codon occurs 80 bp upstream from the 3’ exon-exon junction and will therefore result in nonsense-mediated decay of the mRNA. The *PIK3CA*-WT clone 9 shows wild-type genomic DNA sequence. *Right panel*, Western blot for p110α using antibody CST#4255.

The A549 clones used for the TIRF experiments (Fig. 3d) were *PIK3CA-WT* clone 9 and *PIK3CA-KO* clone 12. The DNA sequencing traces and p110α Western blots of these A549 clones are shown in Extended Data Fig. 10. The *PIK3CA-KO* clone 12 shows a +1 bp insertion, resulting in a frameshift and the generation of a premature stop codon as shown in Extended Data Fig. 10 *left panel*.

### Mass spectrometry-based phosphoproteomics

PI3Kα-WT and PI3Kα-KO MEFs, grown in 15 cm dishes, were serum-starved overnight in DMEM with 1% penicillin-streptomycin and stimulated by the addition of 0.05% DMSO, 5 μM 1938 in final 0.05% DMSO or 100 nM insulin (Sigma #I5016) for 15 min or 4 h. Cells were lysed in 500 μl urea lysis buffer [50 mM triethylammonium bicarbonate, 8 M urea, cOmplete™ EDTA-free protease inhibitor cocktail (1:50 dilution) (Roche #11873580001), 1 PhosSTOP tablet (Roche #4906845001) per 10 ml of lysis buffer, 1 mM sodium orthovanadate] and lysates sonicated until clear for ~10 min with cooling breaks on ice. Protein concentration was measured using a BCA protein assay (Pierce #23227). 300 μg of protein was reduced with 5 mM Tris(2-carboxyethyl)phosphine hydrochloride (Sigma #C4706) at 37°C for 20 min and alkylated using 10 mM 2-chloroacetamide (Sigma #22790) for 20 min at room temperature in the dark. Proteins were digested with LysC (#129-02541; FUJIFILM Wako Chemicals, Osaka, Japan) for 3.5 h at 30°C. Samples were then diluted with 50 mM triethylammonium bicarbonate (Sigma #T7408) to reduce the urea concentration to 1.5 M, followed by an overnight peptide digestion with trypsin (Promega #V5113) at 37°C. Digest reactions were quenched by the addition of 10% trifluoroacetic acid (EMD Millipore #302031-M) to a final pH of 2.0. Sample desalting was performed using 35-350 μg C18 columns (HMM S18V; The Nest Group, Inc., Southborough, MA, USA) according to the manufacturer’ specifications. TiO_2_ (Hichrome Titansphere TiO_2_, 10 μm capacity, 100 mg, GL Sciences #5020-75010) was used for phosphoenrichment. Following peptide loading onto TiO_2_, the beads were sequentially washed with 1 M glycolic acid (Sigma #124737)/80% acetonitrile/5% trifluoroacetic acid, followed by 80% acetonitrile/0.2% trifluoroacetic acid and 20% acetonitrile before elution with 5% ammonium hydroxide. Enriched samples were desalted using 7-70 μg C18 columns (HUM S18V; The Nest Group, Inc., Southborough, MA, USA) according to the manufacturer’s specifications. Dried phosphopeptide samples were stored at -80°C and resuspended in 10% formic acid immediately prior to analysis. nLC-MS/MS was performed on a Q-Exactive Orbitrap Plus interfaced to a NANOSPRAY FLEX ion source and coupled to an Easy-nLC 1000 (Thermo Scientific). Fifty percent of each sample was analysed as 10 μl injections. Peptides were separated on a 27 cm fused silica emitter, 75 μm diameter, packed in-house with Reprosil-Pur 200 C18-AQ, 2.4 μm resin (Dr. Maisch, Ammerbuch-Entringen, Germany) using a linear gradient from 5% to 30% acetonitrile/0.1% formic acid over 180 min, at a flow rate of 250 nl/min. Peptides were ionised by electrospray ionisation using 1.9 kV applied immediately prior to the analytical column via a microtee built into the nanospray source with the ion transfer tube heated to 320°C and the S-lens set to 60%. Precursor ions were measured in a data-dependent mode in the orbitrap analyser at a resolution of 70,000 and a target value of 3e6 ions. The ten most intense ions from each MS1 scan were isolated, fragmented in the HCD cell, and measured in the Orbitrap at a resolution of 17,500.

### Peptide identification, quantification and statistical analysis of phosphoproteomics data

Raw data were analysed with MaxQuant^72^ (version 1.5.5.1) where they were searched against the mouse UniProt database (http://www.uniprot.org/, downloaded 04/12/2018) using default settings. Carbamidomethylation of cysteines was set as fixed modification, and oxidation of methionines, acetylation at protein N-termini, phosphorylation (on S, T or Y) were set as variable modifications. Enzyme specificity was set to trypsin with maximally 2 missed cleavages allowed. To ensure high confidence identifications, peptide-spectral matches, peptides, and proteins were filtered at a less than 1% false discovery rate (FDR). Label-free quantification in MaxQuant was used with a LFQ minimum ratio count of 2, Fast LFQ selected and the ‘skip normalisation’ option selected. The ‘match between runs’ feature was selected with a match time window of 0.7 min and an alignment time window of 20 min. The MaxQuant ‘phospho(STY)Sites.txt’ output file was reformatted by merging each protein accession and gene name with its corresponding phosphosite to obtain an ‘Annotated_PhosphoSite.txt’. This file, together with the MaxQuant ‘evidence.txt’ output file and an experimental design ‘annotation.csv’ file, was further processed by removing contaminants and reversed sequences, and the removal of phosphosites with 0 or 1 valid values across all runs. High experimental reproducibility was observed, as evidenced by an average Pearson Correlation Coefficient of r=0.862 for biological replicates (Extended Data Fig. 7e). Quantified phosphopeptides were analysed within the model-based statistical framework MSstats^73^ (version 3.20.0, run through RStudio (version 1.2.5042, R version 4.0.0)). Data were log2 transformed, quantile normalised, and a linear mixed-effects model was fitted to the data. The group comparison function was employed to test for differential abundance between conditions. p-values were adjusted to control the FDR using the Benjamini-Hochberg procedure^74^. The mass spectrometry proteomics data have been deposited to the ProteomeXchange Consortium via the PRIDE^75^ partner repository with the dataset identifier PXD027993. Reviewer account details: Username: reviewer_pxd027993@ebi.ac.uk; Password: FSaiKH6M)

### Quantification of phosphoinositide species by mass spectrometry

A549 cells (5.10^5^) or wild-type MEFs (3.10^5^) were plated in complete media onto 3.5 cm dishes or 60 mm dishes respectively for 24 h, prior to FCS-free starvation media for 16 h. MEFs, in addition, were supplemented with arachidonic acid in the FCS-free starvation media as previously described^76^. Cells were stimulated with indicated doses of 1938, PDGF-BB, insulin, or DMSO vehicle control (corresponding to DMSO amounts in 30 μM 1938) for the indicated time points at 37°C, 5% CO_2_. Reactions were terminated in 600 μl ice-cold 1 M HCl and cells scraped and resuspended, then divided into two equal samples in eppendorfs. Samples were processed and extracted for C38:4:PI(3,4,5)P3, or PIP_2_ regio-isomer C38:4-PI(3,4)P_2_/PI(4,5)P2 analysis by mass spectrometry, essentially as described^39^, with the exception that the following internal standards (ISDs, all synthesized by the Biological Chemistry Department at the Babraham Institute, Cambridge) were also included: d6-stearoyl-arachidonoyl (C18:0/C20:4) -PI (74.8 ng), -PI(4)P (925 ng) and -PI(4,5)2/PI(3,4)P_2_ (prepared as a 1:1 mix, 100 ng total PIP_2_). The data are shown as response ratios, calculated by normalizing the multiple reaction monitoring (MRM)-targeted lipid integrated response area to that of a known amount of relevant internal standard. To account for any cell input variability, PIP_3_ response ratios were normalized to C38:4-PI response ratios; while PI(3,4)P_2_ response ratios were normalised to PI(4,5)P2 response ratios. Data shown are mean ±SD for n ≥3, except for non-DMSO control in MEF PIP_3_ measurements where n=1.

### Total internal fluorescence (TIRF) microscopy of phosphoinositide reporters

Phosphoinositide reporters used were GFP-PH-ARNO^I303E^x2 (PIP_3_ reporter^41^) and mCherry-cPH-TAPP1x3 (PI(3,4)P_2_ reporter^41^). TIRF microscopy allows selective imaging of the small cell volume, including the plasma membrane, directly adjacent to the coverslip onto which cells have been seeded. HeLa or A549 cells were seeded in Matrigel-coated (Corning #354230; diluted in Opti-MEM at 1:50) 8-well chamber slides (glass bottom, 1.55 refractive index; Thermo Fisher Scientific #155409) at a density of 5,000 cells/well. The following day, cells were transfected with 50 ng (A549) or 10 ng (HeLa) PIP_3_ reporter plasmid (GFP-PH-ARNO^I303E^x2)^41^ using FuGENE® HD Transfection Reagent (Promega #E2311), at a 3:1 Fugene:DNA ratio according to the manufacturer’s instructions. To ensure low yet uniform expression of the reporters in HeLa cells, and to aid in the identification of the critical TIRF angle for imaging, these cells were also co-transfected with 200 ng iRFP-tagged Paxillin plasmid (generated by conventional restriction enzyme-based subcloning from an mCherry-Paxillin plasmid, Addgene #50526). In separate experiments, HeLa cells were also transfected with 10 ng or 50 ng of the PI(3,4)P_2_ reporter mCherry-cPH-TAPP1x3 (Ref.^41^); the use of 50 ng of this reporter enabled easier visualisation in the TIRF field, however the kinetics of the response remained unchanged and results from both experiments were pooled.

Following another 24 h post-transfection, cells were switched to 150 μl serum-free Fluorobrite™ DMEM (Thermo Fisher Scientific #A1896701; supplemented with L-glutamine (2 mM) and 1% penicillin-streptomycin for 3 h prior to time-lapse imaging on a 3i Spinning Disk Confocal microscope fitted with a sCMOS Prime95B (Photometric) sensor for TIRF, with full temperature (37°C) and CO_2_ (5%) control throughout the acquisitions. A 100X 1.45 NA plan-apochromatic oil-immersion TIRF objective was used to deliver the laser illumination beam (40-50% power) at the critical angle for TIRF and for acquisition of the images by epifluorescence (300-500 msec exposure) using single bandpass filters (445/20 nm and 525/30 nm). Acquisition was performed in sequential mode, without binning, using Slidebook 6.0 and an acquisition rate of 2 or 3 min as indicated. Individual treatments were added at the specified times at 2x to 5x concentration in the same imaging medium, ensuring correct final concentration and sufficient mixing with the existing medium solution. BYL719 (Advanced ChemBlocks Inc #R16000) was used at a concentration of 0.5 μM.

Image analyses of total reporter intensities were performed with the Fiji open source image analysis package^77^. The region of interest (ROI) corresponding to the footprint of the individual cell across time points were defined using minimal intensity projection to select only pixels present across all time points, following prior subtraction of camera noise (rolling ball method, radius = 500 pixels) and xy drift correction, intensity levels over time were measured. These analyses were performed with a custom-written FIJI/ImageJ macro. A second macro was used to generate scaled images, with normalisation of all pixels to pre-treatment average intensity (F_t_/F_baseline_). All other quantifications were performed using the open source software R/RStudio. The values plotted in Fig. 3d (iii) represent mean±SEM, following signal scaling to minimum and maximum values of the normalised fluorescence intensity for each time point (Fn_(t_)). All raw images, macros and R analysis scripts are provided via the Open Science Framework (https://osf.io/gzxfm).

### CellTiter-Glo® cell assay

MEFs were seeded at 5000 cells per well in 96-well plates in DMEM supplemented with 10% FBS and 1% P/S, and allowed to attach overnight. The next day, cells were serum starved for 4 h prior to compound treatment in fresh serum-free DMEM. Compounds solubilised in DMSO were diluted 1:2 in a 12-point concentration response curve in DMSO. Intermediate plates were prepared by transferring 4 μl of compounds in DMSO into 96 μl of serum-free DMEM media. This was then used to treat cell plates by transferring 12.5 μl of solution from the intermediate plate into 87.5 μl of serum-free DMEM in the cell plates. Compound concentration response curves had a top concentration of 30 μM and a final well concentration of 0.5% DMSO. Cell plates were incubated for 24 h, 48 h or 72 h at 37°C, 5% CO_2_, followed by determination of cell survival using the CellTiter-Glo® reagent according to manufacturer’s instructions (Promega #G7571). Endpoint luminescence was measured using CLARIOstar (BMG). Compound data were analyzed using GraphPad Prism 8.

### Measurement of cell proliferation by crystal violet staining

MEFs were seeded at 5000 cells per well in 96-well plates in DMEM supplemented with 10% FBS and 1% P/S, and allowed to attach overnight. The next day, cells were serum-starved for 5 h prior to compound addition in fresh serum-free DMEM. After different time points, cells were rapidly washed with distilled H2O before fixed and stained in a solution of 0.5% crystal violet (Sigma-Aldrich #C0775) in 20% methanol (v:v) as described^78^. Briefly, after 20 min incubation at room temperature on a rocking platform, fixed and stained cells were washed 3 times with distilled H2O and plates air-dried overnight. 200 μl methanol was next added to each well and the plates were incubated at room temperature for 20 min on a bench rocker, followed by measurement of optical density at 570 with a plate reader.

### Measurement of cell cycle progression by EdU staining

The Click-IT EdU protocol was used according to manufacturer instructions (Sigma-Aldrich #BCK-FC488-50). Briefly, MEFs were seeded at 50,000 cells per well in 6-well plates in DMEM supplemented with 10% FBS and 1% P/S, and allowed to attach overnight. The next day, cells were serum-starved for 5 h prior to compound addition for 24 h in fresh serum-free DMEM. Cells were then pulsed for 3 h with 10 μM EdU, followed by collection by trypsinization and fixation with 3.7% FA in PBS for 15 min in the dark, washed in 3% BSA and permeabilized in 1x saponin-based permeabilization buffer for 20 min in the dark. EdU was then detected using the FAM-azide assay cocktail for 30 min in the dark. Cells were washed twice in 1x saponin-based permeabilization buffer followed by analysis with flow cytometer (Novocyte Advanteon flow cytometer, Agilent). Gating strategy for flow cytometry is shown in Supplementary Fig. 2.

### Langendorff perfused heart preparation in rats

The Langendorff *ex vivo* perfused rat heart was used as an experimental model of IRI^79^. The animal experiments were conducted within the terms of the UK Animals (Scientific Procedures) Act 1986, under Project Licence number PPL 70/8556 (Protection of the Ischaemic and Reperfused Myocardium). All procedures conform to the guidelines from Directive 2010/63/EU of the European Parliament on the protection of animals used for scientific purposes. Male Sprague–Dawley rats were bred at a central animal unit in University College London and used at a weight of 250–350 g, and randomly assigned to experimental groups. Rats were anaesthetised by intraperitoneal injection of sodium pentobarbitone (60 mg/kg) (Animalcare, York, UK). Hearts were quickly excised via a clamshell thoracotomy and the aorta cannulated and retrogradely perfused on a Langendorff apparatus with a modified Krebs–Henseleit buffer (118 mM NaCl, 25 mM NaHCO_3_, 11 mM D-glucose, 4.7 mM KCl, 1.22 mM MgSO_4_.7H_2_O, 1.21 mM KH_2_PO_4_ and 1.84 mM CaCl_2_.2H_2_O, 37°C, pH 7.35–7.45, gassed with 95% O_2_/5% CO_2_) with a gravity-fed perfusion pressure of 70–80 mm Hg according to standard methods^79^. The temperature of the heart was maintained at 37.0 ± 0.5°C. All hearts were made globally ischaemic by stopping flow for 45 min and then reperfused for 2 h. The heart was perfused during the first 15 min of reperfusion with modified Krebs–Henseleit buffer containing either 1938 (5 μM) or insulin (1 μM) or an equivalent volume of vehicle (DMSO, final concentration 0.1 %). At the end of the protocol, hearts were frozen at -20°C before being sectioned into 5 transverse slices and stained for viable tissue by immersion in 1% triphenyl-tetrazolium chloride at 37°C for 15 min. Following fixation in 10% formalin for 24 h, the sections were digitally scanned for analysis. Analysis of infarct size (IS) as a proportion of area at risk (AAR) was calculated via planimetry using imageJ software (version 1.45, National Institutes of Health, USA).

Alternatively, after 15 min reperfusion, perfusion was stopped and hearts were freeze-clamped in liquid nitrogen and frozen at -80°C. Tissues were incubated in lysis buffer (100 mM Tris.HCl pH 7.4, 300 mM NaCl, 0.5% IGEPAL with 1x Halt protease inhibitor cocktail (#78429; Thermo Scientific, Loughborough, UK), 1x Halt phosphatase inhibitor cocktail (#78420; Thermo Scientific, Loughborough, UK) and 5 μM EDTA (Thermo Scientific, Loughborough, UK)) and homogenised on ice using a Potter-Elvehjem tissue grinder for 1 min using 20 strokes with the pestle, and sonicated on ice (3-5 pulses of 5 sec, amplitude 40-50 x 25) using a Vibracell sonicator. Protein content was determined by bicinchoninic acid (BCA) assay (Sigma-Aldrich, Gillingham, UK). Tissue lysates were mixed with NuPAGE LDS Sample Buffer (Thermo Fisher Scientific) plus 2.5% 2-mercaptoethanol and denatured at 80°C for 10 min. 20 μg protein was run on NuPAGE Novex 10% Bis-Tris protein gels (Thermo Fisher Scientific, Loughborough, UK) using the Mini Protean III system (Bio-Rad, Watford, UK) and electro-transferred onto nitrocellulose blotting membrane (GE Healthcare Life Science, Amersham UK) using wet transfer in a Bio-Rad Mini Trans-Blot. The membranes were blocked in 5% bovine serum albumin/TBS-Tween-20 (#P2287; Sigma; 0.1%) then incubated with primary antibodies at 4°C overnight. Primary antibodies used were directed against total AKT (#2920; Cell Signaling Technology, UK), pAKT^S473^ (#4060; Cell Signaling Technology, UK) and β-actin (#sc-47778; Santa Cruz Biotechnology, UK) as a gel loading control. The next day, membranes were probed with IRDye fluorescence-tagged secondary antibodies (#926-32211 and #926-68020; LI-COR Biosciences, Ltd. UK) and imaged and quantified using the Odyssey imaging system (Image Studio Lite Ver 5.2; LI-COR Biosciences, Cambridge, UK).

### *In vivo* model of ischaemia reperfusion injury in mice

Male C57/BL6 mice weighing 25-30 g were used throughout. Animals received humane care in accordance with the United Kingdom Home Office Guide on the Operation of Animal (Scientific Procedures) Act 1986, Project Licence PPL70/15358.

Animals were anaesthetised with intraperitoneal (i.p.) sodium pentobarbital at a dose of 100 mg/kg. The mice were intubated by tracheotomy and ventilated with room air using a small animal ventilator (MinVent, Type 845, Hugo Sachs Elektronik, Harvard Apparatus). The mice were then placed on a heating pad and the rectal temperature monitored and maintained at ~37°C using a temperature controller. During the experiments, both ECG and heart rate were continuously recorded using a PowerLab (Adinstrument, USA). The chest was opened in the intercostal space between the 3^rd^ and 4^th^ ribs to expose the heart, and a suture was placed around the left anterior descending (LAD) coronary artery followed by a snare to allow the occlusion and opening of the LAD. The left external jugular vein was canulated for drug administration.

By tightening the suture snare to occlude the LAD coronary artery, the hearts were subjected to 40 min ischaemia, which was confirmed by both ST-segment elevation on the ECG and a change in heart colour. After 40 min, the snare was loosened and the heart allowed to reperfuse for the next 120 min. 15 min prior to reperfusion, 50 μl of DMSO vehicle or 10 mg/kg 1938 compound in DMSO, was slowly injected via the jugular vein. The person carrying out the experiment was blinded to the treatment groups.

After 120 min reperfusion, the chest was re-opened, the heart was removed and canulated via the thoracic aorta, and blood within the heart was washed out with saline. The LAD coronary artery was then re-occluded with the suture that had been left loosely in place following ischaemia, and the hearts were injected with 2% Evans blue to delineate the area at risk. These hearts were then frozen at -80°C for ~10 min and subsequently cut into 5-6 slices of ~0.5 mm thickness. The heart slices were incubated in triphenyltetrazolium chloride (10 mg/ml) solution at 37°C, pH 7.4 for ~15 min to delineate viable (stained red) from the necrotic tissue (white regions). Slices were then transferred to 10% formalin solution and fixed overnight. The heart slices without right ventricular wall were then scanned using a Cannon digital scanner. The total area of myocardium, the non-ischaemic area (which is stained with Evans blue), and the infarct area (i.e. the white area) of each slice were measured using Image-J software. The “area at risk” was calculated by subtraction of the non-ischaemic area (blue area) from the whole slice area and expressed as “percentage of the left ventricle”, and “infarct size” calculated as infarct area as a percentage of the area at risk. 4 mice died during the experiment, before reperfusion (3 in DMSO group, 1 in 1938 group) and were excluded from analysis.

Analysis of tissue samples by Western blotting was performed as follows. 50 μl of DMSO vehicle or 10 mg/kg 1938 compound in DMSO, was injected via the jugular vein of anaesthetized and intubated mice as described above. After 15 min, the chest was opened, and the heart removed and freeze-clamped in liquid nitrogen. Hearts were then homogenized in lysis buffer [100 mM Tris.HCl, 300 mM NaCl, 1% IGEPAL, pH 7.4 supplemented with protease inhibitors (78438; Thermo Fisher Scientific) and phosphatase inhibitors (78427; Thermo Fisher Scientific)], by disruption using a pestle and mortar and sonicated on ice 5 times for 3 sec. The supernatant was then collected and after the addition of NuPAGE™ LDS Sample Buffer (4X) (Thermo Fisher Scientific), samples were boiled and stored at -80°C until SDS-polyacrylamide gel electrophoresis (SDS-PAGE) was performed. 20 μg of protein per well was loaded on a 10% NuPAGE Bis-Tris gel (Invitrogen), resolved by SDS-PAGE, and transferred to PVDF membranes (Millipore) for Western blot analysis. Membranes were incubated with primary antibodies in 5% BSA/TBS-0.1% Tween-20 overnight at 4°C, washed three times for 10 min with TBS-0.1% Tween then incubated with secondary antibodies in 5% BSA/TBS-0.1% Tween for 1 h, followed by washing three times for 10 min with TBS-0.1% Tween. Antibodies used were mouse monoclonal antibody to β-actin (Santa Cruz; sc-47778; used at 1:2000), mouse monoclonal antibody to total Akt (Cell Signaling Technology; CST2920; used at 1:1000) and rabbit antibodies from Cell Signaling Technology to phospho-Akt Ser473 (CST9271; used at 1:1000). Secondary antibodies used were IRDye 680LT goat anti-mouse and IRDye 800CW goat anti-rabbit (LI-COR Biosciences). Proteins were visualized and quantified using the Odyssey Imaging System (LI-COR Biosciences).

### Animals for neurological studies

Adult rats (Charles River, UK) were housed in groups of 4-5 per cage and maintained on a 14:10-h light/dark cycle with ad lib access to food and water. All experiments were conducted in accordance with the UK Animals (Scientific Procedures) Act (1986) and the European Communities Council Directives (86/609/EEC), with approval from the University College London Animal Welfare and Ethical Review Board. Rats of the same sex and age were randomly assigned to experimental groups, and the experimentor and the analyst double-blinded.

### Quantification of neurite outgrowth

Dissociated adult rat dorsal root ganglion (DRG) cultures were used as an *in vitro* model for neuroregeneration^80,81^. DRG neurons were isolated from adult male (>250g) Wistar rats as described, with DRGs from each rat cultured separately^81^. Following culling via schedule 1 (rising concentration of CO_2_), the spinal column was removed and stored in PBS on ice. Cord tissue was removed to expose the DRGs and roots in the intervertebral foramen and the DRGs removed with forceps and scalpel under a dissecting microscope (Olympus SZ40). DRGs were manually cleaned by removal of roots, capsule and capillaries with forceps and then placed in DMEM supplemented with P/S. DRGs were treated with 0.125% collagenase type IV solution at 37°C for 90 min, and then mechanically dissociated by trituration using a 1 ml pipette. The collagenase solution was removed by 2 rounds of centrifugation in complete DMEM (DMEM with 1% P/S and 10% FBS) at 400 xg for 5 min, followed by resuspension of the DRG cell pellet in complete DMEM supplemented with 0.01 mM cytosine arabinoside. DRGs were plated in 75-cm^2^ flasks coated with 0.1 mg/ml poly-D-lysine and incubated at 37°C, 5% CO_2_. 24 h later, DRGs were resuspended by trypsinisation, and the trypsin was removed by centrifugation at 190 xg for 4 min. The resultant cell pellet was resuspended by mechanical trituration in Neurobasal-A medium (Gibco #10888022) supplemented with B-27 (Gibco #17504044), 2 mM L-Glutamine (Merck #G7513) and 1% penicillin/streptomycin. DRGs were plated onto 0.1 mg/ml poly-D-lysine-coated clear bottom black-walled 384-well plates (Greiner #781090) at a density of 1,000 cells/well. Cells were incubated at 37°C, 5% CO_2_ for 24 h. Prior to treatment, cells were washed with supplemented Neurobasal-A medium using a BRAVO liquid handler (Agilent) to a uniform volume. 1938 solubilised at 3 mM in DMSO was diluted 1:3 in an 8-point concentration response curve in DMSO. Drugs in concentration response curves were diluted in supplemented Neurobasal-A medium by transfer into intermediate plates using a BRAVO liquid handler. Intermediate plates were then used to treat cell plates using the BRAVO liquid handler (final concentration of 0.1% DMSO in the DRG cultures). The PI3Kα inhibitor BYL-719 (final concentration of 500 nM in the DRG cultures) or vehicle (0.005% DMSO in supplemented Neurobasal-A medium) was added 15 min prior to the addition of the 1938 concentration response curve (total concentration of 0.105% DMSO in the DRG cultures). After incubation for 72 h at 37°C and 5% CO_2_, cells were fixed by addition of 4% paraformaldehyde for 20 min. Wells were washed 3 times in PBS with 0.05% Tween-20 (PBST) before permeabilisation in PBS with 0.1% Triton X-100. Wells were washed 3 more times with PBST before blocking with fish skin gelatin/PBST for 1 h at room temperature. The wells were then incubated overnight at 4°C with primary antibody against the β-III tubulin neuronal marker; abcam #ab18207; 1:1000). The following day, cells were washed 3 times in PBST using the BRAVO liquid handler before incubation with anti-rabbit Alexafluor-488 (1:2000, A-11008) for 1 h at room temperature. Cells were washed 3 times with PBST using the BRAVO liquid handler before staining with Hoechst 33342 nucleic acid stain (Thermo Scientific #62249; 1:2000) for 20 min protected from light. Cells were washed another 3 times with PBST and 3 times with PBS and cell plates stored at 4°C protected from light before imaging. Image acquisition was performed using Opera (PerkinElmer) high-content screening system using the 20x water objective. Images of cell nuclei and β-III tubulin-positive cells were captured using excitation/emission wavelengths λ380/455 and λ490/518, respectively. 9 fields per well were captured and analysed using the CSIRO Neurite Analysis 2 logarithm in Columbus analysis software (Perkin Elmer). Neurites were defined using the following parameters: Smoothing window 0 pixels (px), Linear window 15 px, Contrast > 1.5, Diameter ≥ 3 px, Gap closure distance ≤ 17 px, Gap closure quality 0, Debarb length ≤ 40 px, Body thickening 1 px, Tree length ≤ 0 px. Within each experiment treatments were performed in quadruplicate and data are represented as the average of biological repeats (n= 3) ± standard error of the mean. Variable slope nonlinear regression (4 parameters) was performed in Prism 7. Whole well representative images were captured using Cytation 3 (Biotek) imaging plate reader using a 10X objective. A montage of images was captured before stitching and deconvolution in Gen 5 software (Biotek). Images of cell nuclei and β-III tubulin-positive cells were captured using excitation/emission wavelengths λ380/455 and λ490/518, respectively.

### Control experiments for nerve crush assays

Experiments to test the stability of 1938 in aqueous solution and the biological activity of 1938 on exposed rat sciatic nerves were performed as follows.

Lyophilised 1938 was solubilised in autoclaved dH2O to 100 μM. Solubilisation required sonication at 30°C for 25 min before passing through a 0.22 μm filter. Aliquots of 1938 (at 5 μM and 100 μM) or vehicle were frozen at -20°C in aliquots for later use on separate experimental days. An aliquot of 100 μM TRO-1938 and vehicle was defrosted and tested on A549 cells to test activity (Extended Data Fig. 9a, top panel). Cells were seeded in 24-well plates at 200,000 cells/well in DMEM+Glutamax supplemented with 10% FBS and 1% Pen/Strep. Prior to treatment, cells were washed and incubated with serum-free DMEM+Glutamax. Cells were treated with an 8 point 1:3 dose response of 1938 diluted in serum-free DMEM+Glutamax starting from 10 μM for 15 min at 37°C. Cells were then washed in ice-cold PBS and lysed in RIPA buffer supplemented with protease and phosphatase inhbitors. Lysates were analysed by automated Western blot (Wes) (data shown in Extended Data Fig. 9a; top panel).

To assess if 1938 could induce pAkt generation in exposed rat sciatic nerves, adult male Sprague Dawley rats (>250g; n=2) were anaesthetised using isoflurane, the left sciatic nerve was exposed and injected with 2 μl vehicle (sterile dH2O) or 1938 (5 μM in sterile dH2O). Meanwhile the right sciatic nerve was exposed and bathed in 250 μl of vehicle (sterile dH2O) or 1938 (5 μM in sterile dH2O). Each animal received one vehicle and one compound treatment. The treatments were left on for 30 min prior to washing the bathed nerves with sterile PBS and culling via sodium pentobarbital injection according to local regulations. Nerves were then harvested, washed in fresh 4°C PBS and stored in a fresh vial before snap freezing in liquid nitrogen. Frozen sciatic nerves were homogenised in RIPA buffer supplemented with protease and phosphatase inhibitors using a mortar and pestle homogeniser. The subsequent crude lysates were centrifuged at 10,000xg for 10 min at 4°C, the supernatant harvested and stored at -80°C prior to automated western blot (Wes) analysis for pAkt and controls (Extended Data Fig. 9a; bottom panel).

### Rat sciatic nerve crush injury and 1938 treatment

We used the rat sciatic nerve crush model of peripheral nerve injury and regeneration^82–84^. Adult female Sprague Dawley rats (230-280 g, n=10, Charles River, UK) were anaesthetised by isoflurane inhalation in an induction chamber (5% isoflurane in O2, 0.8 l/min). Anaesthesia was maintained with 1.5-2.5% isoflurane inhalation, and the left sciatic nerve exposed at mid-thigh level.

The nerve was crushed by application of constant pressure using fully closed sterile type 4 tweezers (TAAB) for 15 sec. This was repeated two more times at the same point, with 45° rotation between each crush. The injury site was marked with a 10/0 epineurial non-absorbant suture (Ethicon). Following injury, a single 2 μl injection of 1938 solution (5 μM in sterile H_2_O) or vehicle (sterile dH_2_O) was administered proximal to the crush site with a 10 μl Hamilton syringe. An osmotic minipump (Alzet 1004, Charles River, UK) was also implanted between the muscle layers, adjacent to the nerve oriented with the outlet nearest to the crush site, loaded with 1938 solution (100 μM in sterile H_2_O) or vehicle (sterile H_2_O). Animals were randomly assigned to groups (n=5 per group) and one experimenter was kept blind to condition for conducting functional and histological analyses. Overlying muscle layers were closed using 4/0 sutures (Ethicon) and the skin was closed with wound clips (Clay Adams). Animals were left to recover for 21 days.

### Functional assessment of muscle regeneration

At the end-point of the experiment (21 days), rats were anaesthetised and the sciatic nerve exposed as described above. A reference, ground (Natus) and recording electrode (Ambu Neuroline) were attached above the hip bone, into the tail, and into the tibialis anterior muscle respectively. A microchannel neurointerface (MNI) was placed approximately 2 mm proximal to the injury site and used to stimulate the nerve. The MNI was manufactured using a previously documented protocol^85^. Electrode impedance of the MNI was 27.1 ±19.8 kΩ at 1k Hz. Compound muscle action potential (CMAP) was obtained by sciatic nerve stimulation with square wave pulses of 100 μsec with intensity from 1-10 mA. Stimulus was increased in 0.2 mA steps until muscle response amplitude no longer increased. CMAP amplitude was measured from peak to peak and recorded in triplicate for both the ipsilateral and contralateral side. The CMAP with the largest amplitude was selected for analysis.

A modified multipoint stimulation technique was used to calculate Motor Unit Number Estimation (MUNE)^86–88^. Incremental responses were obtained by delivering a submaximal stimulation of 100 μsec duration at a frequency of 1Hz while increasing the stimulus intensity in increments of 0.02 mA to obtain minimal responses. The initial response was obtained with a stimulus intensity of between 0.21 mA and 0.70 mA. If the initial response did not occur between these stimulus intensities, the stimulating electrode was adjusted to increase or decrease the stimulus intensity as required. Additional Single Motor Unit Potentials (SMUPs) were evoked by stimulation in increments of 0.02 mA to obtain a minimum of four additional increments. The position of the stimulating electrode and the location of the recording electrode was changed to allow the recording of SMUPs from a different site of the muscle. This process was repeated at least three times. The CMAP was divided by the mean magnitude of SMUPs to quantify MUNE.

### Sciatic nerve collection and processing

After electrophysiology recordings, animals were culled with sodium pentobarbital injection according to local regulations. Sciatic nerves, including the common peroneal branch, and tibialis anterior muscles were collected and placed in 4% paraformaldehyde (PFA). Nerve samples were fixed overnight in 4% PFA at 4°C before transferring to PBS. Nerve samples were divided into sciatic nerves including the crush site, and the common peroneal branch for sectioning. Nerve samples were immersed in 30% sucrose overnight at 4°C, then snap frozen in Neg-50 frozen section medium (Thermo Scientific) using liquid nitrogen cooled isopentane. Transverse sections (10 μm) were cut from the distal segment of the common peroneal nerve using a cryostat (HM535, Thermo Scientific). From the sciatic nerve, transverse cryosections (15 μm) were cut from 3 mm and 6 mm distal to the crush site. Sections were adhered to glass slides (Superfrost Plus, Thermo Fisher) for immunofluorescence staining.

For immunofluorescence staining, all washes and dilutions were performed using immunostaining buffer (PBS with 0.002% sodium azide and 0.3% Triton-X 100). Slides were heated to 37°C for 20 min for antigen retrieval and then blocked with 5% normal horse serum for 40 min. Sections were then incubated in primary antibodies overnight at 4°C, followed by incubation for 45 min at room temperature in secondary antibodies. The following antibodies were used: mouse anti-neurofilament (Biolegend #835604, 1:500), goat anti-choline acetyltransferase (Millipore #AB144P, 1:50), DyLight anti-mouse IgG 549 (Vector #DI-2549, 1:300) and DyLight anti-goat IgG 488 (Vector DI-1488, 1:300). Slides were coverslipped with Vectashield Hardset mounting medium (Vector #H-1400).

Fluorescence microscopy (Zeiss AxiolabA1, Axiocam Cm1) was carried out for quantification of motor axons (ChAT) in the distal segment of the common peroneal nerve. For analysis of sciatic nerve sections at 3 mm and 6 mm distal to the crush injury, confocal tile scans (Zeiss LSM 710, 20x magnification) were taken of each transverse section. Quantification of all neurofilament-positive axons was performed using Volocity™ software (Perkin Elmer, Waltham, MA).

### Muscle collection and processing

Tibialis anterior muscles were fixed in 4% PFA for no longer than 15 min and then embedded in Optimal Cutting Temperature (OCT) and snap-frozen on liquid nitrogen-cooled isopentane or left in immunostaining buffer until ready to be processed. Transverse 20 μm cryosections were taken at 300 μm intervals. A minimum of 10 sections from each sample were obtained from the entire cross-section of muscle and adhered to glass slides for immunofluorescence staining.

All washes and dilutions were performed using immunostaining buffer (PBS containing 0.002% sodium azide and 0.3% Triton-X100). Slides were heated to 42°C for 30 min with 20 μg/ml proteinase K and then blocked with 10% goat serum for 40 min at room temperature. After washing, the sections were incubated in primary antibody (neurofilament, Biolegend 835604, 1:500), washed, then incubated with DyLight anti-mouse IgG 488 (Vector #DI-2488, 1:300) and alpha-bungarotoxin (Alexa 594 conjugate, ThermoFisher Scientific, 1:1000). Sections were mounted using Vectashield Hardset mounting medium.

Fluorescence microscopy (Zeiss AxiolabA1, Axiocam Cm1) was used to determine the proportion of motor endplates (α-bungarotoxin) co-stained with neurofilament to quantify the percentage of reinnervated motor endplates. For each sample, a minimum of 20 non-overlapping regions of the entire muscle cross-section were analysed.

For statistical analyses, data from 1938 and vehicle treated animals were compared by unpaired t-tests (Graphpad Prism 8.0.0).

### Statistical methods

The statistical methods for the different types of experiments are included in each experimental section above.

## Extended Data

## Supplementary Material

Nature reporting summary

SI guide with SI tables 2-3, SI figures 1-2 and legends for SI videos 1-4

SI Table 1

SI Table 4

SI Table 5

SI Table 6

SI Table 7

SI Table 8

SI Table 9

SI Video 1

SI Video 2

SI Video 3

SI Video 4

Source data for animal studies

## Data Availability

All raw images for the TIRF experiments are provided via the Open Science Framework (https://osf.io/gzxfm). Mass spectrometry data (raw and processed data) have been deposited to the ProteomeXchange Consortium via the PRIDE partner repository^75^, with the dataset identifier PXD037721. The mass spectrometry proteomics data have been deposited to the ProteomeXchange Consortium via the PRIDE partner repository with the dataset identifier PXD027993. Crystallography data have been deposited in PDB Protein Database^89^ (https://www.rcsb.org/) with the following PDB IDs: 8BFU (apo p110α), 8OW2 (p110α/1938 complex), 7PG5 (apo p110α/p85α) and 7PG6 (BYL719-p110α/p85α). Protein structures used for analysis are available from the PDB database (4JPS, 4ZOP, 4OVV). Protein sequences (*PIK3CA*, *PIK3CB* and *PIK3CD*) are obtained from the UniProt database (https://www.uniprot.org/). The other data that support the findings in this study are available from the corresponding author upon request.

## Code Availability

All macros and R analysis scripts for the TIRF experiments are provided via the Open Science Framework (https://osf.io/gzxfm) Mass spectrometry scripts have been deposited to the ProteomeXchange Consortium via the PRIDE partner repository^75^, with the dataset identifier PXD037721.
