# Supplementary material for: A small molecule PI3Kα activator for cardioprotection and neuroregeneration
Source: Nature. Author manuscript; Available in PMC 2023 Jun 21. (PMC7614683; doi:10.1038/s41586-023-05972-2)
Supplement: SI guide with SI tables 2-3, SI figures 1-2 and legends for SI videos 1-4 [file EMS175910-supplement-SI_guide_with_SI_tables_2_3__SI_figures_1_2_and_legends_for_SI_videos_1_4.pdf]

A small molecule PI3Kα activator for cardioprotection and neuroregeneration

Grace Q Gong[1], Benoit Bilanges[1], Ben Allsop[2], Glenn R Masson[3,4], Victoria Roberton[5], Trevor Askwith[2], Sally Oxenford[2], Ralitsa R Madsen[1], Sarah E Conduit[1], Dom Bellini[3], Martina Fitzek[6], Matt Collier[6], Osman Najam[7], Zhenhe He[7], Ben Wahab[8], Stephen H McLaughlin[3], AW Edith Chan[9], Isabella Feierberg[10], Andrew Madin[11], Daniele Morelli[1], Amandeep Bhamra[12], Vanesa Vinciauskaite[4], Karen E. Anderson[13], Silvia Surinova[12], Nikos Pinotsis[14], Elena Lopez-Guadamillas[1], Matthew Wilcox[5], Alice Hooper[2], Chandni Patel[2], Maria A Whitehead[1], Tom D Bunney[15], Len R Stephens[13], Phillip T Hawkins[13], Matilda Katan[15], Derek M Yellon[7,#], Sean M Davidson[7,#], David M Smith[16,#], James B Phillips[5,#], Richard Angell[2,8,#], Roger L Williams[3,#], & Bart Vanhaesebroeck[1]✉

[1]Cell Signalling, Cancer Institute, University College London, UK.
[2]Drug Discovery Group, Translational Research Office, University College London, London, UK.
[3]Medical Research Council Laboratory of Molecular Biology, Cambridge, UK.
[4]Division of Cellular Medicine, School of Medicine, University of Dundee, UK.
[5]UCL Centre for Nerve Engineering, UCL School of Pharmacy, University College London, London, UK.
[6]Hit Discovery, Discovery Sciences, R&D, AstraZeneca, Alderley Park, Cheshire, UK.
[7]The Hatter Cardiovascular Institute, University College London, London, UK.
[8]Medicines Discovery Institute, School of Biosciences, Cardiff University, Cardiff CF10 3AT, UK.
[9]Wolfson Institute for Biomedical Research, University College London, London, UK.
[10]Molecular AI, Discovery Sciences, R&D, AstraZeneca, Waltham MA, USA.
[11]Hit Discovery, Discovery Sciences, R&D, AstraZeneca, Cambridge, UK.
[12]Proteomics Research Translational Technology Platform, Cancer Institute, University College London, London, UK.
[13]Signalling Programme, Babraham Institute, Cambridge, UK.
[14]Institute of Structural and Molecular Biology, Birkbeck College, London, UK.
[15]Institute of Structural and Molecular Biology, Division of Biosciences, University College London, London, UK.
[16]Emerging Innovations, Discovery Sciences, R&D, AstraZeneca, Cambridge, UK.

[#]*Joint senior authors*
✉*Corresponding author* e-mail: bart.vanh@ucl.ac.uk

Table of contents	
**Supplementary Table 1**	HDX Data and analysis
**Supplementary Table 2**	Crystallography data collection & refinement statistics
**Supplementary Table 3**	Structure-activity relationship analysis of 1938 analogues
**Supplementary Table 4**	1938 Thermo Fisher SSBK-Adapta Screen
**Supplementary Table 5**	1938 Thermo Fisher SSBK-LanthaScreen Binding
**Supplementary Table 6**	1938 Thermo Fisher SSBK-Z'-LYTE Screen
**Supplementary Table 7**	MEF Phosphoproteomic analysis of 1938 and insulin signalling
**Supplementary Table 8**	MEF Phosphoproteomics sites represented in PhosphoSite
**Supplementary Table 9**	Geometry of 1938 checked against crystallographic database (CSD) using MOGUL
**Supplementary Figure 1**	Raw uncropped gels of western blots
**Supplementary Figure 2**	Gating strategy for flow cytometry analysis shown in Fig. 5b.
**Supplementary Video 1**	Mechanisms of activation by 1938
**Supplementary Video 2**	Representative TIRFM time-lapse videos of WT A549 cells treated with vehicle
**Supplementary Video 3**	Representative TIRFM time-lapse videos of WT A549 cells treated with 1938
**Supplementary Video 4**	Representative TIRFM time-lapse videos of KO A549 cells treated with 1938
**Legends for Supplementary files**	Legends for Supplementary Figures 1-2, and Supplementary Videos 1-4.

Supplementary Table 2a: Crystal data collection and refinement statistics for p110α/p85α niSH2 construct

	PI3Kα	PI3Kα + BYL719
**Data Collection**		
Beamline	I03 (DLS)	P13 (EMBL/PetraIII)
Wavelength (Å)	0.97625	0.97626
Resolution Range (Å, grad)	136.09 – 2.20 (2.25 – 2.20)	49.04 – 2.50 (1.65 – 2.50)
Space group	$P\ 2_1\ 2_1\ 2_1$	$P\ 2_1\ 2_1\ 2_1$
Cell parameters a, b, c (Å)	105.43, 105.24, 136.09	104.97, 105.19 135.74
Total reflections	606,367 (24,058)	704,489 (113,969)
Unique reflections	70,889 (4,522)	52,624 (8,343)
Multiplicity	7.8 (5.3)	13.4 (13.7)
Completeness (%)	100.0 (99.6)	99.9 (99.3)
Mean I/Sigma(I)	14.0 (1.0)	13.48 (0.98)
Wilson B-factor (Å^2)	54.84	72.6
R_{meas} (%)	8.0 (184.0)	21.7 (300.5)
$CC_{1/2}$	0.999 (0.398)	0.998 (0.469)
**Refinement**		
R_{work}/R_{free} (%)	19.64 / 24.56	18.73 / 24.03
Protein atoms	10,619	10,637
Solvent molecules	152	59
Other atoms	16 (1xGOL, 1xPO_4^{3-})	30 (1xNa^+, 1xBYL719)
B-factor (Å^2)		
Protein (p110a)	64.85	71.25
Protein (p85a)	84.05	95.10
Solvent	55.55	58.41
Other	78.37	62.45
Ramachandran Plot		
Favoured (%)	96.82	98.29
Allowed (%)	2.95	1.71
Outliers (%)	0.23	0.00
Clash score	6.00	7.40
Rmsd		
Bonds (Å)	0.009	0.009
Angles (grad)	1.145	1.261
PDB code	7PG5	7PG6

Supplementary Table 2b: Crystal data collection and refinement statistics for p110α only construct

	**p110α**	**p110α+1938**
**Data collection**		
Space group	P 21 21 21	P 21 21 21
Cell dimensions		
a, *b*, *c* (Å)	58.2884 135.256 142.67	58.72 134.88 144.77
α, β, γ (°)	90 90 90	90 90 90
Resolution (Å)	68 - 2.41 (2.45 - 2.41)	135 - 2.57 (2.61 - 2.57)
R_{merge}	0.08 (3.1)	0.13 (2.5)
CC1/2	0.99 (0.4)	0.99 (0.3)
I/σ(I)	17.3 (0.5)	11.8 (1.2)
Completeness (%)	100 (100)	100 (100.00)
Multiplicity	13.5 (14.1)	8.7 (8.9)
Total observations	603001 (30855)	324987 (16279)
Unique observations	44504 (2192)	37543 (1832)
**Refinement**		
Number of reflections	44384 (2707)	37468 (2856)
R_{work} / R_{free}	0.24/ 0.28	0.21 / 0.27
Ramachandran favored (outliers) (%)	96.31 (0.00)	95.48 (0.00)
Clashscore	10.87	4.77
Rotamer outliers (%)	0.74	0.37
No. atoms		
Protein	7186	7306
Ligand/ion	N/A	34
Water	51	36
B-factors		

Protein	90	83
Water	76	58
R.m.s. deviations		
Bond lengths (Å)	0.004	0.002
Bond angles (°)	0.77	0.58

Supplementary Table 3: Structure-activity relationship analysis of 1938 analogues

Compound	Structure	EC_{50} (μM)	Maximum activity relative to pY control (%)	Maximum activity relative to 1938 (%)
1938	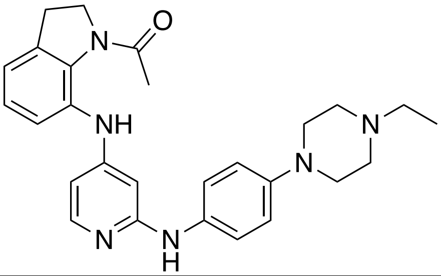	58 ± 28	397 ± 60	100
1887	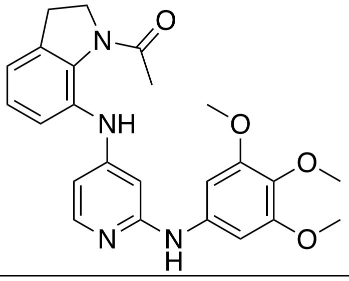	36 ± 5	318 ± 68	80
1889	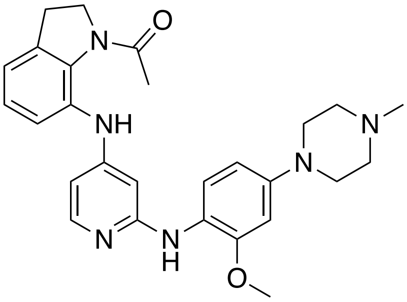	56 ± 24	408 ± 110	102
2016	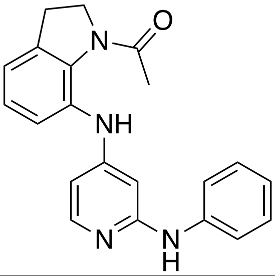	NA	36 ± 8	9
2106	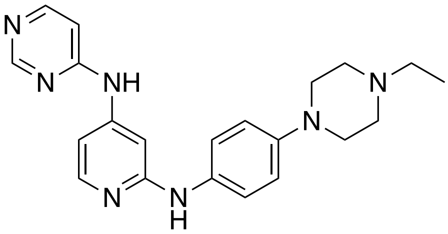	NA	14 ± 13	4
2152	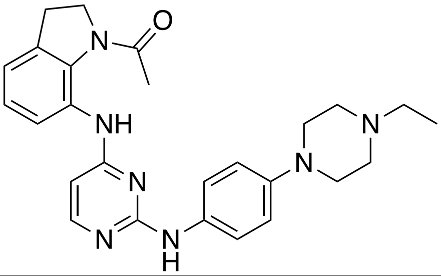	NA	16 ± 8	4

Replacement of the core pyridine for a pyrimidine (UCL-TRO-2152) results in >95% reduction in activity, consistent with the proposal that the equivalent nitrogen is no longer protonated at physiological pH and unable to form the key interaction with D603. UCL-TRO-1887 and 1889 have activities comparable with 1938, indicating that modification or replacement of the piperazine can be tolerated. However, complete removal of the piperazine (UCL-TRO-2016) reduces activity by more than 90%. UCL-TRO-2016 is also less soluble than 1938. The crystal structure shows that the piperazine points out towards solvent, suggesting that presence of the piperazine or tri-O-methyl substituted phenyl may be important in displacing water molecules and maintaining hydrophobic interactions with L1006 and F1016. The acetylindoline is required for edge to face and hydrophobic interactions with F1016 and L1006. The carbonyl group of the acetyl makes an internal hydrogen bond with the NH linking the indoline and pyridine, holding the indoline in an orientation suitable for interacting with F1016. Replacement of the acetylated indoline with a pyrimidine (UCL-TRO-2106) reduces activity by more than 95%, potentially due to less favourable edge to face interactions with F1016.

Supplementary Figure 1

Raw uncropped for Figure 3e

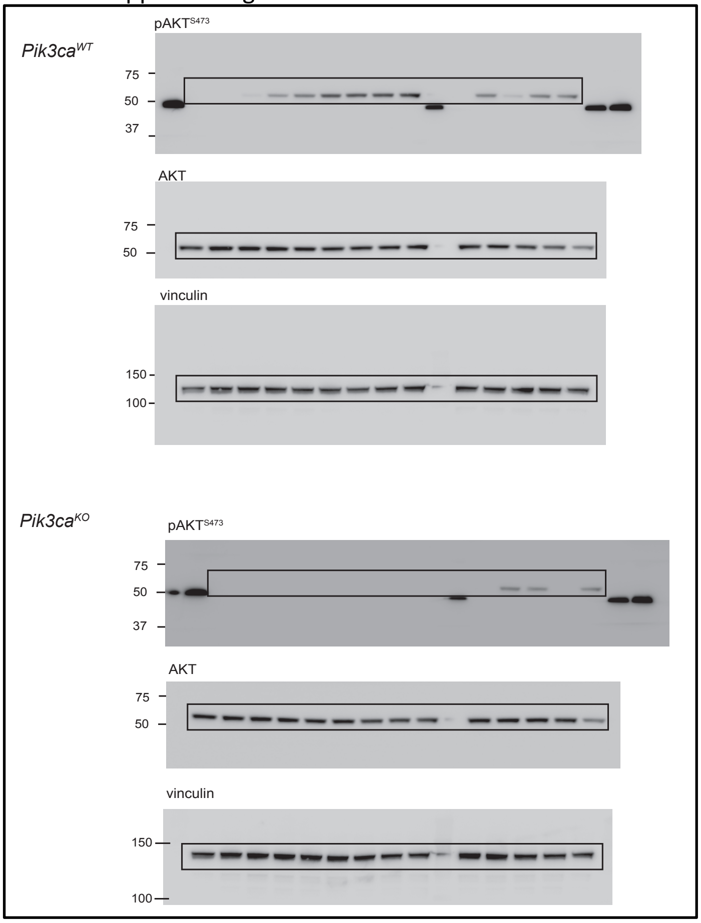

Raw uncropped for Extended Data Figure 3d

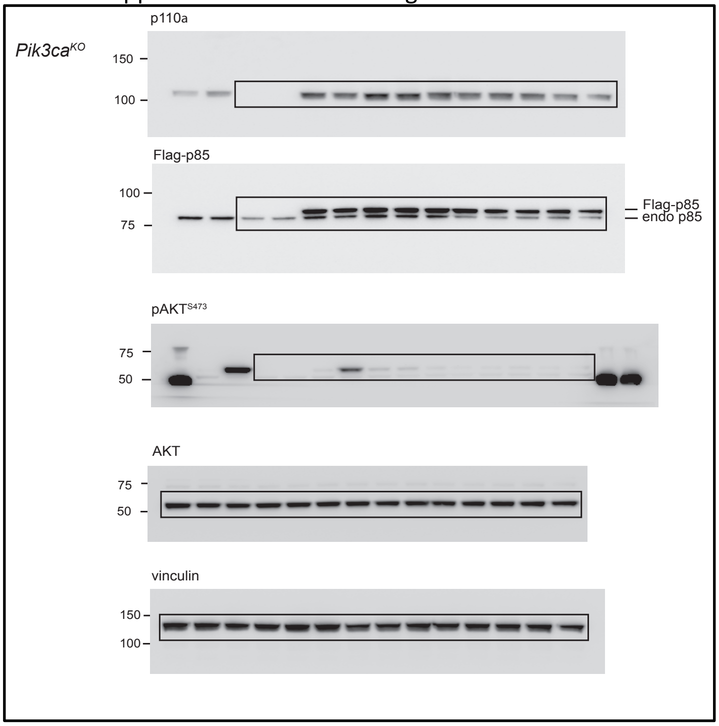

Raw uncropped for Extended Data Figure 3e

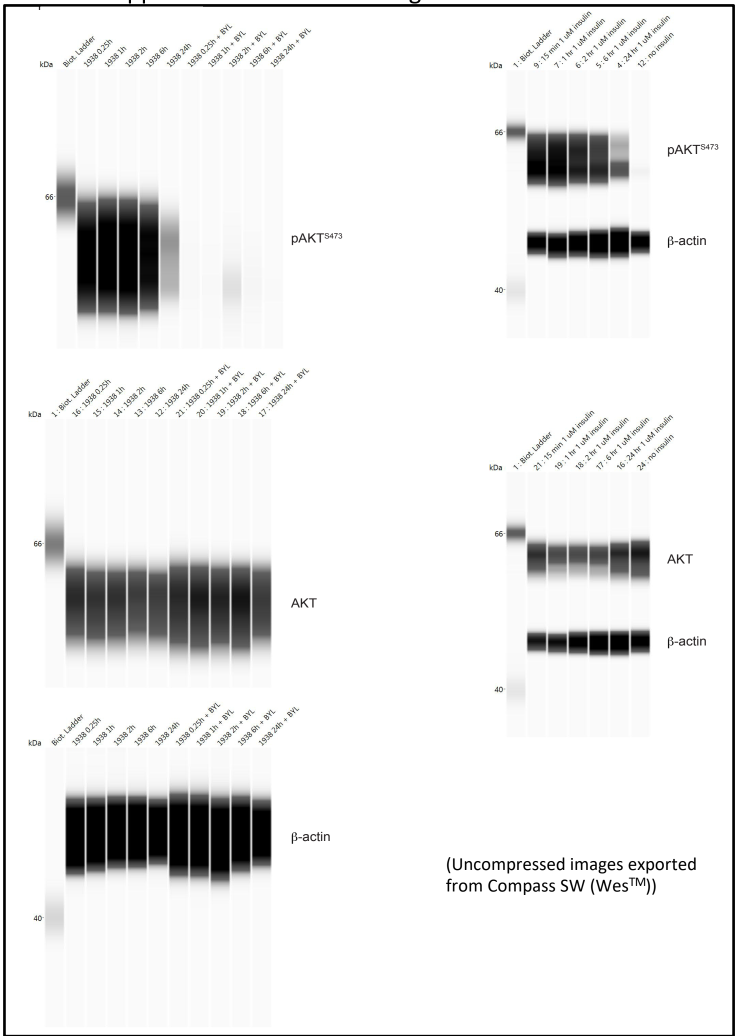

Raw uncropped for Extended Data Figure 3g

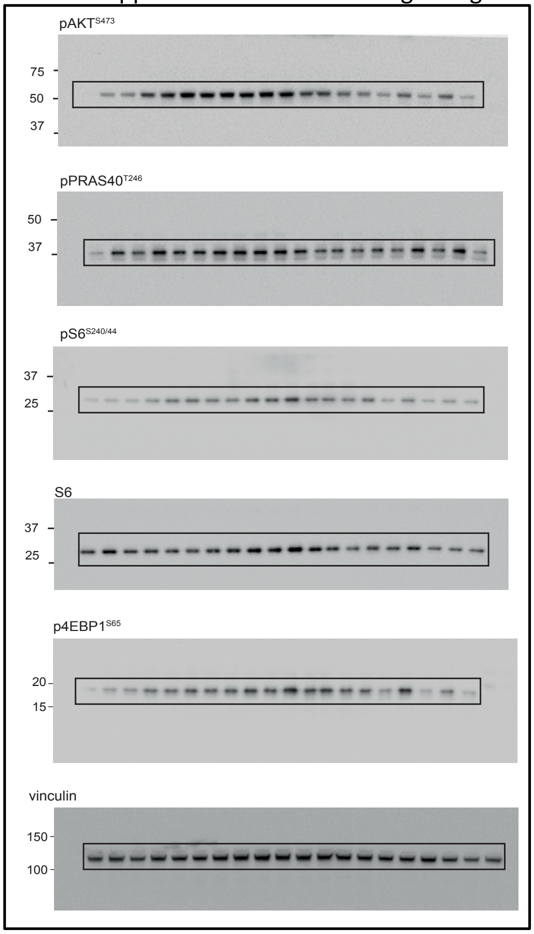

Supplementary Figure 2

Gating strategy for flow cytometry analysis shown in Figure 5b: example from one experiment

Reagent used: EdU Flow Cytometry Kit 488 (SIGMA cat#BCK-FC488)

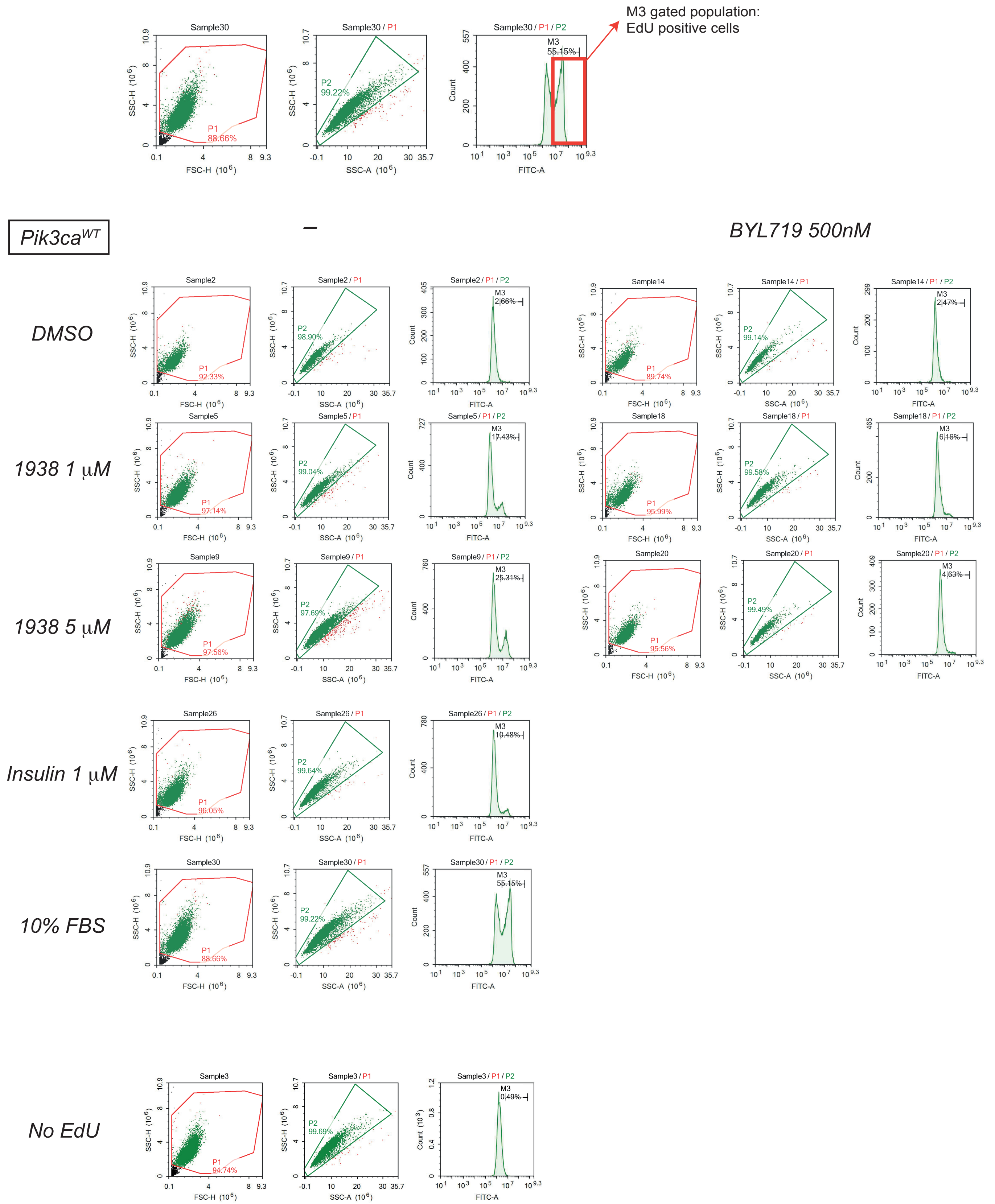

Legends for Supplementary Information

Supplementary Figure 1: Raw uncropped gels of western blots for Figure 3e, Extended Data Figure 3d, Extended Data Figure 3e and Extended Data Figure 3g.

Supplementary Figure 2: Gating strategy for flow cytometry analysis shown in Figure 5b.

Supplementary Video 1: A cartoon representation for the conformational changes elicited in p110α upon 1938 binding. The movie was rendered as in Figure 2d. For the movie, the 1938 compound shown as magenta spheres is invisible in the apo state and appears in the bound state. The yellow spheres mark the sites of cancer-associated mutations from the COSMIC data base that are near the 1938-binding site (only mutations with greater than 10 reports are shown). The kinase domain is colored salmon, the helical domain is pale green and the C2 domain is cyan. The regions of the helical domain showing decreased HDX-MS protection for the common helical domain mutations are colored orange. The PRD-like helix is coloured dark purple. PIP_2 substrate (slate, ball and stick model) has been modelled in the active based on 4OVV. A region of the activation loop (colored slate) has been taken from 7PG5 since it is disordered in the 1938-bound structure. This region is in a thick worm representation. The two slate spheres represent two residues important for PIP_2 recognition (K942 and R949). The three chocolate spheres in the kinase domain represent three residues that are essential for the phosphate transfer (K776, H917 and H936). A bound ATP (blue) has been modelled based on PDB ID 1E8X. The ATP binding loop is coloured yellow. Phosphates in PIP_2 and ATP are shown in red.

Supplementary Video 2: Representative TIRFM time-lapse videos of wild-type A549 cells expressing the GFP-tagged PH-ARNO-I303Ex2 (ARNO) PIP_3 biosensor treated with vehicle prior to addition of BYL719. Individual treatments are indicated in the bottom left corner of the video. Time stamps are included in the top left corner. The entire 2h experimental time course is shown at a speed of 3 frames per sec. Individual frames were acquired at 3 min intervals as specified in Materials and Methods. Scale bar = 11 µm.

Supplementary Video 3: Representative TIRFM time-lapse videos of wild-type A549 cells expressing the GFP-tagged PH-ARNO-I303Ex2 (ARNO) PIP_3 biosensor, treated with 1938 prior to addition of BYL719. Individual treatments are indicated in the bottom left corner of the video. Time stamps are included in the top left corner. The entire 2h experimental time course is shown at a speed of 3 frames per sec. Individual frames were acquired at 3 min intervals as specified in Materials and Methods. Scale bar = 11 µm.

Supplementary Video 4: Representative TIRFM time-lapse videos of *PIK3CA*-KO A549 cells expressing the GFP-tagged PH-ARNO-I303Ex2 (ARNO) PIP_3 biosensor, treated with 1938 prior to addition of BYL719. Individual treatments are indicated in the bottom left corner of the video. Time stamps are included in the top left corner. The entire 2h experimental time course is shown at a speed of 3 frames per sec. Individual frames were acquired at 3 min intervals as specified in Materials and Methods. Scale bar = 11 µm.
